# E2F7 and E2F8 attenuate diabetic kidney disease by suppressing E2F1-induced tubular senescence

**DOI:** 10.1038/s42003-026-11334-1

**Published:** 2026-11-13

**Authors:** Zhiyang Li, Yunli Dai, Dan Liang, Yawen Xiao, Li Hu, Xin Wu, Zhaowei Feng, JiaJia Chen, Shengjie Chen, Zhiping Yuan, Mingjun Shi, Yuanyuan Wang, Lingling Liu, Pengchen Chen, Shihai Jiang, Bing Guo, Ying Xiao

**Affiliations:** 1 Guizhou Provincial Key Laboratory of Pathogenesis and Prevention of Common Chronic Diseases, Guizhou Medical University, Guiyang, China; 2 Institute of Laboratory Medicine, Clinical Chemistry and Molecular Diagnostics, Universitätsklinikum Leipzig, Universität Leipzig, Leipzig, Germany; 3 International Scientific and Technological Cooperation Base of Pathogenesis and Drug Research on Common Major Diseases, Guizhou Medical University, Guiyang, China; 4 Guizhou Province Talent Base of Research on the Pathogenesis and Drug Prevention and Treatment for Common Major Diseases, Guizhou Medical University, Guiyang, Guizhou China; 5 The Second People’s Hospital of Guiyang, Guiyang, China; 6 University Town Hospital, Guizhou Medical University, Guiyang, China; 7 Collaborative Innovation Center for Prevention and Control of Endemic and Ethnic Regional Diseases Co-constructed by the Province and Ministry, Guizhou, China

**Keywords:** Diabetes complications, Chronic kidney disease, Mechanisms of disease

## Abstract

Targeting cellular senescence represents a critical frontier for mitigating diabetic kidney disease (DKD), yet the transcriptional hierarchies governing renal senescence remain incompletely defined. While the E2F family is pivotal in cell cycle regulation, its specific contribution to the senescent phenotype in DKD is unclear. Here we show that resveratrol attenuates renal fibrosis and cellular senescence in *db/db* mice by modulating the interaction between activator and repressor E2f transcription factors. We demonstrate that hyperglycemic conditions drive the upregulation of E2f1 in mouse renal tubular epithelial cells, directly precipitating DNA damage and senescence. Accordingly, in vivo knockdown of E2f1 significantly ameliorates DKD pathology. Mechanistically, resveratrol induces the expression of the atypical repressors E2f7 and E2f8. These repressors directly bind to the *E2F1* promoter region to repress its transcription, thereby abrogating E2f1-mediated cellular senescence. These findings elucidate a transcriptional axis linking resveratrol to the suppression of renal senescence and highlight the E2F1-E2F7/8 regulatory loop as a promising therapeutic target.

## Abstract (web summary)

Resveratrol reduces kidney fibrosis and cellular senescence in diabetic kidney disease by suppressing E2F1 through induction of E2F7/8 repressors. Targeting this E2F1-E2F7/8 regulatory axis may offer a promising strategy to slow DKD progression.

## Introduction

Diabetic kidney disease (DKD) constitutes the leading cause of end-stage renal disease (ESRD) worldwide, imposing a significant burden on global healthcare systems^1^. The pathogenesis of DKD is complex, characterized structurally by glomerular sclerosis and tubulointerstitial fibrosis, and functionally by a progressive decline in filtration capacity. Emerging evidence has identified cellular senescence, a state of irreversible cell cycle arrest accompanied by a senescence-associated secretory phenotype (SASP), as a pivotal driver of diabetic nephropathy^2^. In particular, the accumulation of senescent renal tubular epithelial cells (RTECs) under hyperglycemic conditions fosters a pro-inflammatory and pro-fibrotic microenvironment, accelerating renal tissue deterioration^3^. Consequently, targeting the molecular machinery governing cellular senescence represents a promising therapeutic frontier for halting DKD progression.

E2F family of transcription factors are one of the central to the regulation of cell proliferation and cell fate decisions^4^. While classically recognized for coordinating the G1/S transition in the cell cycle, the E2F family exhibits a functional dichotomy that extends into stress responses and aging^5^. The family is generally categorized into canonical activators (E2F1-3) and repressors (E2F3b-8). Among these, E2F1 is a potent activator that, paradoxically, can induce apoptosis or senescence in response to DNA damage signals^6^. Conversely, the atypical repressors E2F7 and E2F8 have emerged as critical regulators of E2F1-dependent transcription, functioning independently of the retinoblastoma (Rb) protein to maintain genomic stability^7^. Despite this, the precise interplay between activating and repressing E2F members in the context of metabolic stress and renal fibrosis remains unclear^8^. Specifically, whether the dysregulation of the E2F axis drives the senescent phenotype in DKD is a critical gap in current understanding of renal pathophysiology^9^.

Resveratrol (3,5,4’-trihydroxystilbene), a natural polyphenol, has garnered attention for its capacity to modulate aging processes and ameliorate metabolic disorders. Previous studies indicate that resveratrol exerts renal protective effects by suppressing oxidative stress and fibrosis^10^. However, the molecular mechanisms linking resveratrol to cell cycle regulators in the diabetic kidney remain elusive^11^. Based on the bioinformatics analysis results, we hypothesized that resveratrol may mitigate glucose-induced senescence by restoring the homeostatic balance within the E2F family.

In this study, we elucidate a transcriptional mechanism governing renal senescence in DKD. We demonstrate that resveratrol attenuates renal fibrosis and RTECs senescence in *db/db* mice by differentially regulating E2f isoforms. We found that hyperglycemic conditions trigger the upregulation of E2f1 in mouse RTECs, which drives DNA damage and senescence, while the simultaneous overexpression of the atypical repressors E2f7 and E2f8 is sufficient to abrogate these pathological features. Mechanistically, we show that E2f7 and E2f8 directly bind to *E2f1* promoter-binding domain, thereby inhibiting its transcriptional activity. These findings indicate a possible mode of E2F regulation and highlight the therapeutic potential of targeting the E2F7/8-E2F1 axis to prevent the progression of diabetic kidney disease.

## Methods

### Cell culture and treatments

Mouse renal tubular epithelial cells (mRTECs) were purchased from Otwo Biotech Inc. (cat# HTX2460). Cells were cultured in standard medium (DMEM low glucose; Gibco cat#11885084. 10% fetal bovine serum (FBS); Gibco, cat#A5670201) and in a humidified atmosphere containing 5% CO₂. For experimental treatments, cells were maintained in either normal glucose medium (NG; 5.5 mmol/L glucose) or high glucose medium (HG; 30 mmol/L glucose, Glucose Solution; Gibco, cat# A2494001) to mimic hyperglycemia conditions^9^. Cultured mRTECs were incubated in 30 mM glucose for 72 h which was suggested by previous study^12^. We selected these parameters based on prior literature and our preliminary in vitro data, demonstrating that this specific high-glucose regimen robustly induces nuclear p21 expression without significantly compromising cell viability (Figure S2b, c). For resveratrol treatments concentration, 15 μM resveratrol was used in the study and was determined by cell survival assay on mRTECs (Figure S2a).

### Construction of expression plasmid and transient transfection

The mRTECs were transiently transfected in 6-well plates using Lipofectamine^TM^ 3000 (Invitrogen; Cat# L3000015) following the manufacturer’s protocol. Transfection complexes were prepared in Opti-MEM (Gibco; Cat#31985062) and added to cells at 80% confluence. After a 6 h incubation, the medium was replaced with fresh complete medium, and cells were cultured for 48 h before downstream analyses. Overexpression plasmid including E2f7 expression plasmid, E2f8 expression plasmid, and *E2f1* promoter plasmid were purchased from Shanghai Yile Biotechnology Co., Ltd. The E2f7 and E2f8 siRNA were purchased from Suzhou Genepharma Biotechnology Co., Ltd.

### Ethics statement, animal models, and experimental design

All animal procedures were approved by the Guizhou Medical University Animal Care Welfare Committee (Animal Experimental Ethical Approve Number: 2200406, Date: 2022.03.01) and conducted in accordance with ARRIVE guidelines. Males were used in this study to reduce the effects of estrogen on metabolic fluctuations, especially in obesity animal models. Male *db/db* mice (Strain: BSK-DB, Strain No.: T002407; 7 weeks old, 40 ± 5 g, *n* = 36) and age-matched non-diabetic *db/m* control mice (20 ± 2 g, *n* = 12) were obtained from Jiangsu Jicui Yaokang Biotechnology Co., Ltd. Mice were housed under specific pathogen-free (SPF) conditions at 24–26  C with humidity ranging between 30 and 60% with 12 h light/dark cycles switching at 6 p.m, and with free access to food and water. Two or one mice were housed in per cage. Humane endpoints were defined as > 20% body weight loss or the occurrence of severe distress requiring euthanasia. There was one mouse that reached humane endpoints therefore euthanized during the study.

All animals were grouped randomly and blindly by researchers. Till mice reach 16 weeks of age, all *db/db* mice were randomly assigned to experimental groups (PBS group, *n* = 8; resveratrol group, *n* = 7) using computer-generated random numbers. In all mice experiments, each experimental group number was determined by G-power sample size calculation which was considered sufficient to detect biologically meaningful differences while adhering to the 3Rs principle in the *db/db* model. Allocation concealment was maintained by assigning animal ID codes prior to treatment. The resveratrol treatment group mice were administered resveratrol (MedChemExpress; Cat# HY-1656) by gavage at a dose of 10 mg/kg, six days per week for a duration of eight weeks according to previous protocol with slightly modification^13,14^. At the conclusion of the experiment, 2 mice died from hypoglycemia and 1 mouse died from cannibalism therefore excluded from experimental calculation.

To induce gene silencing in vivo, 8-week-old *db/db* mice received tail vein injections of adeno-associated virus (AAV) -shE2f1 plasmid (Obio Technology Shanghai Corp. Ltd; Cat# HYKY-210407032-DAAV-2021AAV1) vectors encoding an E2f1-targeting shRNA (AAV-shE2F1, *n* = 8, 100 μL, titer of 5 × 10¹² viral genomes (vg)/mL), resulting in a total dose of 5 × 10¹¹ vg per mouse. Control group processed same tail vein injections with an equivalent dose of an AAV vector encoding a non-targeting shRNA (*n* = 8). Animals were randomly assigned to experimental groups using computer-generated random numbers. Allocation concealment was maintained by assigning animal ID codes prior to treatment. At the end of the experiment, 2 mice died during tail vein injection, 1 mouse died from hyperglycemia, and 1 mouse reached these humane endpoints therefore euthanized during the study. For anesthesia, mice were anesthetized via an intraperitoneal (i.p.) injection of a mixture containing ketamine (100 mg/kg, Bela Pharm) and xylazine (10 mg/kg, Rompu). For euthanasia, mice were euthanized by carbon dioxide inhalation followed by cervical dislocation.

The primary outcome was urinary albumin content, while secondary outcomes included morphological pathological alterations in animal kidneys, as demonstrated by hematoxylin and eosin staining, periodic acid-Schiff staining, and the deposition of aging markers in the kidneys. The personnel conducting the animal experiments were aware of the grouping. However, histological staining and data analysis were conducted by independent researchers blinded to group identity.

### Serum biochemical analysis

Following a 6 h fasting period, mice were anesthetized, and blood samples were collected via the retro-orbital sinus. Serum was isolated by centrifugation for biochemical profiling. Levels of blood glucose (BG), blood urea nitrogen (BUN), total cholesterol (T-CHO), and triglycerides (TG) were quantified using commercial kits (Nanjing Jiancheng Bioengineering Institute; Cat# A154-1-1, A028-2-1, A111-1-1, and A110-1-1, respectively). Microalbuminuria (MAU) was measured using an ELISA kit (Elabscience; Cat# E-EL-M0792) according to the manufacturer instructions.

### Histopathology and immunohistochemistry

Kidney tissues were harvested, fixed, embedded in paraffin, and sectioned. Histological assessments were performed using commercial staining kits (Solarbio) according to the manufacturer’s protocols. These included Senescence-Associated β-Galactosidase (SA-β-gal, Cat# G1580), Hematoxylin and Eosin (H&E, Cat# G1120), Masson’s Trichrome (Cat# G1340), and Sirius Red (Cat# G1472) staining according to the manufacturer instructions.

For immunohistochemistry (IHC), tissue sections underwent deparaffinization, antigen retrieval, and blocking of endogenous peroxidases. Sections were incubated with primary antibodies E2F7 (Proteintech, 230073E3), E2F8(Abcam, ab109596), and E2F1 (Abcam, ab112580) overnight at 4 °C, followed by incubation with appropriate secondary antibodies. Signals were visualized using 3,3’-diaminobenzidine (DAB), and nuclei were counterstained with hematoxylin. Images were acquired using an optical microscope (Olympus BX43).

### Quantity real-time polymerase chain reaction

Total RNA was extracted from mice renal cortex using TRIzol reagent (ThermoFisher; Cat#15596018) and reverse transcribed into complementary DNA (cDNA). *E2f1*, *E2f7*, *E2f8*, and *β-actin* were amplified using cDNA as the template. The relative mRNA expression was calculated with the 2 − ΔΔCt method. The primer sequences list was listed in Supplementary Table 1.

### Enzyme-linked immunosorbent assay (ELISA)

Mouse CCL2/MCP-1 levels were quantified using the Mouse CCL2/JE/MCP-1 Quantikine ELISA Kit (R&D Systems; Cat# MJE00B) according to the manufacturer instructions. Briefly, samples and standards were added to microplate wells containing assay diluent, followed by incubation with an MCP-1 conjugate. Substrate and stop solutions were sequentially added, and the optical density was measured at 450 nm using a CLARIOstar Plus microplate reader (BMG LABTECH) with 620 nm correction.

### Flow cytometry of cell cycle analysis

Cells (1 × 10^6^) were fixed in ice-cold 70% ethanol and washed with PBS. Fixed cells were stained with a propidium iodide (PI)/RNase A working solution (Cell Cycle Detection Kit, KeyGen Biotech; Cat# KGA511-KGA512) for 1 h in the dark. Fluorescence signals were acquired on a NovoCyte flow cytometer (Agilent), with PI emission detected following 488 nm excitation.

### Cellular thermal shift assay (CETSA)

The mRTECs grown in 10 cm dishes were lysed, and the resulting lysates were divided into two equal aliquots (500 µL each). Lysates were treated with either 0.4 nM resveratrol (Res) or 1% (v/v) DMSO (vehicle control) and incubated for 1.5 h with agitation at 600 rpm. Samples were then subjected to a temperature gradient (37, 42, 47, 52, 57, and 62 °C), with a 5-min reaction time at each temperature. Following heat treatment, tubes were immediately chilled on ice to quench the thermal reaction. The thermal stability of E2F7 and E2F8 was subsequently analyzed by western blotting to show remaining protein expression.

### E2F7 and E2F8 ubiquitination assay

Following the treatment of mRTECs with resveratrol under normal or high-glucose conditions, cell lysates were subjected to co-immunoprecipitation. Lysates were incubated with antibodies against E2F7 (Proteintech, 24489-1-AP) or E2F8 (Abcam, ab109596), alongside isotype-matched IgG controls. Input and immunoprecipitated fractions were subsequently resolved and analyzed for ubiquitination status by western blotting.

### Western blotting

Total protein was extracted from kidney tissues or cultured cells using RIPA lysis buffer containing protease and phosphatase inhibitors. Protein lysates were separated by SDS-PAGE and transferred onto PVDF membranes. Membranes were blocked and incubated with specific primary antibodies (detailed in Suppl. Table 2), followed by HRP-conjugated secondary antibodies. Protein bands were visualized using enhanced chemiluminescence (ECL) and quantified using ImageJ software (ImageJ 1.54p).

### Co-Immunoprecipitation (Co-IP)

The mRTECs were transiently transfected with Flag-E2F8 and/or HA-E2F7 expression plasmids. Seventy-two hours post-transfection under normal glucose conditions, cells were washed with PBS and lysed in ice-cold IP-grade RIPA buffer (ThermoFisher, Cat# 26149) for 10 minutes. Lysates were centrifuged to remove debris, and the supernatant was divided into Input, IgG control, and IP fractions. Samples were incubated overnight at 4 °C with gentle rotation in the presence of either anti-Flag antibody or a control IgG. Immunocomplexes were captured, washed, and boiled in SDS loading buffer prior to Western blot analysis.

### Chromatin immunoprecipitation (ChIP)

ChIP assays were performed using the SimpleChIP® Enzymatic Chromatin IP Kit (Magnetic Beads) (Cell Signaling Technology; Cat# 9003) following the manufacturer standard procedures. Briefly, cells were cross-linked with 1% formaldehyde and lysed in an SDS buffer containing protease inhibitors. In the case of fresh tissue, the tissue was placed in lysis buffer and homogenized using a homogenizer. Chromatin was fragmented via micrococcal nuclease digestion and sonication. Diluted lysates were incubated overnight at 4 °C with an anti-E2F7 (Proteintech, 24489-1-AP) or E2F8 antibody (Abcam, ab109596), alongside isotype-matched IgG controls, followed by a 2 h incubation with Protein G magnetic beads. After washing and elution, cross-links were reversed at 65 °C for 4 h. Following DNA purification, ChIP samples were analysed by qPCR using primers targeting *E2f1* promoter regions containing putative binding sites. Primer sequences are provided in Supplementary Table 1.

### Dual-luciferase reporter assay

The promoter region of *E2f1* was cloned into the pGL3-Basic vector (Shanghai Leyi Biotechnology Co., Ltd.) to generate the pGL3-*E2f1* reporter construct. High-purity plasmid DNA was isolated using an endotoxin-free plasmid purification kit (PureLink, Invitrogen; Cat# K211004A). The mRTECs were seeded in 12-well plates and co-transfected with the pGL3-*E2f1* plasmid and a *Renilla* luciferase control vector (pRL-TK) to normalize for transfection efficiency.

Cells were lysed 48 h post-transfection using Passive Lysis Buffer (Promega; Cat#E1941). Firefly and *Renilla* luciferase activities were measured sequentially using the Dual-Luciferase® Reporter Assay System (Promega; Cat#E1910). The relative transcriptional activity of the *E2f1* promoter was calculated as the ratio of Firefly to *Renilla* luciferase signals.

### ChIP-Atlas analysis of E2F1 target genes

To investigate *E2F1* directly binding genes promoter within the ATM signaling axis, aggregated ChIP-seq datasets were analyzed via the ChIP-Atlas database (https://chip-atlas.org/)^14^. The database was queried for the E2F1 antigen against the *Homo sapiens* reference genome (hg38). Putative binding sites were restricted to proximal promoter regions, defined as ±1 kilobase (kb) from the transcription start site (TSS). To identify high-confidence regulatory targets, a stringent E2F1 binding score threshold of ≥450 was applied. The resulting gene list was subsequently cross-referenced to isolate established components of the ATM signaling cascade.

### Nephroseq database analysis

The mRNA expression levels of *E2F1* in human kidney tissues were analyzed using the publicly available Nephroseq database (http://www.nephroseq.org, v5, University of Michigan, Ann Arbor, MI). Specifically, the ERCB Nephrotic Syndrome dataset was selected to compare the expression of *E2F1* between patients with DKD and healthy control. A Student’s t-test was used for the comparison via database. The Schmid Diabetes dataset was utilized to compare patient clinical estimated glomerular filtration rate (eGFR). The correlation was evaluated using Spearman’s correlation analysis with a *P*-value < 0.05 considered statistically significant within database. Because the data analyzed in this study were obtained from a publicly available database, ethical approval and patient consent were not required.

### Human DKD kidney biopsies

Kidney biopsies were obtained from one patient with DKD and one control individual at the First Affiliated Hospital of Guizhou Medical University. Tissues were embedded in paraffin for following section staining. All ethical regulations relevant to human research participants were followed and therefore study did not relate to any interventional protocols in human subjects. The study was approved by the Institutional Ethics Committee of Guizhou Medical University (2026-158), and written informed consent was obtained from participants.

### Statistics and reproducibility

Statistical analyses were performed using SPSS software (version 25.0; IBM Corp., Armonk, NY, USA). Normality of data distribution was assessed using the Shapiro-Wilk test. Data are presented as mean ± standard deviation (SD), and ‘*n*’ indicates the number of experiment replicates or the number of mice, are provided in the figure legends. Differences were calculated by unpaired two-tailed Student’s t-tests in two groups, and among multiple groups were analyzed using one-way analysis of variance (ANOVA), followed by Fisher’s Least Significant Difference (LSD) post hoc test for pairwise comparisons. The *p*-value < 0.05 was considered statistically significant.

## Results

### Resveratrol alleviates renal fibrosis in the *db/db* chronic kidney disease mice model

**Fig. 1:**
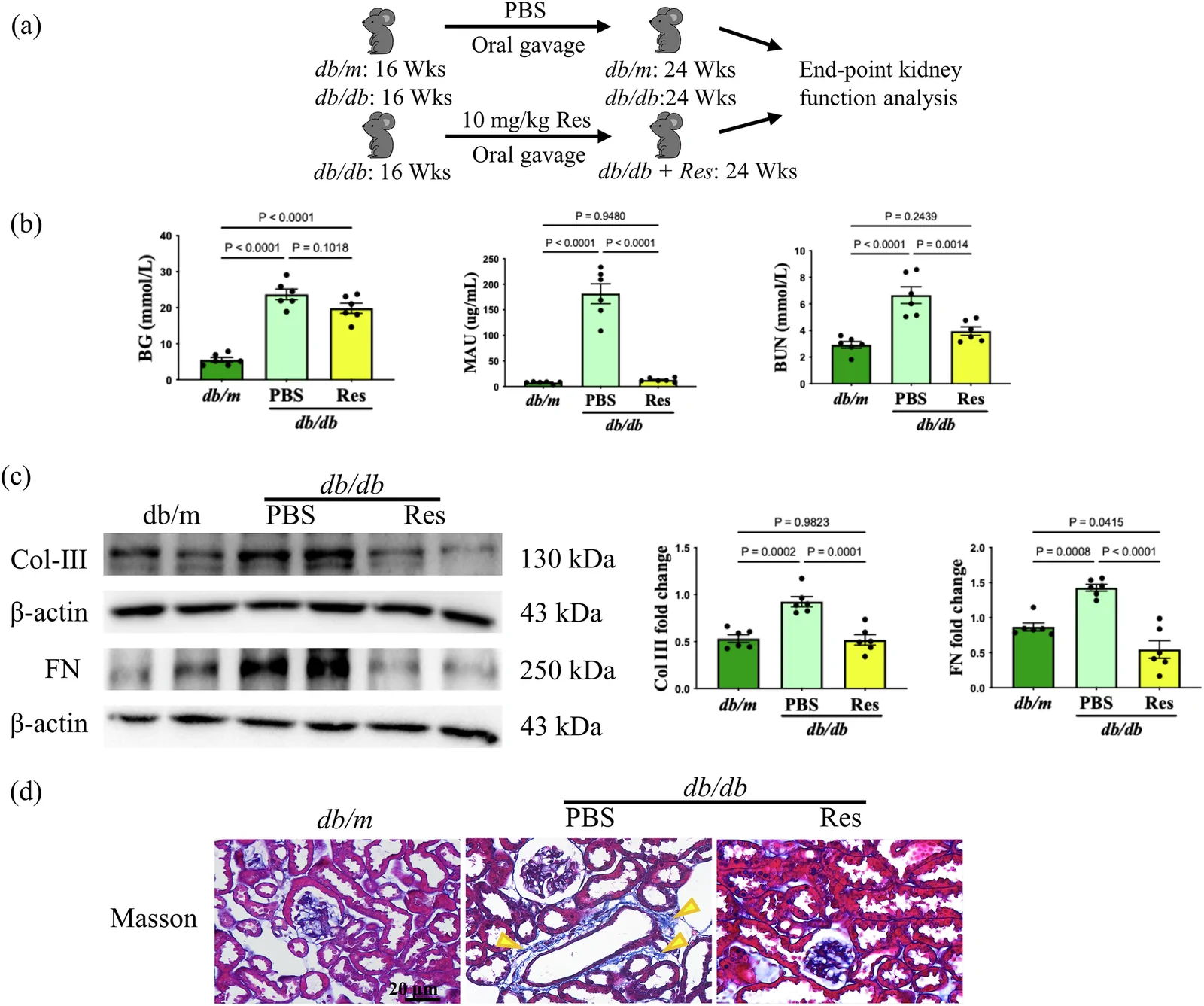
Resveratrol mitigates renal fibrosis in *db/db* chronic kidney disease mice model. **a** Experimental design scheme of resveratrol treatment in *db/db* mice model. **b** Laboratory serum testing of *db/m* and *db/db* mice. BG: blood glucose, BUN: blood urea nitrogen, MAU: microalbuminuria ( = 6). **c** Representative immunoblots and statistical results of renal fibrosis biomarkers, tissue Collagen-III and FN expression in subgroup. FN Fibronectin ( = 6). **d** Representative images of mouse kidney sections staining for Masson trichrome stain. The yellow arrow points to the damaged renal tubule (*n* = 6). Scale bar, 20 μm. All data are represented as mean ± s.d. Data presented in (**b**, **c**) were analyzed via one-way ANOVA with Tukey’s multiple comparisons test. All tests were two-sided. Source data are provided as Supplementary Data 1 file.

To identify the therapy efficiency of attenuating kidney fibrosis by resveratrol in the diabetic kidney disease (DKD) model, *db/db* mouse with diabetes kidney damage was used to treated with resveratrol (10 mg/kg/day) for 8 weeks^13^ (Fig. 1a). Hyperglycemia was confirmed by higher blood glucose (BG) level in the *db/db* mice when compared with age-matched *db/m* mice. Interestingly, there is no significant difference with blood glucose level between the *db/db* mice and the resveratrol-treated mice, indicating that resveratrol has less effect on regulating blood glucose level in the *db/db* mice. Despite the remaining blood glucose level, the total cholesterol (T-CHO), triglycerides (TG), blood urea nitrogen (BUN), and microalbuminuria (MAU) levels were decreased in resveratrol-treated mice compared with the *db/db* mice, suggesting that resveratrol alleviated DKD progression (Fig. 1b and S1a). In addition, the protein expression of fibrosis makers (collagen-III and fibronectin) in kidney tissue was alleviated in the resveratrol-treated mice (Fig. 1c). Masson’s trichrome staining (Fig. 1d) showed that tubular collagen deposition and tubulointerstitial fibrosis were alleviated in resveratrol-treated mice, indicating that resveratrol rescues tubulointerstitial fibrosis development in the *db/db* mice model.

### Resveratrol delays renal tubular cellular senescence via regulating the E2fs family under diabetes condition

**Fig. 2:**
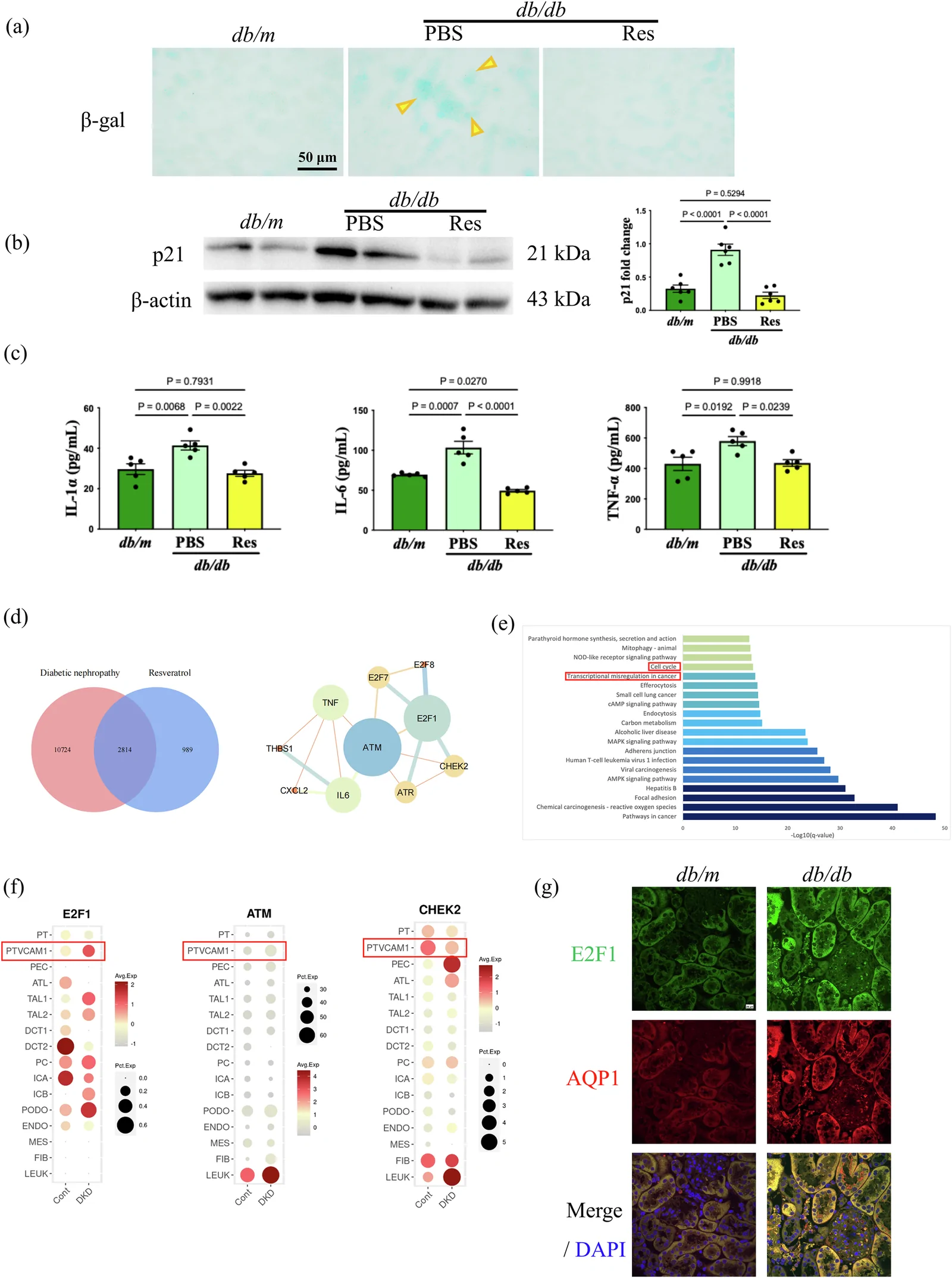
Resveratrol mitigates renal senescence via transcriptional regulation and E2f-mediated DNA damage pathways. **a** Representative micrographs of mouse kidney sections evaluated via senescence-associated β-galactosidase (SA-β-gal) staining (Scale bar, 50 μm) (*n* = 6). The yellow arrow points to the positive senescence tissue. b) Representative immunoblots and corresponding densitometric quantification of p21 protein expression in whole kidney tissue lysates (*n* = 3). **c** Enzyme-linked immunosorbent assay (ELISA) quantification of IL-1α, IL-6, and TNF-α levels in kidney tissues isolated from *db/m* control mice, vehicle-treated *db/db* mice (PBS), and resveratrol-treated *db/db* mice (*n* = 5). **d** Network pharmacology and protein-protein interaction (PPI) mapping. Genes associated with diabetic nephropathy and putative resveratrol targets were curated and cross-referenced utilizing GeneCards, TCMSP, SwissTargetPrediction, CTD, and SEA databases. **e** Kyoto Encyclopedia of Genes and Genomes (KEGG) pathway enrichment analysis of the identified intersection targets, generated utilizing Metascape. **f** Evaluation of target gene expression profiles across renal cell clusters based on single-cell transcriptomic atlas datasets (Humphreys Lab). **g** Representative immunofluorescence co-staining of E2f1 (green) and the proximal tubule marker Aquaporin-1 (AQP1; red) in mouse kidney sections (*n* = 3). All data are represented as mean ± s.d. Data presented in (**b**, **c**) were analyzed via one-way ANOVA with Tukey’s multiple comparisons test. All tests were two-sided. Source data are provided as Supplementary Data 1 file.

Our previous study suggested that tubular senescence is associated with renal hyperglycemic memory in DKD^15^. Targeting senescent cells serve as therapy for renal fibrosis in CKD^16^. Importantly, resveratrol treatment significantly reduced senescence-associated β-galactosidase (SA-β-gal) staining in renal sections compared to the *db/db* mice (Fig. 2a). Consistent with this histological observation, resveratrol attenuated the expression of the senescence marker p21 in kidney tissues (Fig. 2b). Furthermore, ELISA analysis confirmed that resveratrol treatment suppressed the renal levels of senescence-associated secretory phenotype (SASP) factors, specifically IL-1α, IL-6, and TNF-α (Fig. 2c). The above results suggest that resveratrol alleviates renal fibrosis and corresponding cellular senescence in the *db/db* mice model.

To further clarifies the potential mechanism of how resveratrol regulating renal fibrosis and corresponding cellular senescence, network pharmacology analysis for protein-protein-interaction (PPI) prediction was used by overlap related genes of diabetic nephropathy and resveratrol targets genes (Fig. 2d). Genes annotation shows that cell cycle and DNA damage are top enrichment pathways (Fig. 2e). DNA damage genes (ATM and Chk2) and DNA damage transcription factors (E2F1, E2F7 and E2F8) are potential targets of resveratrol in DKD regulation. Interestingly, single cell atlas analysis (https://humphreyslab.com/SingleCell/) indicated that VCAM1 positive tubular epithelial cells^17^ express significantly upregulating E2F1 and ATM expression in human DKD renal tissue (Fig. 2f). Our tissue section staining (Fig. 1d**and S1b**) indicated that tubular injury and fibrosis are mainly targeted by resveratrol. Notably, co-immunostaining of kidney sections from the *db/db* mice revealed robust colocalization of E2f1 with AQP1, an established marker of the proximal tubule and loop of Henle (Fig. 2g), indicating that tubular epithelial cells may involve in the E2f1 regulation process. Considering renal tubular epithelial cells (RTEC) play critical role in cellular senescence of acute or chronic kidney fibrosis^16^, and RTEC showed that increased VCAM1 expression after high-glucose stimulation, we therefore analysis VCAM1 positive RTEC in the following study (Figure S2d). The in vitro protein expression of collagen-III, fibronectin (FN) (Figure S3c), and p21 (Figure S3d) were alleviated by resveratrol treatment under high glucose conditions, which is consistent with the in vivo data (Fig. 1c).

### Resveratrol regulates the E2F7 and E2F8 protein expression via direct interaction

**Fig. 3:**
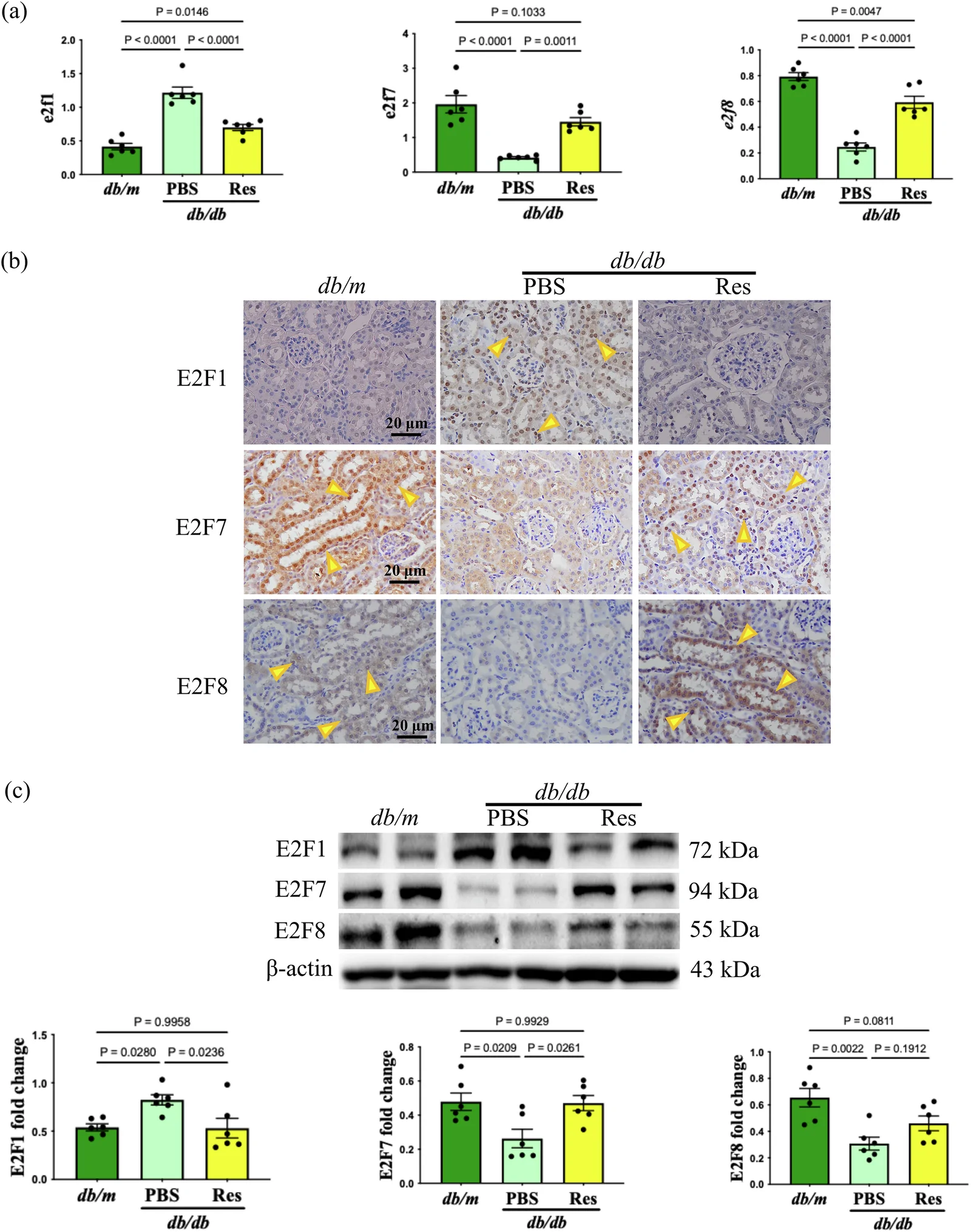
Resveratrol modulates the in vivo expression of E2f transcription factors in mouse renal tissue. **a** Quantitative RT–PCR analysis of *E2f1*, *E2f7*, and *E2f8* mRNA levels in murine kidney tissue (*n* = 3). **b** Representative immunohistochemical images of kidney sections detailing the spatial distribution and subcellular localization of E2f1, E2f7, and E2f8 proteins. (*n* = 6). Scale bar, 20 μm. **c** Representative immunoblots and corresponding densitometric quantification of E2f1, E2f7, and E2f8 protein abundance in whole-kidney lysates (*n* = 3). All data are represented as mean ± s.d. Data presented in (**a**, **c**) were analyzed via one-way ANOVA with Tukey’s multiple comparisons test. All tests were two-sided. Source data are provided as Supplementary Data 1 file.

**Fig. 4:**
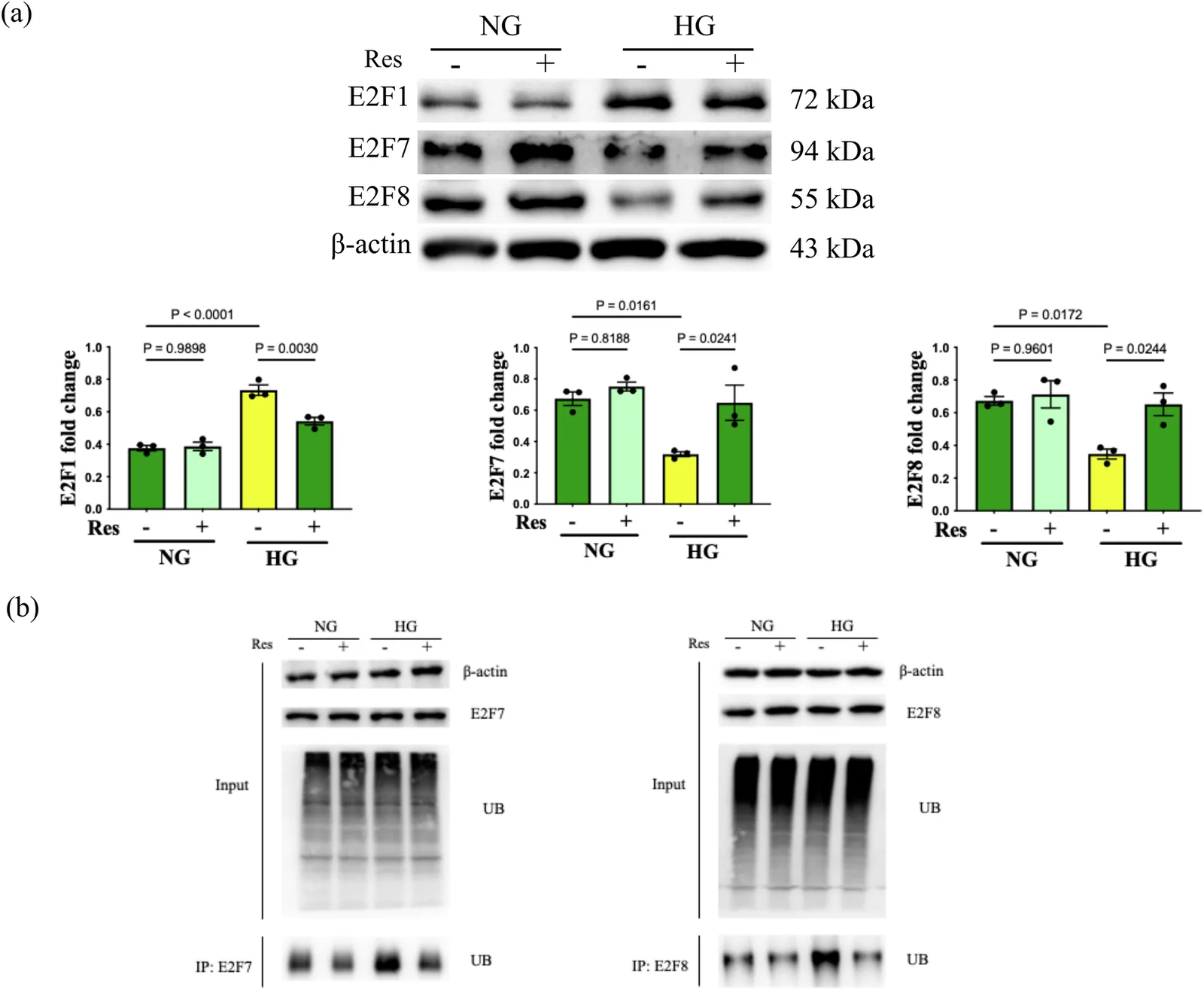
Impact of resveratrol on E2f7 and E2f8 expression and ubiquitination in mouse renal tubular epithelial cells (mRTECs). **a** Representative immunoblots and corresponding densitometric quantification of E2f1, E2f7, and E2f8 protein levels in mRTECs treated with 15 µM resveratrol under high-glucose conditions (*n* = 3). **b** Co-immunoprecipitation (co-IP) analysis demonstrating the interaction between ubiquitin and E2f7 (left) or E2f8 (right). Cell lysates were immunoprecipitated using anti-E2F7 or anti-E2F8 antibodies, followed by immunoblotting with an anti-ubiquitin antibody (*n* = 3). All data are represented as mean ± s.d. Data presented in (**a**) were analyzed via one-way ANOVA with Tukey’s multiple comparisons test. All tests were two-sided. Source data are provided as a Supplementary Data 1 file.

As E2F1 activation is implicated in driving replicative senescence^18^, we investigated the regulation of E2F1 expression by resveratrol. The analysis of renal tissue from the *db/db* mice revealed a significant upregulation of *E2f1* mRNA accompanied by a marked downregulation of both *E2f7* and *E2f8* expression, which could be reversed by resveratrol (Fig. 3a). Previous study suggested that E2F7 and E2F8 exert unique negative feedback on the *E2F* transcriptional network, primarily regulating the E2F1-p53 apoptotic axis^19^. Notably, we observed that E2f7 and E2f8 protein expressions were suppressed under hyperglycemia but were restored by resveratrol treatment (Fig. 3b, c). Furthermore, protein expression results confirmed that resveratrol modifies E2f1, E2f7, and E2f8 levels in vitro, which is consistent with the in vivo data (Fig. 4a). Collectively, these findings demonstrate that resveratrol modulates E2f1, E2f7, E2f8 expression in mRTEC under high glucose stimuli and the *db/db* mice model.

To further understand the underlining mechanism of how resveratrol regulates E2F7 and E2F8 expression, we found that a direct interaction pocket between resveratrol and E2F7/E2F8 via molecular docking (Figure S3a). Furthermore, cellular thermal shift assay (CETSA) results suggested that resveratrol application stable the thermal stability of E2f7 and E2f8 protein (Figure S3b), indicating that resveratrol may interact directly with E2f7 and E2f8 protein for stabilization.

Since the specific contribution of E2F1-mediated DKD senescence and tubular pathology remains to be elucidated. Abnormal ubiquitination is well studied in DKD, for example, E3 ubiquitin ligase of the TRIM subfamily is notably associated with renal fibrosis in DKD^20^. Interestingly, previous study showed that Cyclin F recruits ubiquitin-proteasome complexes to degrade protein E2F7^21^, and E2F8 is a direct target of the APC/C ubiquitin ligase^22^. We therefore hypothesis that high glucose levels affect the E2F7and E2F8 expression via increased ubiquitination. In the mRTEC co-IP experiments (Fig. 4b), we observed that increased ubiquitination of E2f7 and E2f8 proteins in cells treated with high glucose, compared to cells cultured under normal glucose conditions. This suggests that high glucose reduces the E2f7 and E2f8 proteins expression through increased ubiquitination. Interestingly, resveratrol treatment attenuated ubiquitination of both proteins in mRTEC with high glucose treatment, suggested that resveratrol may inhibit E2f7/8 ubiquitination through directly protein interaction. Collectively, these findings demonstrate that resveratrol modulates E2f1, E2f7, E2f8 expression in mRTEC under high-glucose stimuli and the *db/db* mice model.

### E2f1 contributes to the development of RTEC senescence via inducing DNA damage in diabetes mice model

The cyclin-dependent kinase inhibitor p21 is a canonical marker of cellular senescence associated with the DNA damage response (DDR). We observed that high-glucose conditions significantly upregulated p21 expression, whereas treatment with resveratrol attenuated this effect both in vitro and in vivo (Fig. 2b and Figure S3d). These findings indicate that hyperglycemia drives cellular senescence, a phenotype amenable to resveratrol-mediated mitigation.

**Fig. 5:**
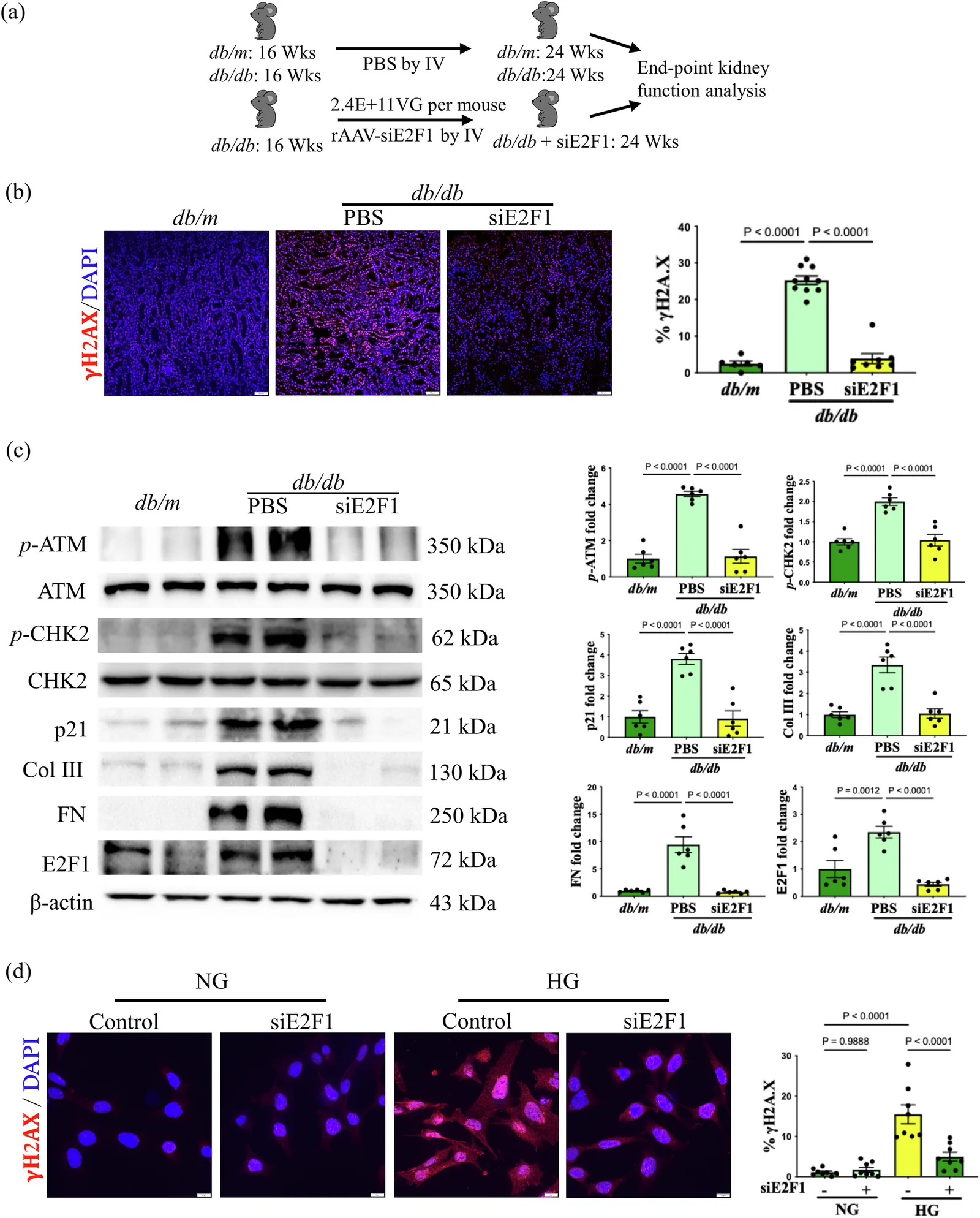
E2f1 promotes renal tubular epithelial cells senescence via DNA damage induction in-vivo and in-vitro. **a** Schematic representation of the experimental design for rAAV-sh*E2f1* administration in *db/db* mice (VG, viral genomes). **b** Representative immunofluorescence images and quantification of γH2AX foci (red) in mouse kidney sections. Scale bar, 50 μm (*n* = 6). **c** Immunoblot analysis and corresponding densitometric quantification of the DNA damage response proteins ATM, CHK2, p21, and fibronectin (FN) in renal tissue lysates (*n* = 3). **d** Representative immunofluorescence images and quantification of γH2AX foci (red) in cultured mRTECs. Scale bar, 10 μm (*n* = 3). All data are represented as mean ± s.d. Data presented in (**b**, **c**) were analyzed via one-way ANOVA with Dunnett’s multiple comparisons test. Data presented in (**d**) were analyzed via one-way ANOVA with Tukey’s multiple comparisons test. All tests were two-sided. Source data are provided as a Supplementary Data 1 file.

To elucidate the role of E2F1 in hyperglycemia-induced renal senescence, we generated an AAV-mediated knockdown mouse model (AAV-sh*E2f1*) (Fig. 5a). Recombinant AAV vectors encoding *E2f1*-targeting shRNA were administered to 16-week-old mice, with peak expression achieved 8 weeks post-injection. Serum biochemical characterization indicated that E2f1 silencing significantly alleviated serum BUN and MAU expression in vivo^9^. Immunofluorescence analysis revealed extensive DNA damage markers in high-glucose-treated mRTEC cells (Fig. 5d) and kidneys from obese mice (Fig. 5b). Notably, *E2f1* silencing significantly alleviated DNA damage. Furthermore, immunoblotting indicated that E2f1 depletion suppresses the ATM/CHK2 signaling axis (Fig. 5c). Interestingly, combined ChIP-seq analysis demonstrates E2F1 regulation of the ATM-CHK2 signaling axis via direct promoter binding (Figure S5). E2F1 directly targets the promoter region of *ATM* and *CHK2*. Collectively, these data demonstrate that E2F1 promotes DNA damage via activation of the ATM/CHK2 pathway.

Moreover, pathways enrichment of the resveratrol-gene interaction suggested that the pathway of Recruitment and ATM-mediated phosphorylation of repair and signaling proteins at DNA double strand breaks (REACT:R-HSA-5693565) were significantly enriched with 35 annotated genes (Supplementary Table 3, Figure S4a). Both ATM and CHK2 were included in the annotated genes (Figure S4b), indicating that E2F1 may be the downstream regulation of resveratrol.

### Both E2f7 and E2f8 reduces renal tubular epithelial cells senescence and fibrosis via downregulating E2F1 expression

**Fig. 6:**
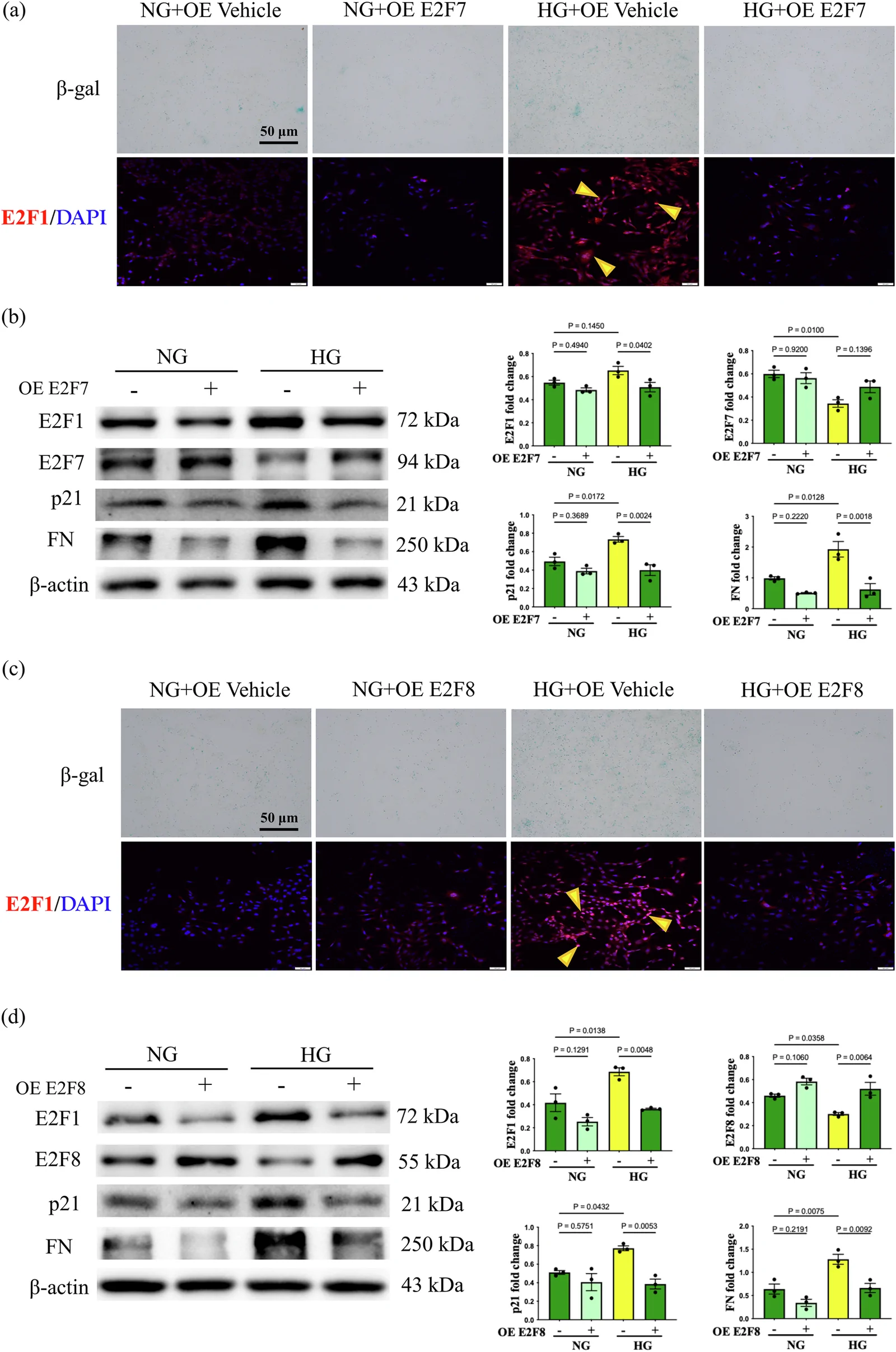
E2f7 and E2f8 attenuate tubular cells senescence and fibrosis via regulating E2f1 expression. **a** Representative images of mRTECs showing SA-β-gal staining (Scale bar, 50 μm) and E2f1 immunostaining (red. Scale bar, 20 μm) upon E2f7 overexpression (*n* = 3). The yellow arrow points to the positive-stained nuclear. b) Representative immunoblots and quantification of E2f1, E2f7, p21, and fibronectin (FN) expression in mRTECs upon E2f7 overexpression (*n* = 3). **c** Representative images of mRTECs showing SA-β-gal staining (Scale bar, 50 μm) and E2f1 immunostaining (red. Scale bar, 20 μm) upon E2f8 overexpression (*n* = 3). **d** Representative immunoblots and quantification of E2f1, E2f8, p21, and FN expression in mRTECs upon E2f8 overexpression (*n* = 3). Scale bar, 50 μm. All data are represented as mean ± s.d. Data presented in (**b**, **d**) were analyzed via one-way ANOVA with Tukey’s multiple comparisons test. All tests were two-sided. Source data are provided as a Supplementary Data 1file.

E2F7 inhibits cell cycle arrest and senescence by coupling the p53 and Rb pathways to repress cell cycle genes, including E2F1. To determine whether the transcriptional repressor E2F8 shares similar capability, we examined the effects of overexpression E2f7 and E2f8 in mRTECs cultured under high-glucose conditions. E2f7 overexpression mitigated cellular senescence and fibrosis while downregulating E2f1 expression (Fig. 6a-b, S6b). E2f8 overexpression recapitulates this phenotype (Fig. 6c, d, S6b). More specifically, elevated E2f8 levels repressed E2f1 protein expression, reduced the population of SA-β-gal positive cells, and downregulated both p21 and FN expression.

Notably, ectopic expressions of either E2f7 or E2f8 effectively rescued mRETCs from high-glucose-induced S-phase arrest (Fig. S6a) and concomitantly attenuated activation of the ATM/CHK2 signaling axis (Figure S6c). Collectively, these findings demonstrate that E2f7 and E2f8 suppress senescence and fibrotic remodeling in mRTECs via the targeted downregulation of E2f1 expression.

### E2f7 and E2f8 synergistically repress *E2f1* transcription to mitigate cellular senescence and fibrosis

**Fig. 7:**
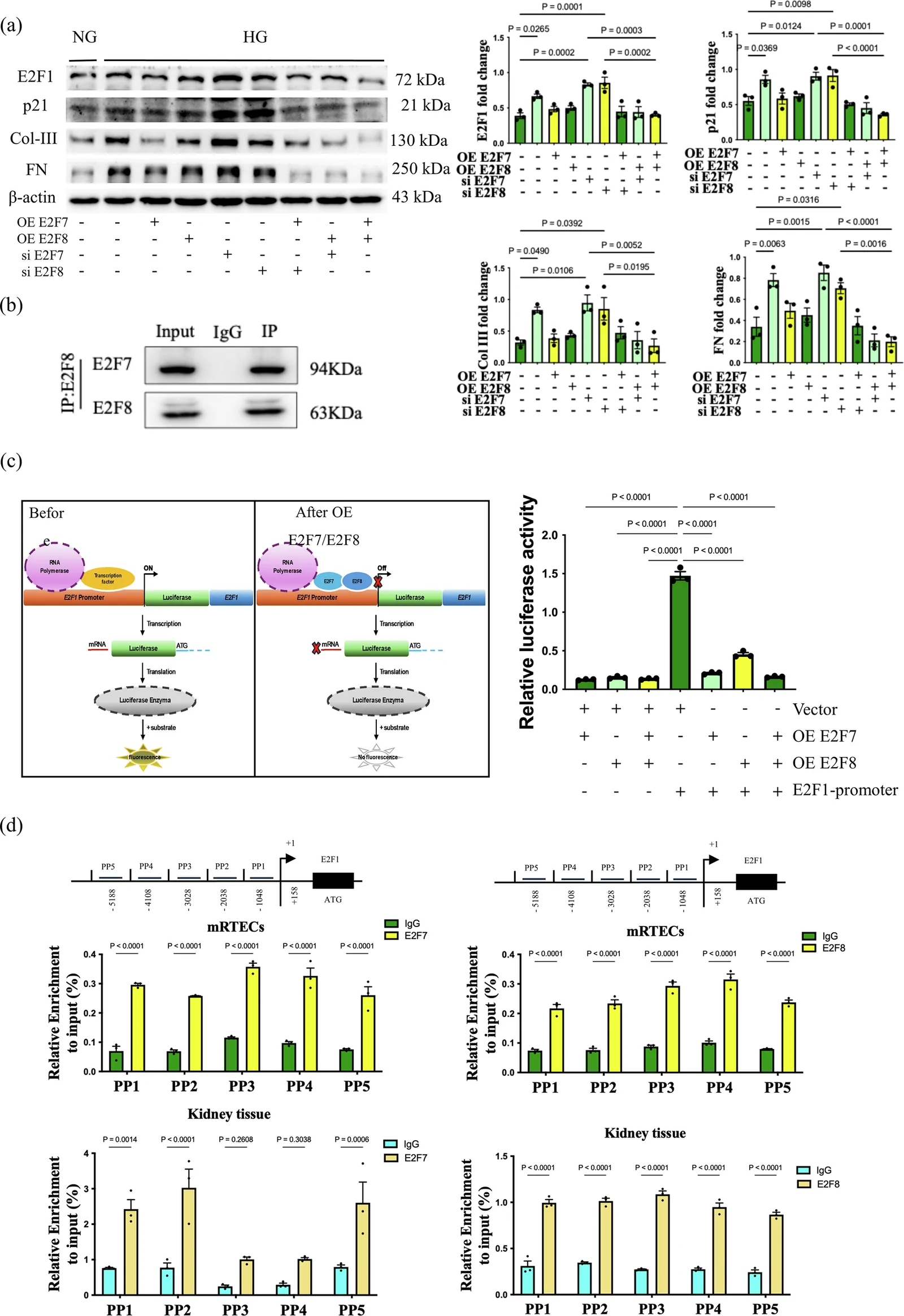
Synergistic repression of E2f1 expression by E2f7 and E2f8. **a** Representative immunoblots and densitometric quantification of E2f1, p21, Col-III, and FN protein levels in mRTECs (*n* = 3). **b** Co-immunoprecipitation (Co-IP) assay demonstrating the interaction between E2f7 and E2f8. Lysates were immunoprecipitated with an anti-E2F8 antibody followed by immunoblotting for E2f7 and E2f8. Input represents the loading control (*n* = 3). **c** Schematic representation of reporter constructs (left) and dual-luciferase reporter activity (right). mRTECs cultured in high glucose (30 mM) were co-transfected with E2f7 and/or E2f8 overexpression plasmids and an *E2F1* promoter-luciferase reporter (or empty vector) for 6 h (*n* = 3). **d** ChIP-qPCR analysis of mRTECs and db/m kidney tissue for E2f7 and E2f8 occupancy at the *E2f1* promoter. Enrichment was quantified using five primer pairs (PP1-PP5) spanning the promoter region. IgG and H3K4me3 served as negative and positive controls, respectively (*n* = 3). All data are represented as mean ± s.d. Data presented in (**a**, **c**) were analyzed via one-way ANOVA with Tukey’s multiple comparisons test. Data presented in (**d**) were analyzed via two-way ANOVA with Šídák’s multiple comparisons test. All tests were two-sided. Source data are provided as a Supplementary Data 1 file.

The synergistic function of E2F8 with E2F7 is important for embryonic development^19^, cancer treatment^23^. Homo- and heterodimers of E2F7 and E2F8 were found on target E2f1 promoter^24^. To elucidate the cooperative function of E2f7 and E2f8, we assessed the protein levels of E2f1 and downstream senescence- and fibrosis-associated markers under high glucose conditions. While the individual overexpression of E2f7 or E2f8 (with reciprocal knockdown) attenuated E2f1 levels and reduced fibrosis, the co-expression of both factors resulted in a more potent suppression of E2f1, p21, FN, and collagen-III (Fig. 7a). Immunoblotting confirmed that the combined impact of E2f7 and E2f8 exceeds their individual effects. Mechanistically, co-immunoprecipitation (Co-IP) assays revealed that E2f7 and E2f8 physically interact to form heterodimers (Fig. 7b). Furthermore, dual-luciferase reporter (DLR) assays demonstrated that while E2f7 or E2f8 alone inhibit *E2f1* promoter activity, their combined action is synergistic (Fig. 7c). To map the binding interface, we designed primer pairs spanning the open chromatin domain of the *E2f1* promoter. Subsequent analysis indicated that E2f7 and E2f8 occupy multiple sites within this promoter region to blockade E2f1 production (Fig. 7d, e), both in mRTECs lysates and kidney tissue lysates. Collectively, these data indicate that E2f7 and E2f8 heterodimerize and bind the *E2f1* promoter to repress expression, thereby alleviating high glucose-induced senescence and fibrosis in mRTECs.

### Possible correlation of E2F1 with human clinical DKD samples

To determine the clinical relevance of E2F1 in human DKD, we interrogated the Nephroseq (https://nephroseq.org/) transcriptomic database. We observed a significant upregulation of *E2F1* mRNA in the tubulointerstitium of DKD patients compared to healthy controls (Fig. S7a). Notably, tubulointerstitial E2F1 expression positively correlated with the glomerular filtration rate (GFR) within the DKD cohort (Fig. S7b)^8,25^. To validate these findings at the protein level, we performed immunohistochemical analysis on human renal biopsies. Consistent with our transcriptomic profiling, sections from a DKD patient demonstrated elevated E2F1 protein expression and a concurrent reduction in E2F7 levels relative to an age-matched health control (Figure S7c), suggesting that the regulation of E2F1 in RETCs is involved in DKD development.

## Discussion

**Fig. 8:**
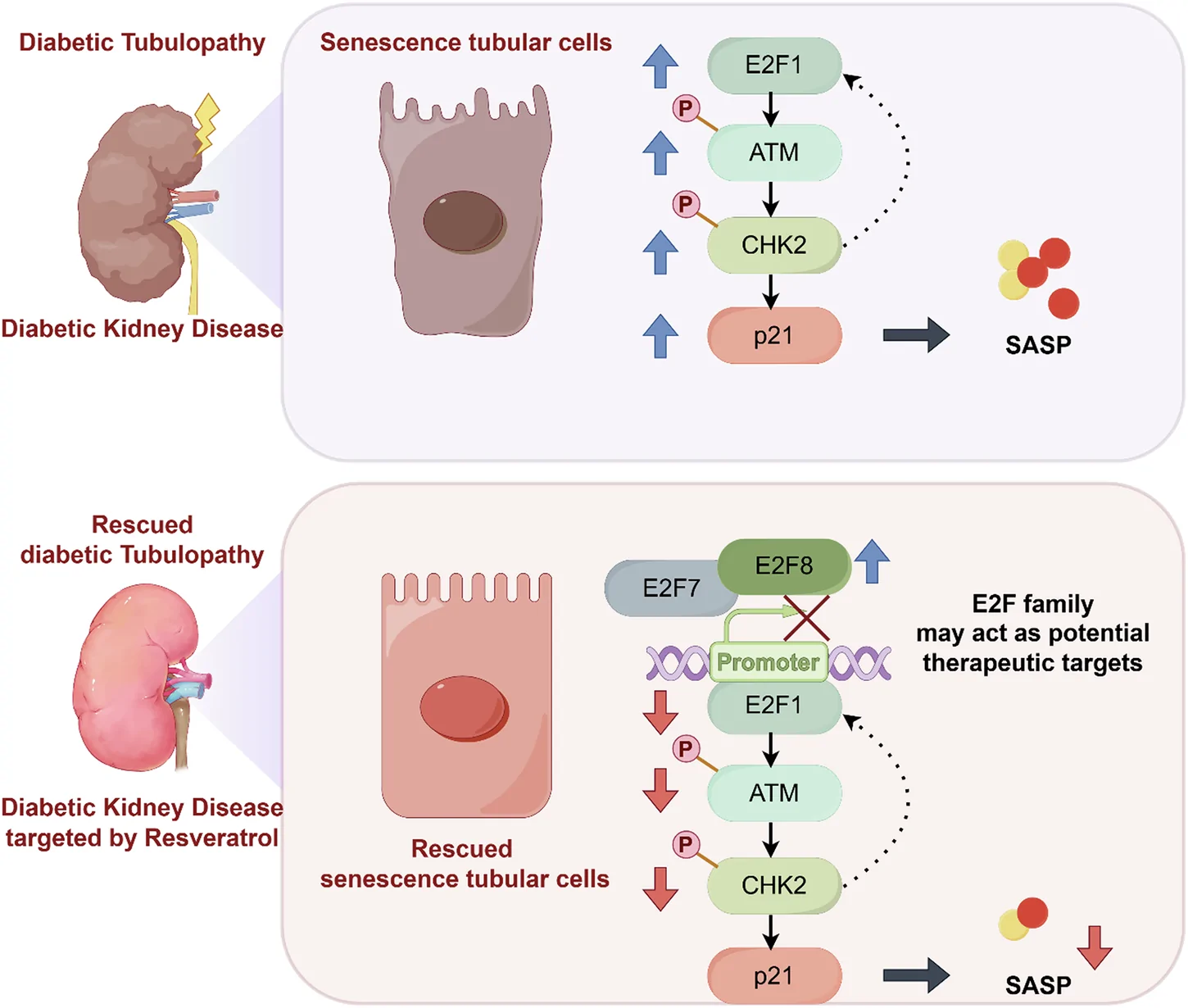
Proposed mechanism of E2f1-mediated tubular senescence and transcriptional rescue by E2f7/8 heterodimers. In diabetic tubular cells, E2f1 upregulation triggers DNA damage via the ATM/CHK2 signaling axis, leading to p21-dependent senescence. Consequently, senescent tubular cells release SASP factors, exerting detrimental paracrine effects on neighboring cells. The atypical family members E2f7 and E2f8 function as transcriptional repressors that restrain E2f1 expression. Resveratrol administration in the *db/db* mice modulates the E2f7/8-E2f1 axis, thereby attenuating tubular senescence in DKD.

Diabetic kidney disease (DKD) is a leading cause of end-stage renal disease, characterized pathologically by glomerular basement membrane thickening, glomerular hypertrophy, and tubulointerstitial fibrosis associated with extracellular matrix (ECM) deposition^26^. Here, we investigate the regulatory function of the E2F transcription factor family in DKD-associated renal tubular epithelial cell (RTEC) senescence and fibrosis. Using high-glucose in vitro systems and the *db/db* diabetic mouse models, we demonstrate that the transcriptional activator E2f1 is significantly upregulated in DKD. This upregulation promotes RTEC senescence by inducing DNA damage via the ATM/CHK2 signaling axis. We further show that the transcriptional repressors E2f7 and E2f8 synergistically inhibit *E2f1* transcription to mitigate cellular senescence and fibrosis. Furthermore, we identify E2F7/E2F8-mediated repression of E2F1 as the mechanism underlying the anti-fibrotic effects of resveratrol (Fig. 8). Collectively, these findings define the E2F signaling network as a possible, therapeutically targetable driver of DKD progression.

Previous study suggested that E2F regulatory loops controlling the development of cancer cell and polyploidization in mammalian cells^27–29^. Our current study map the E2F7/8-E2F1 axis within the context of metabolic stress in DKD progression. Our results suggested that there was a causal link between hyperglycemia-induced E2F1 dysregulation and the senescence of RTECs. The E2F7/8-E2F1 interaction also prevents the expression of downstream senescence-associated target genes. By demonstrating that modulating this axis alleviates DNA damage and renal fibrosis in vivo, we expand the biological relevance of the E2F family into metabolic kidney disease. More importantly, we identify resveratrol as a specific upstream modulator of this transcriptional axis in vivo. While resveratrol is known to exert renoprotective effects, the molecular bridge connecting it to cell cycle regulators in DKD remains a critical gap. By proving that resveratrol ameliorates DKD specifically by upregulating E2F7/8 to neutralize E2F1, we may provide a promising mechanistic rationale for targeting this pathway therapeutically.

Resveratrol exhibits diverse pharmacological properties, including anti-fibrotic and anti-senescence activities^30–32^. Previous studies demonstrate that resveratrol normalizes renal metrics (kidney weight, albuminuria, BUN, and serum creatinine) and attenuates fibrosis in streptozotocin (STZ)-induced models by promoting autophagy^33^. Furthermore, resveratrol improves aging-associated metabolic phenotypes in diet-induced obese mice by inhibiting phosphodiesterase (PDE) activity, cAMP levels, and ameliorates renal inflammation via the Sirt1/SphK1 axis^34^. However, the mechanisms by which resveratrol modulates cellular senescence remain incompletely understood. Here, we identify the atypical E2F7 and E2F8, as therapeutic targets of resveratrol in DKD via bioinformatics analysis. Using mouse RTEC cultured under high-glucose conditions, we demonstrate that E2f7 and E2f8 overexpression suppresses E2f1, thereby alleviating DNA damage, cellular senescence, and renal fibrosis. Consistently, oral administration of resveratrol to the *db/db* mice upregulated E2f7/8 and downregulated E2F1, correlating with reduced senescence and fibrosis markers in vivo. Collectively, our findings clarify that resveratrol mitigates high glucose-induced tubular cell senescence and DKD-associated fibrosis through an E2f7/8-dependent repression of E2f1 signaling.

Cellular senescence is a critical driver of dysfunction and tubulointerstitial fibrosis in DKD^35^. Senescent RTEC exacerbate both acute and chronic kidney pathologies via the senescence-associated secretory phenotype (SASP)^36^. Our results also suggested that SASP factors showing the paracrine effect from senescent mRTECs on normal mRTECs (Figure S6d), supporting the evidence for the role of SASP in DKD renal tubular injury. While previous studies establish that cisplatin-induced chronic senescence leads to maladaptive repair and fibrosis, and that targeting tubular senescence mitigates rhabdomyolysis-induced injury^37^, the specific mechanisms driving senescence in DKD remain complex. Hyperglycemia directly induces RTEC senescence, thereby inhibiting mitosis and disrupting mitochondrial function^38^. Furthermore, KIM-1-mediated uptake of palmitic acid-bound albumin has been linked to DNA damage, fibrosis, and secondary glomerulosclerosis^35^. Here, we demonstrate that E2f1 drives DNA damage-induced senescence in hyperglycemic tubular cells. We show that AAV-mediated *E2f1* silencing in vivo significantly mitigates this pathology. Mechanistically, *E2f1* depletion suppresses the ATM/CHK2 signaling axis, concomitant with p21 downregulation. Collectively, our findings suggest that E2f1 promotes mRTEC senescence in DKD by activating the ATM/CHK2-mediated DNA damage pathway.

The E2F family of transcription factors orchestrates cell cycle progression and is broadly categorized into transcriptional activators (E2F1-E2F3) and transcriptional repressors, the latter comprising canonical (E2F4-E2F6) and atypical (E2F7-E2F8) subsets^7^. Among these, E2F1 is pivotal for DNA replication and cell cycle entry. However, E2F1 dysregulation is implicated in pathological states. E2F1 promotes proliferation in CKD yet paradoxically regulates mitotic fidelity and the SASP to inhibit cell cycle progression under senescent conditions. While E2F1 is known to induce mesenchymal stem cell senescence via the Cyclin D1/CDK4/E2F1 axis^29^, its role in high glucose-induced senescence remains undefined. In contrast, the atypical repressors E2F7 and E2F8 constitute a unique regulatory arm that modulates the E2F1-p53 apoptotic axis^39^. Evidence suggests E2F7 couples the p53 and Rb pathways to prevent aberrant cell cycle arrest and directly represses *E2F1* transcription^40^. Similarly, E2F8 functions as a transcriptional repressor, and the simultaneous deletion of E2F7 and E2F8 enhances tumor angiogenesis in various malignancies^23,41,42^.

Here, we investigate the E2f7/E2f8-E2f1 regulatory axis in DKD, specifically within mRTEC. We demonstrate that high glucose exposure upregulates E2f1, triggering DNA damage-dependent senescence. Silencing *E2f1* via shRNA in both the *db/db* mice and mRTECs significantly attenuated the DNA damage phenotype and downregulated downstream effector gene expression. Mechanistically, we found that E2f7 and E2f8 directly repress *E2f1* transcription. Chromatin immunoprecipitation assays revealed that E2F7 binds to the accessible chromatin domain of the *E2f1* promoter^27,43^. Furthermore, our data indicates that E2f7 and E2f8 act synergistically likely via heterodimerization to potently inhibit E2f1 expression, thereby mitigating E2f1-driven senescence in mRTEC under hyperglycemic conditions.

Several limitations of this study warrant consideration. Notably, tubulointerstitial E2F1 expression exhibited an unexpected positive correlation with GFR within the DKD cohort. We posit that this divergence from our in vivo findings reflects temporal disparities in disease progression. More specifically, animal models likely recapitulate early-stage pathogenesis, whereas the clinical cohort predominantly represents advanced disease. Future clinical investigations are required to systematically delineate the relationship between E2F expression dynamics, the severity of renal tubular injury, and the histological stage of interstitial fibrosis. Second, the precise molecular intermediaries coupling E2F1 upregulation to ATM/CHK2 activation still require further characterization, though we identified that E2F1 could directly bind to ATM/CHK2 promoter via public ChIP dataset. Third, the distinct spatiotemporal role of E2F1 in podocytes remains largely unknown. While single cell atlas analysis revealed that E2F1 expression is also significantly upregulated in podocytes from patients with DKD, its direct contribution to massive tubulointerstitial fibrosis is lacking investigation. In the present study, we mainly focus on defining the E2F7/8-E2F1 axis within RTECs. Further research warrants for studying the functional role of podocyte-derived E2F1 in the pathogenesis of kidney fibrosis with DKD. Finally, validating the translational relevance of this signaling axis in DKD necessitates corroboration within longitudinal clinical cohorts.

In conclusion, we demonstrate that the transcriptional activator E2F1 is upregulated in DKD, driving RTEC senescence via the ATM/CHK2 DNA damage response axis. The transcriptional repressors E2f7 and E2f8 synergistically inhibit E2f1 transcription to attenuate cellular senescence and fibrosis. Crucially, we identify this E2f7/E2f8-mediated repression of E2f1 as the mechanistic basis for the anti-fibrotic efficacy of resveratrol. Collectively, these findings provide the E2F signaling network as a possible therapeutic target for mitigating tubular senescence in diabetic kidney disease.

### Resource availability

#### Materials availability

This study did not generate new or unique reagents.

## Supplementary information

Transparent Peer Review file

Supplementary Information

Description of Additional Supplementary Files

Supplementary Data 1

## Data Availability

Public data used in this study are available in the GeneCards (Genes associated with diabetic nephropathy sources: https://www.genecards.org/). The resveratrol target gene and E2F1 target gene sources are available in: https://old.tcmsp-e.com/ ; http://swisstargetprediction.ch/ ; http://ctdbase.org ; http://sea.bkslab.org/; https://cytoscape.org/^44^; https://string-db.org/^45^. The original contributions presented in the study are included in the Supplementary Data 1, including the quantitative source data and the unmodified original blot images. Source data are shared in Figshare (https://figshare.com/) with DOI: 10.6084/m9.figshare.34452318. Any additional information required to reanalyze the data reported in this paper is available from the lead contact.

## Code availability

This paper does not generate or report any original code.
