# Supplementary material for: E2F7 and E2F8 attenuate diabetic kidney disease by suppressing E2F1-induced tubular senescence
Source: Commun Biol. 2026 Nov 13;9:1526. doi: 10.1038/s42003-026-11334-1 (PMC13768344; doi:10.1038/s42003-026-11334-1)
Supplement: Supplementary file 1 [file 42003_2026_11334_MOESM1_ESM.pdf]

nature portfolio

Peer Review File

E2F7 and E2F8 attenuate diabetic kidney disease by suppressing E2F1 induced tubular senescence

Corresponding Author: Dr Shihai Jiang

This file contains all reviewer reports in order by version, followed by all author rebuttals in order by version.

Version 0:

Reviewer comments:

Reviewer #1

(Remarks to the Author)
This manuscript mainly demonstrated that the transcriptional activator E2F1 was significantly upregulated in DKD, driving RTEC senescence via the ATM/CHK2 DNA damage response axis. The transcriptional repressors E2F7 and E2F8 synergistically inhibited E2F1 transcription to attenuate cellular senescence and fibrosis. Furthermore, this study identified that the anti-fibrotic efficacy of resveratrol was mediated by the E2F7/E2F8-dependent repression of E2F1. Overall, the results of this study are interesting, but not unexpected. And several important concerns need to be carefully addressed.

Major concerns
1.Given that previous studies have reported that E2F7 and E2F8 can inhibit the transcription of E2F1, this poses a significant challenge to the innovation of this study. Therefore, the authors need to clearly articulate the innovation of this research.
2.In Fig. S1a, single cell atlas analysis revealed that E2F1 expression is significantly upregulated in podocytes from patients with diabetic kidney disease. It remains unclear whether podocyte-derived E2F1 contributes to the pathogenesis of kidney fibrosis. The expression pattern and functional role of E2F1 in podocytes would be also interesting.
3.The manuscript demonstrated the interaction between resveratrol and E2F7/E2F8 only by molecular docking in Fig. S1c. The conclusion that resveratrol directly binds to these transcription factors would be substantially strengthened by additional biochemical validation, such as surface plasmon resonance (SPR) or cellular thermal shift assay (CETSA).
4. The underlying mechanism by which resveratrol modulates the ATM/CHK2 signaling axis is not explained in the manuscript. The authors should clarify whether this regulation is direct or indirect and provide supporting data.
5. The functional and mechanistic studies of E2F7 and E2F8 in Figs. 6 and 7 were restricted to in vitro cellular experiments. It is necessary to use appropriate animal models to confirm the physiological relevance.
6. There is a lack of information on the mechanism by which high glucose levels affect the E2F7and E2F8 expression.
7. The conclusions of this study were drawn from animal models and cell lines, with no validation in clinical samples from patients with DKD.

Minor concerns
1. The tubular localization of E2F7, E2F8, and E2F1 remains unclear. I would suggest including co-localization studies using confocal microscopy with segment-specific markers (e.g., for proximal tubules, distal tubules, or collecting ducts).
2. There were no obvious changes in the staining of Masson in Fig. 1d and Sirius Red in Fig. S1d of the db/db mice with resveratrol treatment.
3. In Fig. 6a and 6b, the P values in the statistical graphs were covered and not clearly visible.
4. In Fig. 2a, the anti-senescence effect of resveratrol is only supported by SA-β-gal staining. I would suggest including additional senescence markers (e.g., p21 and γH2AX) to provide more comprehensive evidence for the anti-senescent effect of resveratrol.
5. The authors propose that SASP factors released by E2F1-induced senescent cells contribute to renal fibrosis in the manuscript and Fig. 7. However, the expression of SASP factors was not examined. It is suggested that the authors detect the expression of typical SASP factors using qPCR or ELISA.
6. The supplemental figures are not presented in a logical order and the Fig. S1e, as referenced in the text, is missing from the supplemental materials. Please reorganize all supplemental panels in sequential order and ensure that all referenced figures are included.
7. In Fig 5b and d, there were no obvious changes in the expression of E2F7 and E2F8 in mRTEC with E2F7

overexpression or E2F8 overexpression. Please explain that.
8. The manuscript only used the db/db mouse model to investigate DKD pathogenesis and therapeutic effects. However, validating the key findings in a second model, such as STZ-induced diabetic mice, would significantly strengthen the generalizability of the conclusions.

Reviewer #2

(Remarks to the Author)
In this manuscript, Li et.al. investigate the E2F1-E2F7/8 regulatory axis and its pivotal role in hyperglycemia-induced tubular cellular senescence. The study reveals that elevated E2F1 expression in diabetic nephropathy (DN) models activates DNA damage response (DDR) pathways, subsequently driving cellular senescence. The authors demonstrate that the atypical E2F family members, E2F7 and E2F8, repress E2F1 transcription and translation by binding to and masking the E2F1 promoter. Additionally, they identify resveratrol as a pharmacological modulator of this E2F axis.
Overall, the study offers valuable mechanistic insights into diabetic kidney disease (DKD) through a coherent experimental design. The findings carry significant translational potential. However, several major points regarding phenotypic validation, mechanistic directness, and in vivo physiological relevance require further clarification and experimental support before acceptance for publication:

1. Quantitative validation of cell cycle arrest is required. Cell cycle arrest is a definitive hallmark of cellular senescence. While the authors have provided marker-based evidence, the manuscript would be significantly strengthened by direct quantification of cell cycle distribution: Incorporate flow cytometric analysis (e.g., PI staining or BrdU/EdU incorporation) to rigorously confirm the transition to a senescence-associated growth arrest in the tubular cell models.
2. The authors establish that E2F1 knockdown reduces phosphorylation within the ATM/CHK2 pathway and that E2F7/8 overexpression suppresses E2F1. However, the connection between E2F7/8 upregulation and the attenuation of the DDR remains associated: Authors should provide direct evidence demonstrating that overexpressing E2F7 and E2F8 significantly decreases the phosphorylation levels of ATM and CHK2. This is essential to confirm that the E2F7/8-E2F1 axis is the primary driver of the observed DDR modulation and bridging the Mechanistic Gap between E2F7/8 and the ATM/CHK2 Axis.
3. The study concludes that E2F7/8 suppresses E2F1 by binding to its promoter region. To solidify this claim of transcriptional regulation, authors should provide E2F1 mRNA expression data (via RT-qPCR) following E2F7/8 overexpression. This will clarify whether the observed reduction in E2F1 protein levels is a direct consequence of transcriptional repression or involves secondary post-translational mechanisms.
4. Physiological Characterization of the in vivo Model
The use of db/db mice with E2F1 knockdown is central to this paper’s logical framework. However, the manuscript lacks an assessment of whether this genetic intervention actually preserves renal function. Authors should provide data on renal functional parameters (e.g., urinary albumin-to-creatinine ratio, serum creatinine) and histological analysis (e.g., PAS staining for basement membrane thickening or fibrosis quantification) comparing E2F1-knockdown db/db mice to their db/m counterparts.

The study presents a compelling mechanism for tubular senescence in DKD. I recommend that Editors consider this manuscript for publication provided the authors address these concerns with additional experimental data and refined analysis.

Reviewer #3

(Remarks to the Author)
This study focuses on the regulatory mechanism of renal tubular epithelial cell senescence in diabetic kidney disease (DKD). It demonstrates that E2F1 promotes senescence by inducing DNA damage, while E2F7 and E2F8 exert a protective effect through transcriptional inhibition of E2F1. Furthermore, it reveals that resveratrol may exert an anti - fibrotic effect by regulating this axis. The overall study design is reasonable, with diverse experimental approaches and abundant data, holding significant scientific significance and potential clinical application value. However, the study could be further strengthened with more in-depth mechanistic dissection, stricter experimental rigor, and improved data presentation. Specific comments are as follows:
1. The authors demonstrate that E2F7/8 bind to the E2F1 promoter and inhibit its transcription via ChIP-qPCR and dual-luciferase reporter assays. Nevertheless, the current data are solely derived from in vitro overexpression systems, and the binding of E2F7/8 to the E2F1 promoter at the chromatin level has not been verified in db/db mouse renal tissues. It is recommended to perform ChIP-qPCR experiments in renal tissues to enhance the in vivo relevance of this regulatory mechanism.
2. The discussion mentions that senescent cells exacerbate renal injury through senescence-associated secretory phenotype (SASP) factors, but the study did not detect the expression and secretion of SASP-related factors, such as IL-6, TNF-α, MMP-9. It is suggested to supplement the detection of mRNA and protein levels of key SASP factors, and verify the paracrine effect of the supernatant from senescent mRTECs on normal mRTECs through conditioned medium experiments, so as to strengthen the evidence for the role of SASP in renal tubular injury.
3. The study only verified the mechanism in mouse models and cell lines, lacking correlation validation with human DKD samples. It is recommended to supplement immunohistochemical/immunofluorescence analyses of renal biopsy tissues from DKD patients and normal controls, detect the expression levels of E2F1/E2F7/E2F8, and analyze their correlation with the degree of renal tubular injury and the stage of interstitial fibrosis.
4. The study states that E2F1 knockdown can reduce the phosphorylation level of ATM/CHK2, but it remains unclear

whether E2F1 directly binds to the promoters of these genes. It is suggested to supplement ChIP experiments for verification, or analyze E2F1-regulated DNA damage-related genes through transcriptome sequencing to clarify its mechanism of action.
5. The study used 30 mmol/L high glucose to treat mRTECs. It is recommended that the authors supplement the rationale for the selection of the 30 mmol/L high glucose concentration and treatment duration, which can be supported by citing relevant literature or providing pre-experimental data (time/concentration-dependent changes in cell viability and senescence marker expression) to enhance the scientific rigor of the experimental design.

Version 1:

Reviewer comments:

Reviewer #1

(Remarks to the Author)
Basically, the authors have answered questions.

Reviewer #3

(Remarks to the Author)
My concerns have been addressed. No more comments.

Open Access This Peer Review File is licensed under a Creative Commons Attribution 4.0 International License, which permits use, sharing, adaptation, distribution and reproduction in any medium or format, as long as you give appropriate credit to the original author(s) and the source, provide a link to the Creative Commons license, and indicate if changes were made.
In cases where reviewers are anonymous, credit should be given to 'Anonymous Referee' and the source.
The images or other third party material in this Peer Review File are included in the article’s Creative Commons license, unless indicated otherwise in a credit line to the material. If material is not included in the article’s Creative Commons license and your intended use is not permitted by statutory regulation or exceeds the permitted use, you will need to obtain permission directly from the copyright holder.
To view a copy of this license, visit https://creativecommons.org/licenses/by/4.0/

Point-by-point response to reviewers

We would like to thank the anonymous reviewers for their constructive suggestions and comments with the manuscript.

Reviewer 1:

This manuscript mainly demonstrated that the transcriptional activator E2F1 was significantly upregulated in DKD, driving RTEC senescence via the ATM/CHK2 DNA damage response axis. The transcriptional repressors E2F7 and E2F8 synergistically inhibited E2F1 transcription to attenuate cellular senescence and fibrosis. Furthermore, this study identified that the anti-fibrotic efficacy of resveratrol was mediated by the E2F7/E2F8-dependent repression of E2F1. Overall, the results of this study are interesting, but not unexpected. And several important concerns need to be carefully addressed.

Response: We thank the reviewer for finding our study of interest and pointing out several important concerns that certainly improve the manuscript, please find our response to the concerns as follows.

1. Given that previous studies have reported that E2F7 and E2F8 can inhibit the transcription of E2F1, this poses a significant challenge to the innovation of this study. Therefore, the authors need to clearly articulate the innovation of this research.

Response: We thank the reviewer for raising this critical point regarding the conceptual advance of our work. We agree that classical models characterize the atypical E2Fs (E2F7 and E2F8) as transcriptional repressors of the E2F1 gene, primarily through DNA-binding competition at the E2F1 promoter (**EMBO Rep. 2008 Mar;9(3):252-9.**). However, our study fundamentally advances the field by defining a previously unrecognized pathophysiological context.
While E2F regulatory loops are well-documented in oncology and cancer cell cycle biology (e.g. U2OS cells in *LP Zalmas. et.al.* EMBO Rep. 2008), from our best knowledge, our study is the first to map the E2F7/8-E2F1 axis within the context of metabolic stress and diabetic kidney disease (DKD). We establish a causal link between hyperglycemia-induced E2F1

dysregulation and the senescence of renal tubular epithelial cells (RTECs). The E2F7/8-E2F1 interaction also prevents the expression of downstream senescence-associated target genes. By demonstrating that modulating this axis alleviates DNA damage and renal fibrosis in *db/db* mice, we expand the biological relevance of the E2F family into metabolic kidney disease. More importantly, we identify resveratrol as a specific upstream modulator of this exact transcriptional axis in vivo. While resveratrol is known to exert renoprotective effects, the molecular bridge connecting it to cell cycle regulators in DKD remains a critical gap. By proving that resveratrol ameliorates DKD specifically by upregulating E2F7/8 to neutralize E2F1, we provide a promising mechanistic rationale for targeting this pathway therapeutically.

We revised the following section to highlight the innovation of this research at Line 434-446, Page 18:

> Previous study suggested that E2F regulatory loops controlling the development of cancer cell and polyploidization in mammalian cells. From our best knowledge, our study may be the first to map the E2F7/8-E2F1 axis within the context of metabolic stress in DKD progression. Our results suggested that there was a causal link between hyperglycemia-induced E2F1 dysregulation and the senescence of RTECs. The E2F7/8-E2F1 interaction also prevents the expression of downstream senescence-associated target genes. By demonstrating that modulating this axis alleviates DNA damage and renal fibrosis *in vivo*, we expand the biological relevance of the E2F family into metabolic kidney disease. More importantly, we identify resveratrol as a specific upstream modulator of this exact transcriptional axis *in vivo*. While resveratrol is known to exert renoprotective effects, the molecular bridge connecting it to cell cycle regulators in DKD remains a critical gap. By proving that resveratrol ameliorates DKD specifically by upregulating E2F7/8 to neutralize E2F1, we provide a promising mechanistic rationale for targeting this pathway therapeutically.

2. In Fig. S1a, single cell atlas analysis revealed that E2F1 expression is significantly upregulated in podocytes from patients with diabetic kidney disease. It remains unclear whether podocyte-derived E2F1 contributes to the pathogenesis of kidney fibrosis. The expression pattern and functional role of E2F1 in podocytes would be also interesting.

Response: We appreciate the reviewer's insightful observation regarding the upregulation of

E2F1 in podocytes within the single-cell atlas analysis. We strongly agree that podocyte injury represents a critical initiating event in DKD (**Nat Rev Endocrinol. 2026 Jan;22(1):21-35**) and that the functional role of podocyte-derived E2F1 warrants thorough investigation.

While we acknowledge the significance of podocytes in diabetes glomerular pathology, the primary focus of the current manuscript is fundamentally centered on tubulointerstitial fibrosis driven by cellular senescence in RTECs. We prioritized RTECs for several reasons. First, the tubulointerstitium constitutes most of the renal parenchyma, and the severity of tubulointerstitial fibrosis correlates more closely with renal function decline than glomerular alterations. Second, our *in vivo* and *in vitro* data demonstrate that injured RTECs are one of the primary sources of the SASP in DKD model, which directly dictates the pro-fibrotic microenvironment. Third, while podocyte-derived E2F1 or podocyte-enriched E2F7/8-E2F1 axis contributes to glomerular sclerosis in DKD remains highly interest, its direct contribution to massive tubulointerstitial fibrosis is likely indirect. We assume that podocyte-derived E2F1 acting secondary to glomerular filtration barrier failure and subsequent proteinuria-induced tubular toxicity.

Fully dissecting the distinct spatiotemporal role of E2F1 in podocytes requires the generation and extensive characterization of podocyte-specific (e.g., *Nphs2*-Cre) *E2f1* knockout and overexpression murine models. Given the extensive scope required to rigorously establish this parallel mechanism, in the present study, we mainly first focus on defining the E2F7/8-E2F1 axis within RTECs. Further research warrants for studying the functional role of podocyte-derived E2F1 in the pathogenesis of kidney fibrosis.

To address the reviewer's valid point, we have revised the manuscript to discuss the E2F1 expression pattern in podocytes in the discussion section at Line 511-517, Page 21:

> Third, the distinct spatiotemporal role of E2F1 in podocytes remains largely unknown. While single cell atlas analysis revealed that E2F1 expression is also significantly upregulated in podocytes from patients with DKD, its direct contribution to massive tubulointerstitial fibrosis is lacking investigation. In the present study, we mainly focus on defining the E2F7/8-E2F1 axis within RTECs. Further research warrants for studying the functional role of podocyte-derived E2F1 in the pathogenesis of kidney fibrosis with DKD.

3. The manuscript demonstrated the interaction between resveratrol and E2F7/E2F8 only by molecular docking in Fig. S1c. The conclusion that resveratrol directly binds to these

transcription factors would be substantially strengthened by additional biochemical validation, such as surface plasmon resonance (SPR) or cellular thermal shift assay (CETSA).

Response: We fully agree on additional biochemical validation on resveratrol and E2F7/E2F8 directly interacting would strengthen the reliability of the results. Accordingly, the CETSA results were shown in **(Fig. S3b)**. Compared to vehicle (DMSO) group, resveratrol increases both E2F7 and E2F8 protein stability from 37 to 62 °C, which indicated by the slowing of protein degradation. This result validated the predictions from the molecular docking analysis.

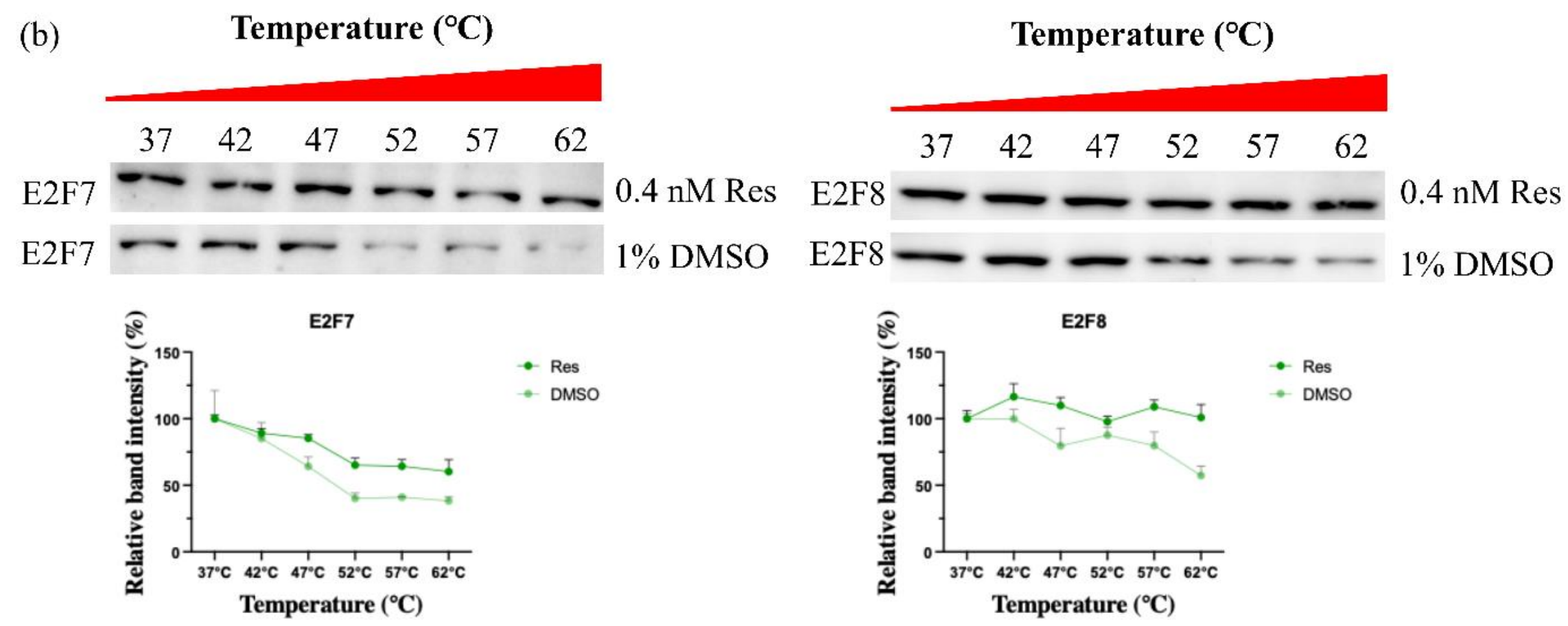

Accordingly, we have revised the manuscript at <u>Line 326-329, Page 14:</u>

Furthermore, cellular thermal shift assay (CETSA) results suggested that resveratrol application stable the thermal stability of E2F7 and E2F8 protein (Fig. S3b), indicating that resveratrol may interact directly with E2F7 and E2F8 protein for stabilization.

4. The underlying mechanism by which resveratrol modulates the ATM/CHK2 signaling axis is not explained in the manuscript. The authors should clarify whether this regulation is direct or indirect and provide supporting data.

Response: We thank the reviewer for this critical feedback. We agree that defining the precise hierarchical link between resveratrol and the ATM/CHK2 axis is essential for completing the mechanistic model. We have performed additional experiments to address this point, which indicated that resveratrol could modulates the ATM/CHK2 axis both directly and indirectly. We first utilized Comparative Toxicogenomics Database (https://ctdbase.org/, NIH) to understand what the Resveratrol-Gene interaction could be. Pathways were enriched

significantly among genes that interact with resveratrol and show only terms with a corrected *p*-value <0.01. The pathway Recruitment and ATM-mediated phosphorylation of repair and signaling proteins at DNA double strand breaks (REACT:R-HSA-5693565) were shown significantly enriched with 35 annotated genes (**Table S1**). More importantly, both ATM and CHK2 were included in the annotated genes (**Fig. S4a, b**, https://cytoscape.org/). These results suggested that resveratrol could modulate the ATM/CHK2 axis directly.

Table. S1 The 35 annotated genes interacted with resveratrol. The pathway Recruitment and ATM-mediated phosphorylation of repair and signaling proteins at DNA double strand breaks (REACT:R-HSA-5693565) analyzed by CTD.

ABL1	CHEK2	H2BC17	KAT5	RNF8
APBB1	EYA1	H2BC21	MAPK8	SMARCA5
ATM	EYA2	H2BC5	MRE11	SUMO1
BAP1	EYA3	H2BC6	NBN	TP53
BARD1	EYA4	H2BC8	NSD2	TP53BP1
BRCA1	H2AX	H4C13	RAD50	UBB
BRCC3	H2BC15	H4C6	RNF168	UBC

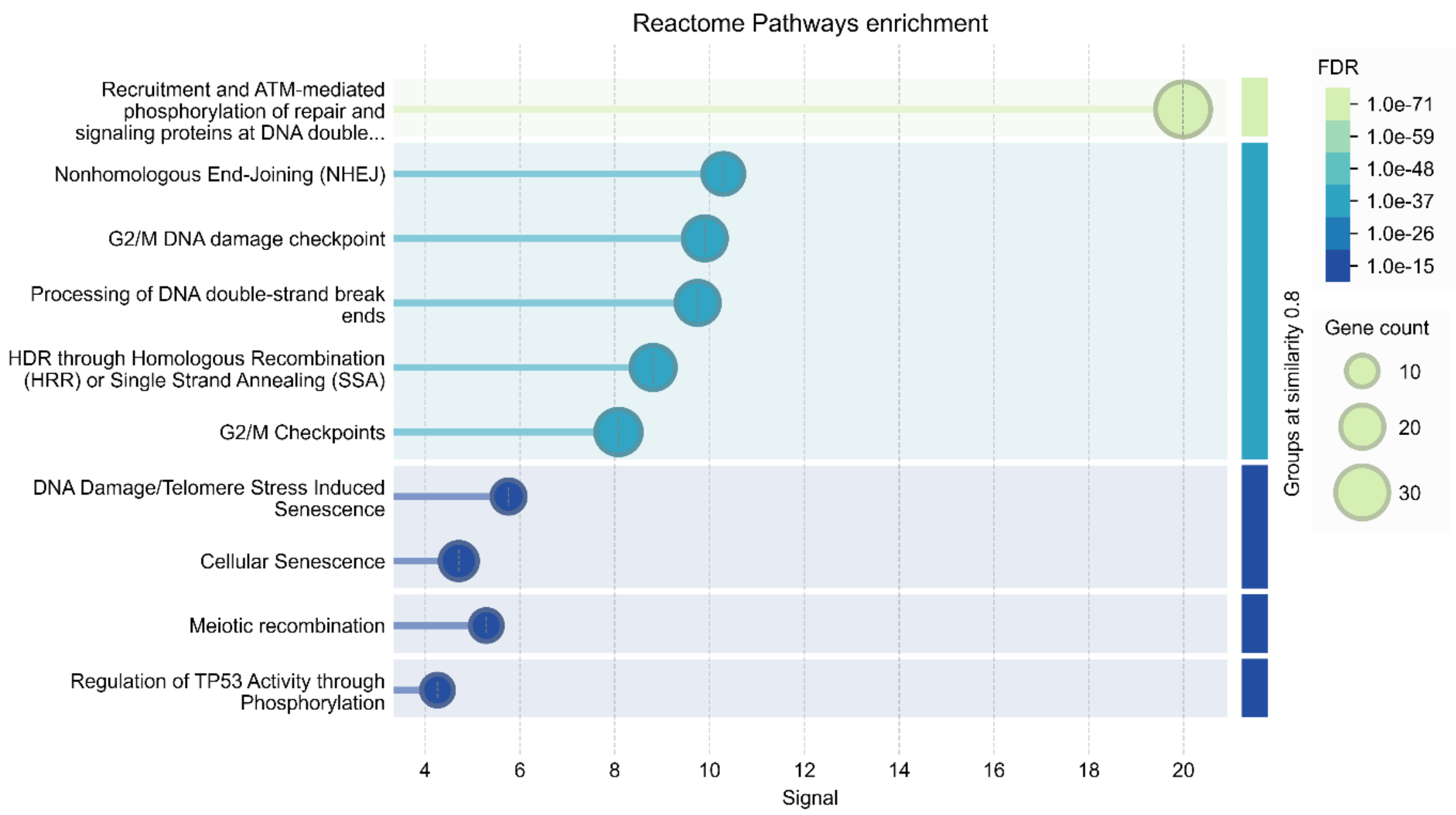

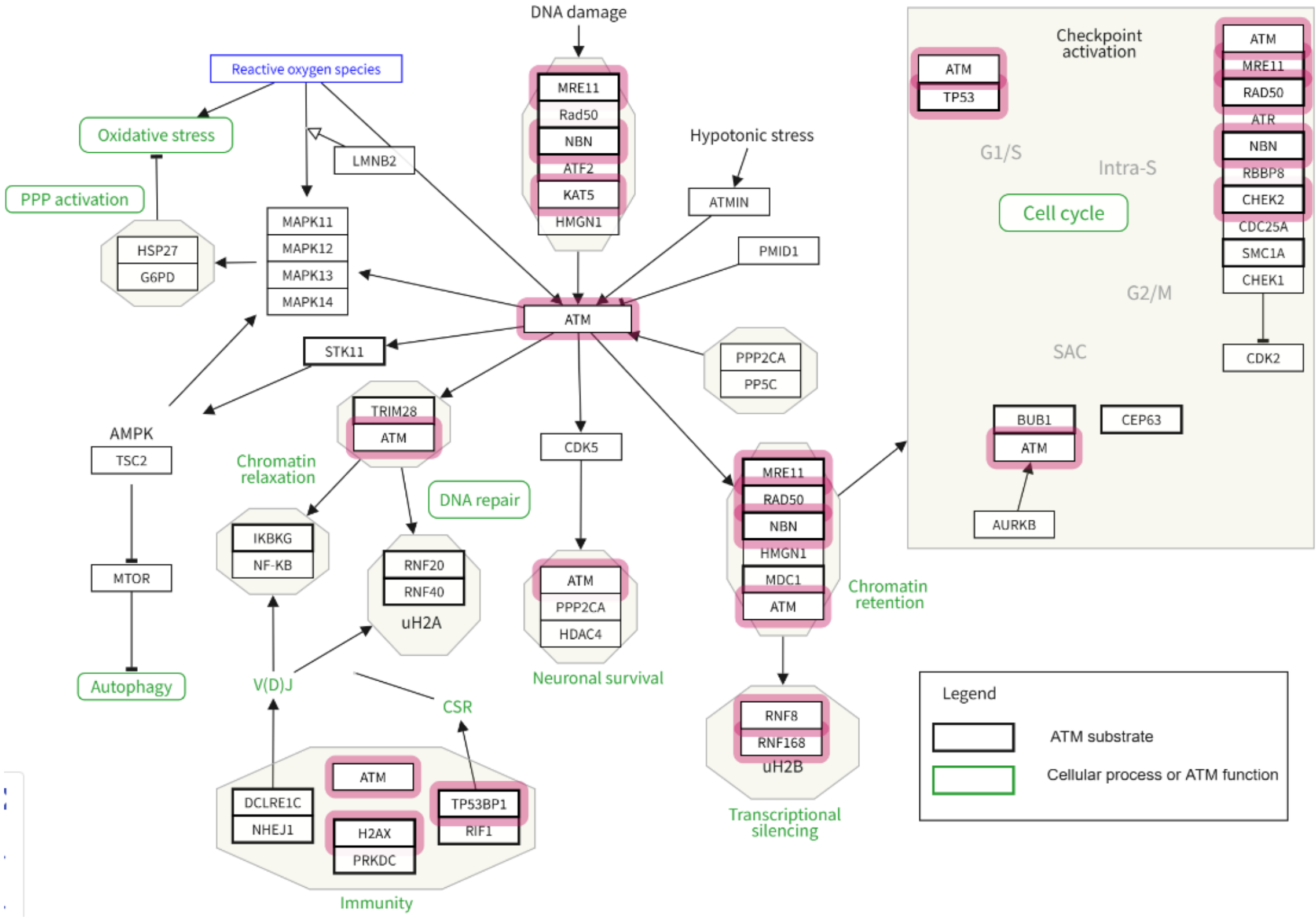

In addition, our presenting results indicated that resveratrol regulates E2F7/8-E2F1 axis within RTECs. As we validated that resveratrol directly interacting with E2F7/E2F8 (Response point 3), and E2F1 directly targets the promoter region of ATM and CHK2 (**Fig. S5**), all the above results therefore suggested that resveratrol could also modulate the ATM/CHK2 axis indirectly, particularly via E2F7/8-E2F1-ATM axis within RTECs.

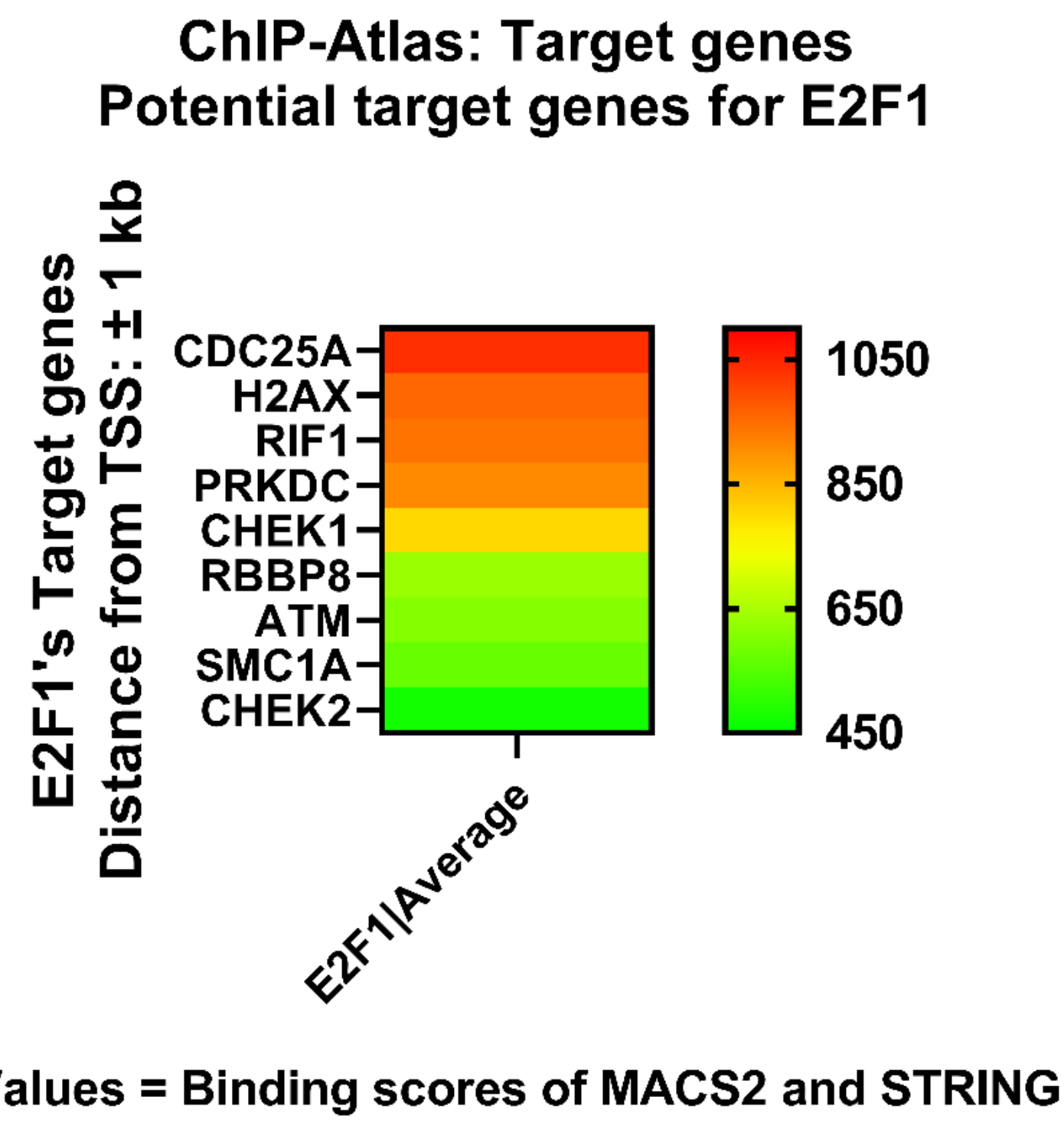

To address the reviewer's comment, we have revised the manuscript to discuss the interaction between resveratrol and the ATM/CHK2 axis at Line 366-371, Page 16:

Interestingly, pathways enrichment of the resveratrol-gene interaction suggested that the pathway of Recruitment and ATM-mediated phosphorylation of repair and signaling proteins at DNA double strand breaks (REACT:R-HSA-5693565) were significantly enriched with 35 annotated genes (Supplementary Table 3, Fig. S4a). Both ATM and CHK2 were included in the annotated genes (Fig. S4b), indicating that E2F1 may be the downstream regulation of resveratrol.

5. The functional and mechanistic studies of E2F7 and E2F8 in Figs. 6 and 7 were restricted to in vitro cellular experiments. It is necessary to use appropriate animal models to confirm the physiological relevance.

Response: We thank the reviewer for this valuable suggestion. In revised experiments, we therefore further validated the functions and mechanisms of E2F7 and E2F8 in animal models. We performed CHIP-qPCR analysis on kidney tissue from normal C57/BL mice, and the results were consistent with those from the *in vitro* experiments: both E2F7 and E2F8 bind directly to the promoter region of E2F1, indicating that E2F7/8-E2F1 regulatory mechanism is verified in renal tissues (**Fig. 7d**).

(d)

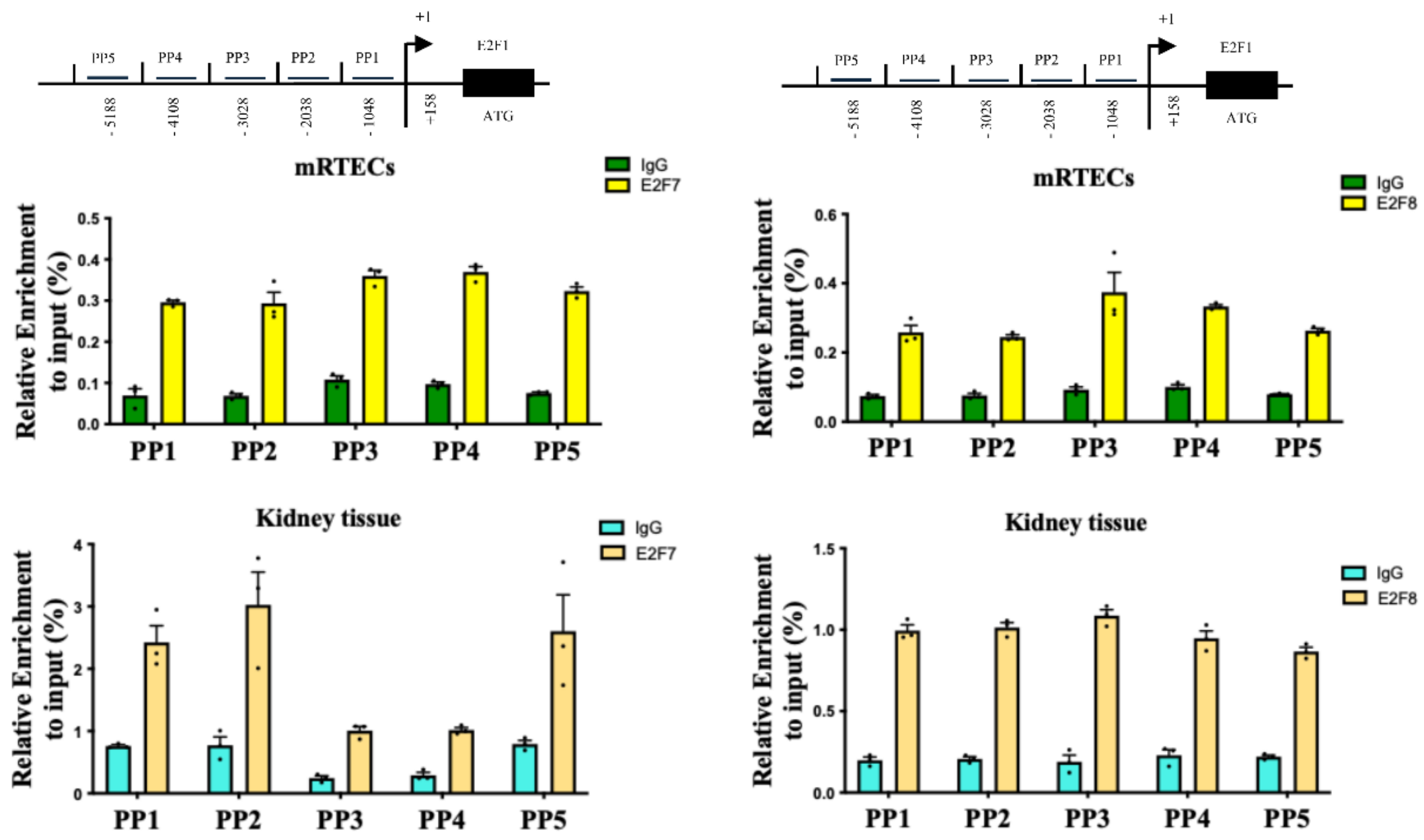

The corresponding text in the Results section has been updated to reflect these findings (Page

17, Lines 403–405):
Subsequent analysis indicated that E2F7 and E2F8 occupy multiple sites within this promoter region to blockade E2F1 production (Fig. 7d-e), both in mRTECs lysates and kidney tissue lysates. Collectively, these data indicate that E2F7 and E2F8 heterodimerize and bind the E2f1 promoter to repress expression, thereby alleviating HG-induced senescence and fibrosis in mRTECs.

6. There is a lack of information on the mechanism by which high glucose levels affect the E2F7and E2F8 expression.

Response: Abnormal ubiquitination is well studied in DKD, for example, E3 ubiquitin ligase of the TRIM subfamily is notably associated with renal fibrosis in DKD (**Redox Rep. 2024 Dec;29(1):2404794.**). Interestingly, previous study showed that Cyclin F recruits ubiquitin-proteasome complexes to degrade protein E2F7 and E2F8 is a direct target of the APC/C ubiquitin ligase. We therefore hypothesis that high glucose levels affect the E2F7and E2F8 expression via increased ubiquitination. In our RTEC co-IP experiments (**Fig. 4b**), we observed that increased ubiquitination of E2F7 and E2F8 proteins in cells treated with high glucose, compared to cells cultured under normal glucose conditions. This suggests that high glucose reduces the E2F7 and E2F8 proteins expression through increased ubiquitination. Interestingly, resveratrol treatment attenuated ubiquitination of both proteins in RTEC with high glucose treatment, suggested that resveratrol may inhibit E2F7/8 ubiquitination through protein interaction.

(b)

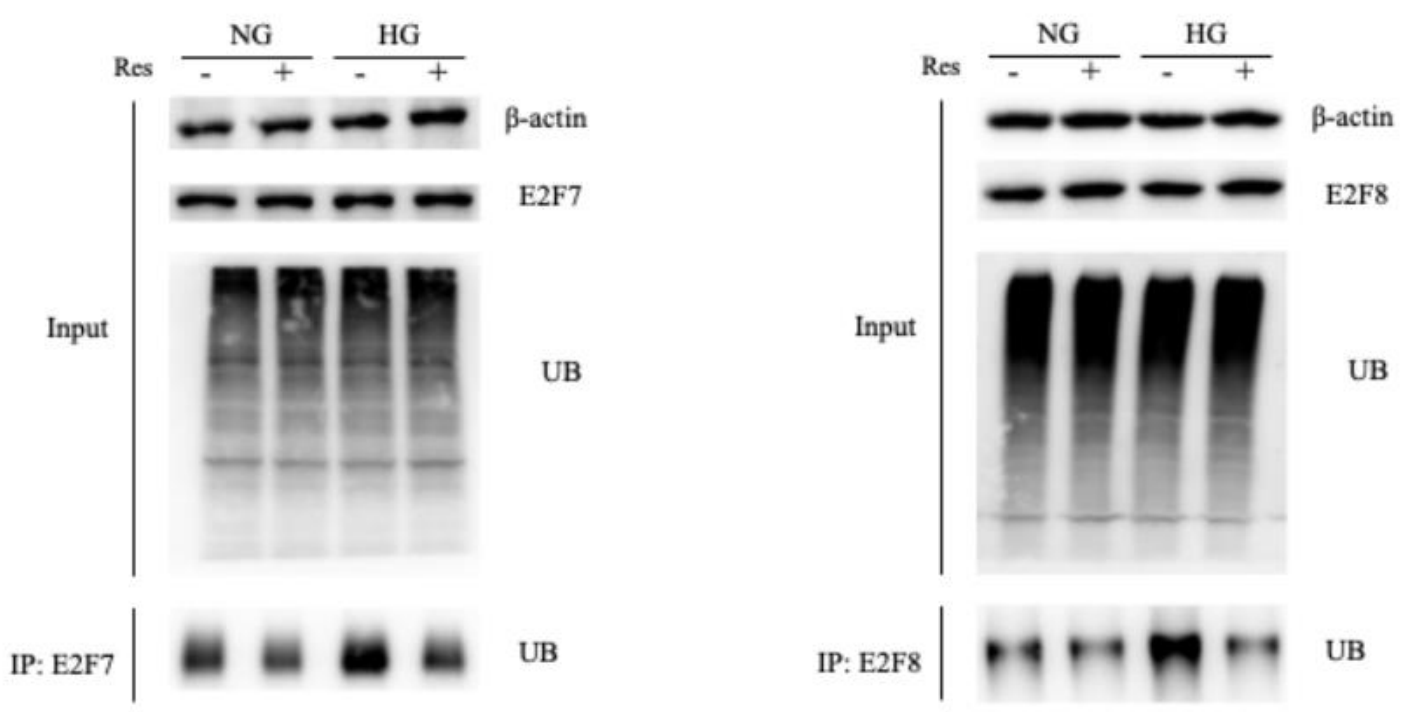

Accordingly, we have revised the manuscript at Line 330-339, Page 14:
Since the specific contribution of E2F1-mediated DKD senescence and tubular pathology remains to be elucidated. Abnormal ubiquitination is well studied in DKD, for example, E3 ubiquitin ligase of the TRIM subfamily is notably associated with renal fibrosis in DKD. Interestingly, previous study showed that Cyclin F recruits ubiquitin-proteasome complexes to

degrade protein E2F7 and E2F8 is a direct target of the APC/C ubiquitin ligase. We therefore hypothesis that high glucose levels affect the E2F7and E2F8 expression via increased ubiquitination. In our mRTEC co-IP experiments (Fig. 4b), we observed that increased ubiquitination of E2F7 and E2F8 proteins in cells treated with high glucose, compared to cells cultured under normal glucose conditions. This suggests that high glucose reduces the E2F7 and E2F8 proteins expression through increased ubiquitination.

7. The conclusions of this study were drawn from animal models and cell lines, with no validation in clinical samples from patients with DKD.

Response: We thank the reviewer for raising this critical point. We agree that validating our mechanistic findings in human clinical samples is essential for establishing the translational relevance of the E2F7/8-E2F1 axis in DKD. To address this limitation in the revised manuscript, we have incorporated both ex vivo analysis of human renal biopsies and orthogonal bioinformatic validation using patient transcriptomic datasets.
To determine the clinical relevance of E2F1 in human DKD, we interrogated the Nephroseq (https://nephroseq.org/) transcriptomic database. We observed a significant upregulation of E2F1 mRNA in the tubulointerstitium of DKD patients compared to healthy controls (**Fig. S7a**). Notably, tubulointerstitial E2F1 expression positively correlated with the glomerular filtration rate (GFR) within the DKD cohort (**Fig. S7b**). To validate these findings at the protein level, we performed immunohistochemical analysis on human renal biopsies. Consistent with our transcriptomic profiling, sections from a DKD patient demonstrated elevated E2F1 protein expression and a concurrent reduction in E2F7 levels relative to an age-matched health control (**Fig. S7c**), suggesting that the regulation of E2F1 in RETCs is involved in DKD development. However, we cannot perform correlation analyses comparing the E2F staining with clinical pathology scores due to limit case in our own cohort.

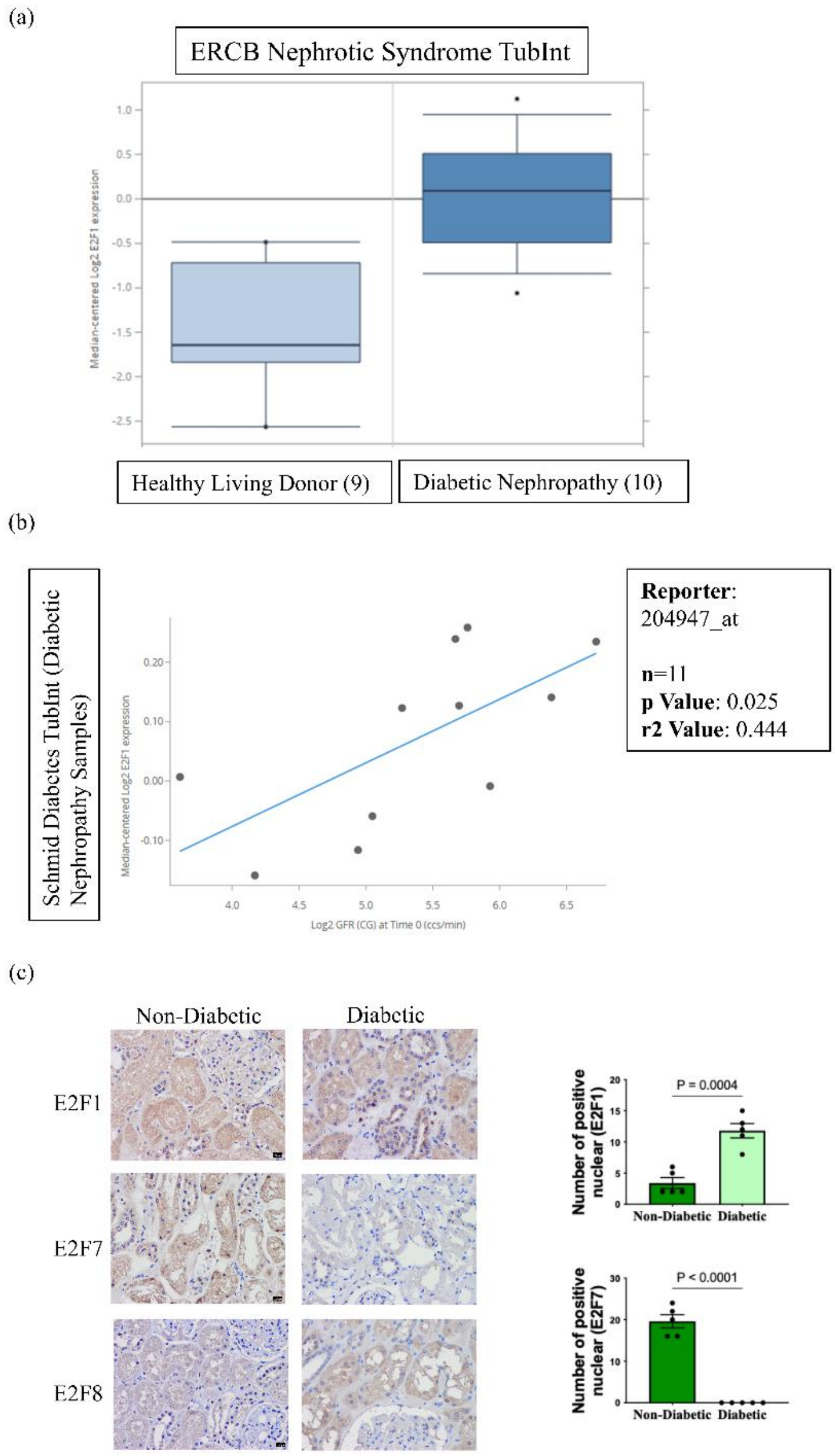

Accordingly, we have revised the manuscript in limitation section at Line 502-509, Page 21:

Several limitations of this study warrant consideration. Notably, tubulointerstitial E2F1 expression exhibited an unexpected positive correlation with GFR within the DKD cohort. We posit that this divergence from our in vivo findings reflects temporal disparities in disease progression. More specifically, animal models likely recapitulate early-stage pathogenesis, whereas the clinical cohort predominantly represents advanced disease. Future clinical investigations are required to systematically delineate the relationship between E2F expression dynamics, the severity of renal tubular injury, and the histological stage of interstitial fibrosis.

Minor concerns

1. The tubular localization of E2F7, E2F8, and E2F1 remains unclear. I would suggest including co-localization studies using confocal microscopy with segment-specific markers (e.g., for proximal tubules, distal tubules, or collecting ducts).

Response: We thank the reviewer for this constructive suggestion, which enhances the spatial resolution and physiological relevance of our study. We agree that precisely defining the tubular localization of these transcription factors is critical for understanding their role in DKD). To address this, we performed multiplex immunofluorescence on kidney sections from *db/db* and wild-type control mice. We utilized well-established, segment-specific markers to delineate the tubular architecture: aquaporin-1 (AQP1) for proximal tubules. Co-immunostaining of kidney sections from db/db mice revealed robust colocalization of E2F1 with AQP1, an established marker of the proximal tubule and loop of Henle (**Fig. 2g**), indicating that tubular epithelial cells may involve in the E2F1 regulation process.

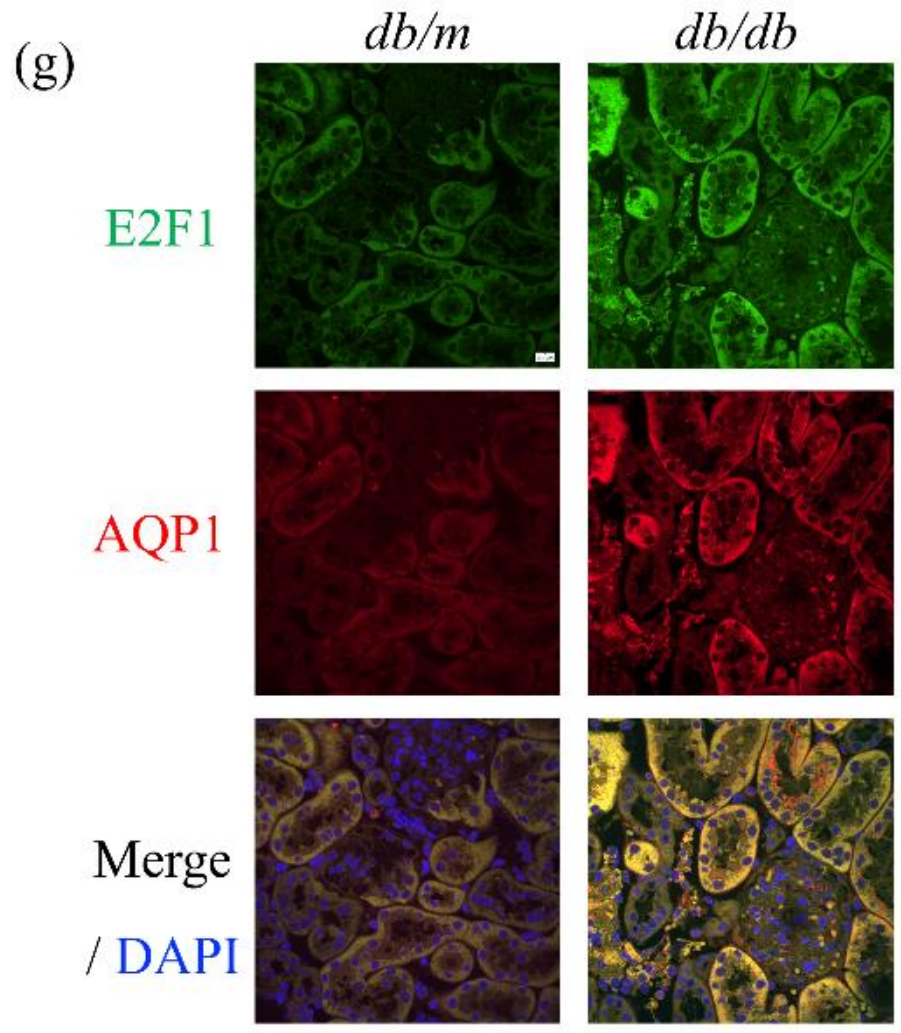

Accordingly, we have revised the manuscript in result section at Line 301-304, Page 13:
Notably, co-immunostaining of kidney sections from db/db mice revealed robust colocalization of E2F1 with AQP1, an established marker of the proximal tubule and loop of Henle (Fig. 2g), indicating that tubular epithelial cells may involve in the E2F1 regulation process.

2. There were no obvious changes in the staining of Masson in Fig. 1d and Sirius Red in Fig. S1d of the db/db mice with resveratrol treatment.

Response: Accordingly, we have improved the representative images via replaced the original micrographs in Fig. 1d (Masson's trichrome) and Fig. S1d (Picrosirius Red) with clear-resolution images.

3. In Fig. 6a and 6b, the P values in the statistical graphs were covered and not clearly visible.
Response: Accordingly, we have improved the images for clear version.

4. In Fig. 2a, the anti-senescence effect of resveratrol is only supported by SA-β-gal staining. I would suggest including additional senescence markers (e.g., p21 and γH2AX) to provide more comprehensive evidence for the anti-senescent effect of resveratrol.

Response: Accordingly, we have added the results of p21 to support the anti-senescence effect of resveratrol (**Fig. 2b**).

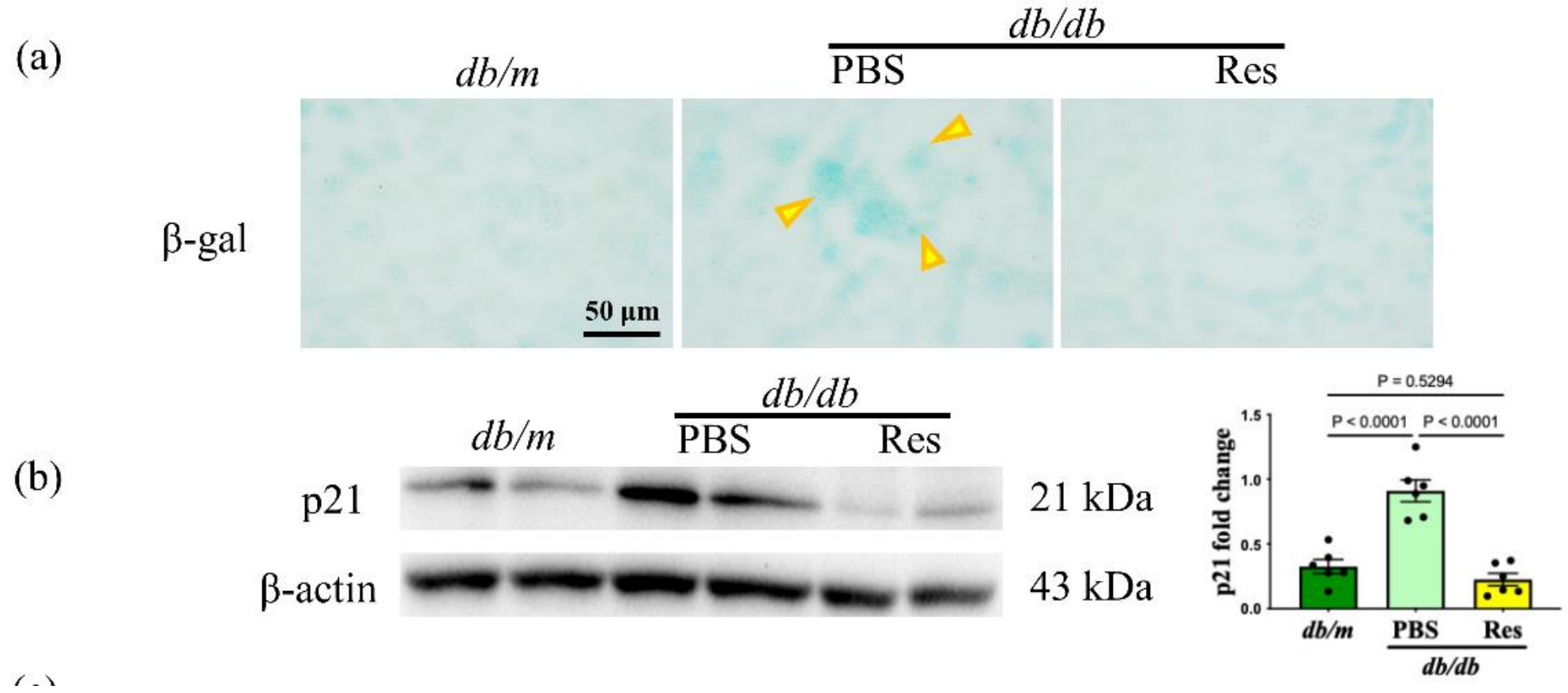

Accordingly, we have revised the manuscript in result section at Line 285-287, Page 12:
Consistent with this histological observation, resveratrol attenuated the expression of the senescence marker p21 in kidney tissues (Fig. 2b).

5. The authors propose that SASP factors released by E2F1-induced senescent cells contribute to renal fibrosis in the manuscript and Fig. 7. However, the expression of SASP factors was not examined. It is suggested that the authors detect the expression of typical SASP factors using qPCR or ELISA.

Response: We agree with the reviewer that directly quantifying the senescence-associated

secretory phenotype (SASP) is essential to substantiate our proposed mechanism linking E2F1-induced senescence to renal fibrosis. To address this critical point, we performed ELISA to evaluate the secreted protein levels of classical SASP components. Specifically, we measured Interleukin-1 beta (IL-1β), Interleukin-6 (IL-6), Tumor Necrosis Factor-alpha (TNF-α). (**Fig. 2c**).

(c)

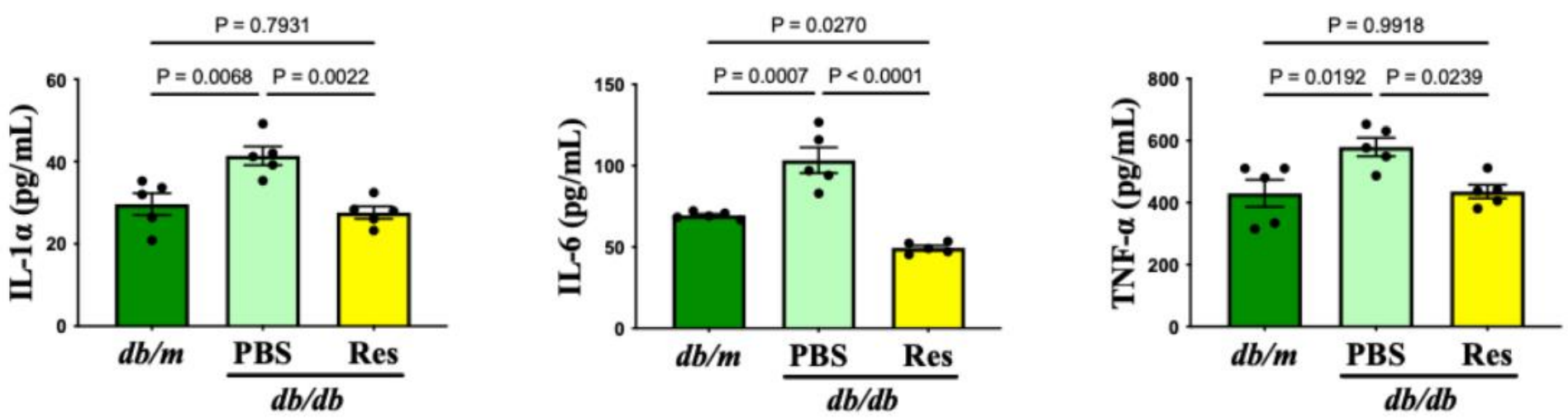

Accordingly, we have revised the manuscript in result section at Line 287-289, Page 12: Furthermore, ELISA analysis confirmed that resveratrol treatment suppressed the renal levels of senescence-associated secretory phenotype (SASP) factors, specifically IL-1α, IL-6, and TNF-α (Fig. 2c).

6. The supplemental figures are not presented in a logical order and the Fig. S1e, as referenced in the text, is missing from the supplemental materials. Please reorganize all supplemental panels in sequential order and ensure that all referenced figures are included.

Response: We thank the reviewer for pointing out this important point and we have reorganized all supplemental panels accordingly.

7. In Fig 5b and d, there were no obvious changes in the expression of E2F7 and E2F8 in mRTEC with E2F7 overexpression or E2F8 overexpression. Please explain that.

Response: Accordingly, we have improved the representative images via replaced with the original micrographs in new Fig. 6b, d.

(b)

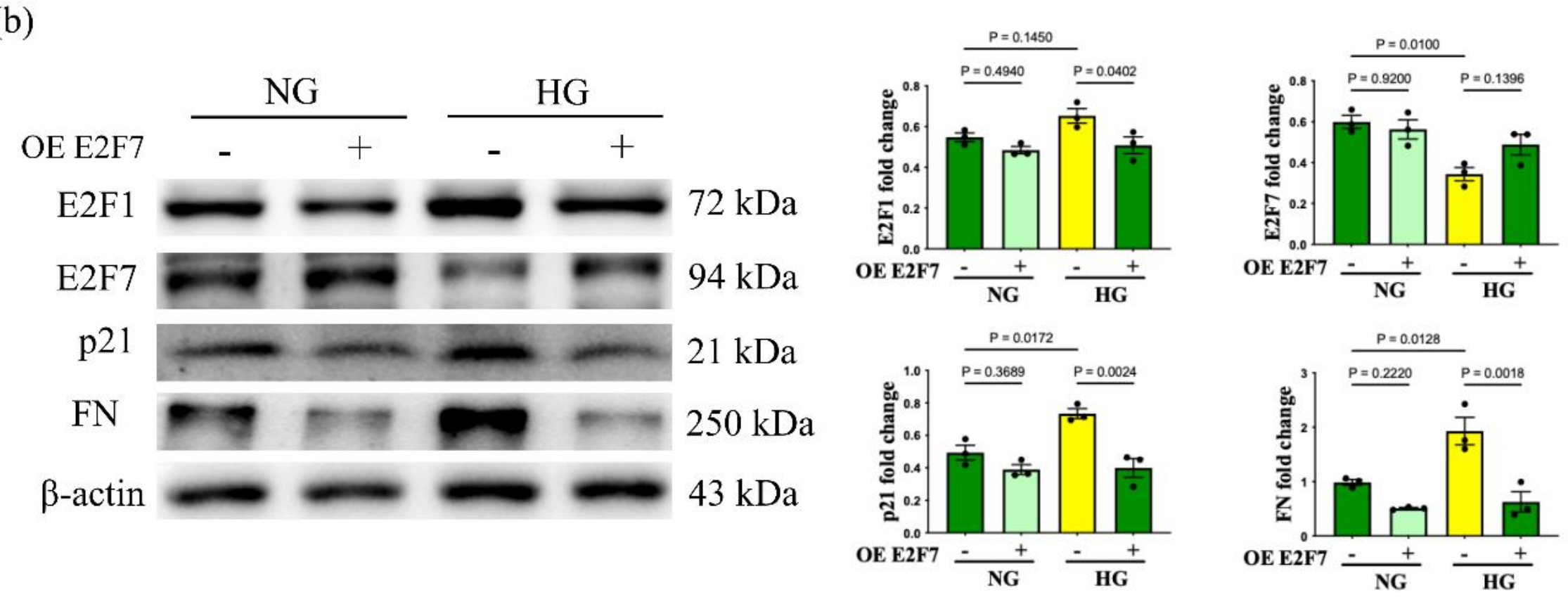

(d)

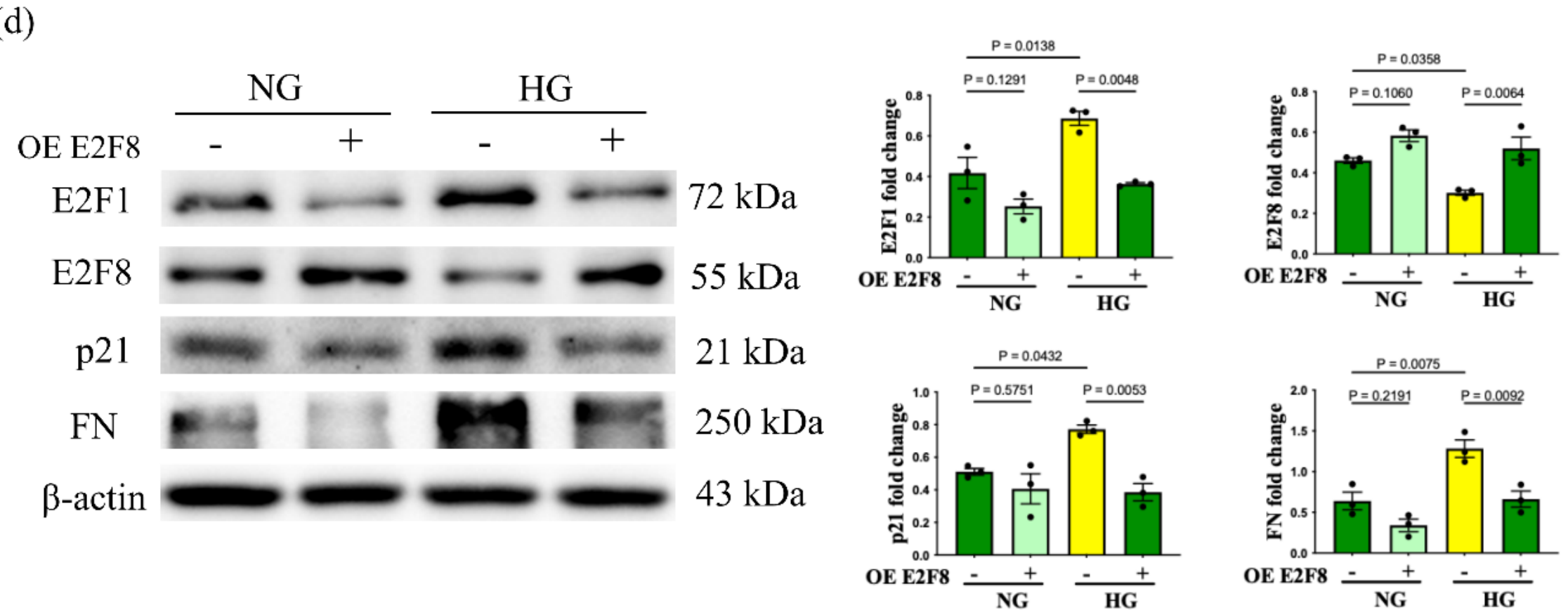

8. The manuscript only used the db/db mouse model to investigate DKD pathogenesis and therapeutic effects. However, validating the key findings in a second model, such as STZ-induced diabetic mice, would significantly strengthen the generalizability of the conclusions.

Response: We thank the reviewer for this highly constructive suggestion. We agree that validating our findings in an independent, well-established model of DKD substantially bolsters the generalizability and translational relevance of our conclusions. In direct response to this comment, we have expanded our *in vivo* studies to include the streptozotocin (STZ)-induced Type 1 diabetic rat model.

Surprisingly, kidney tissue section from STZ rat model did not replicate the findings observed in the *db/db* model. Our new section staining observed that neither E2F1 nor E2F7 was changed when compared to control rat group (data not shown), indicating that special regulation of E2F family mainly exists in Type-II diabetes. Further experiments to understand this interesting finding are needed.

Reviewer 2:

In this manuscript, Li et.al. investigate the E2F1-E2F7/8 regulatory axis and its pivotal role in hyperglycemia-induced tubular cellular senescence. The study reveals that elevated E2F1 expression in diabetic nephropathy (DN) models activates DNA damage response (DDR) pathways, subsequently driving cellular senescence. The authors demonstrate that the atypical E2F family members, E2F7 and E2F8, repress E2F1 transcription and translation by binding to and masking the E2F1 promoter. Additionally, they identify resveratrol as a pharmacological modulator of this E2F axis.
Overall, the study offers valuable mechanistic insights into diabetic kidney disease (DKD) through a coherent experimental design. The findings carry significant translational potential. However, several major points regarding phenotypic validation, mechanistic directness, and in vivo physiological relevance require further clarification and experimental support before acceptance for publication:

Response: We thank the reviewer for finding our study of interest, please find our response to the concerns as follows.

1. Quantitative validation of cell cycle arrest is required. Cell cycle arrest is a definitive hallmark of cellular senescence. While the authors have provided marker-based evidence, the manuscript would be significantly strengthened by direct quantification of cell cycle distribution: Incorporate flow cytometric analysis (e.g., PI staining or BrdU/EdU incorporation) to rigorously confirm the transition to a senescence-associated growth arrest in the tubular cell models.

Response: We thank the reviewer for this critical assessment. We fully agree that direct functional quantification of cell cycle arrest is essential to substantiate our marker-based cellular senescence data. To address this, we conducted flow cytometric analyses on our RTEC. We utilized both Propidium Iodide (PI) staining to evaluate global cell cycle distribution. RTECs cultured under hyperglycemic conditions exhibited a significant accumulation of cells in the S-phase population. This indicates a robust S phase cell cycle arrest. Notably, ectopic expressions of either E2F7 or E2F8 effectively rescued mRETCs from high-glucose-induced S-phase arrest (**Fig. S6a**)

(a)

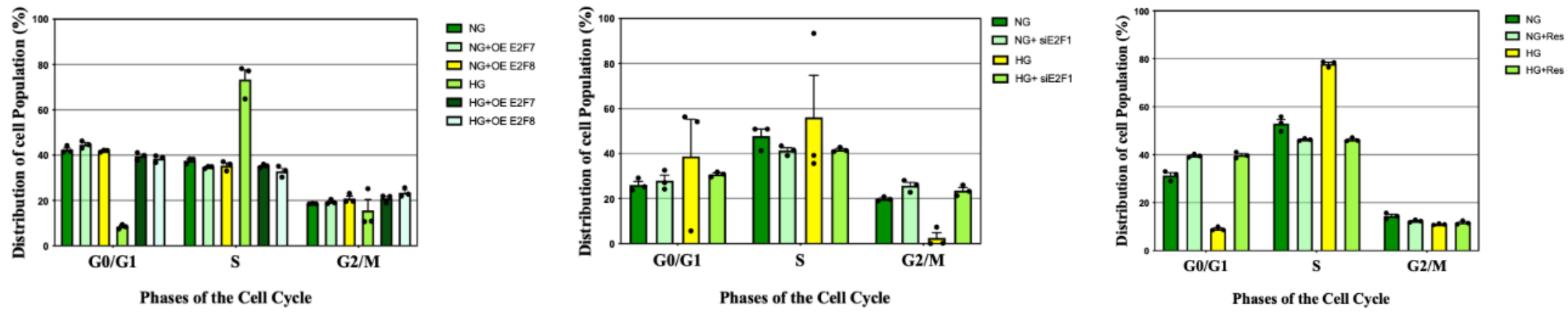

Accordingly, we have revised the manuscript in result section at <u>Line 383-384, Page 16:</u>

Notably, ectopic expressions of either E2F7 or E2F8 effectively rescued mRETCs from high-glucose-induced S-phase arrest (Fig. S6a).

2. The authors establish that E2F1 knockdown reduces phosphorylation within the ATM/CHK2 pathway and that E2F7/8 overexpression suppresses E2F1. However, the connection between E2F7/8 upregulation and the attenuation of the DDR remains associated: Authors should provide direct evidence demonstrating that overexpressing E2F7 and E2F8 significantly decreases the phosphorylation levels of ATM and CHK2. This is essential to confirm that the E2F7/8-E2F1 axis is the primary driver of the observed DDR modulation and bridging the Mechanistic Gap between E2F7/8 and the ATM/CHK2 Axis.

Response: We thank the reviewer for highlighting this critical mechanistic gap. We agree that direct evidence linking E2F7 and E2F8 upregulation to the attenuation of the ATM/CHK2-mediated DNA damage response (DDR) is necessary to firmly establish the E2F7/8-E2F1 axis as the primary driver of this phenotype. To address this concern, we conducted additional *in vitro* experiments in the revised manuscript. We transiently overexpressed E2F7 and E2F8 in RTECs cultured under hyperglycemic conditions and evaluated the phosphorylation levels of ATM and CHK2. Notably, ectopic expressions of either E2F7 or E2F8 concomitantly attenuated activation of the ATM/CHK2 signaling axis (**Fig. S6c**).

(c)

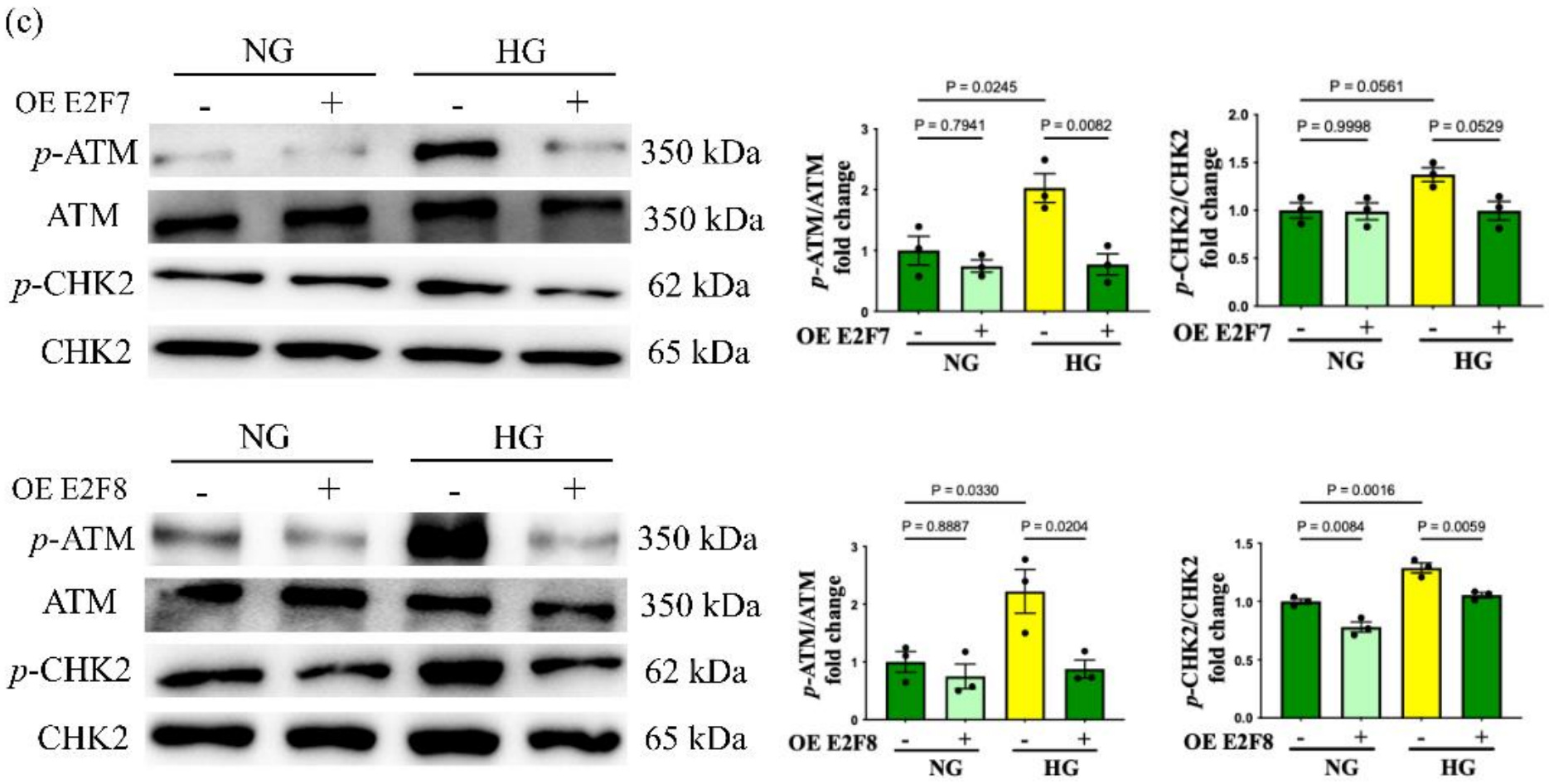

Accordingly, we have revised the manuscript in result section at Line 383-387, Page 16:
Notably, ectopic expressions of either E2F7 or E2F8 effectively rescued mRETCs from high-glucose-induced S-phase arrest (Fig. S6a) and concomitantly attenuated activation of the ATM/CHK2 signaling axis (Fig. S6c). Collectively, these findings demonstrate that E2F7 and E2F8 suppress senescence and fibrotic remodeling in renal tubular epithelial cells via the targeted downregulation of E2F1 expression.

3. The study concludes that E2F7/8 suppresses E2F1 by binding to its promoter region. To solidify this claim of transcriptional regulation, authors should provide E2F1 mRNA expression data (via RT-qPCR) following E2F7/8 overexpression. This will clarify whether the observed reduction in E2F1 protein levels is a direct consequence of transcriptional repression or involves secondary post-translational mechanisms.

Response: We thank the reviewer for this insightful comment. To address this, we conducted new RT-qPCR experiments to quantify E2F1 mRNA expression in RTECs following the overexpression of E2F7 and E2F8 under high-glucose conditions.
E2F7 overexpression downregulating E2F1 expression (**Fig. S6b**). Parallel experiments demonstrated that E2F8 overexpression recapitulates this phenotype (**Fig. S6b**).

(b)

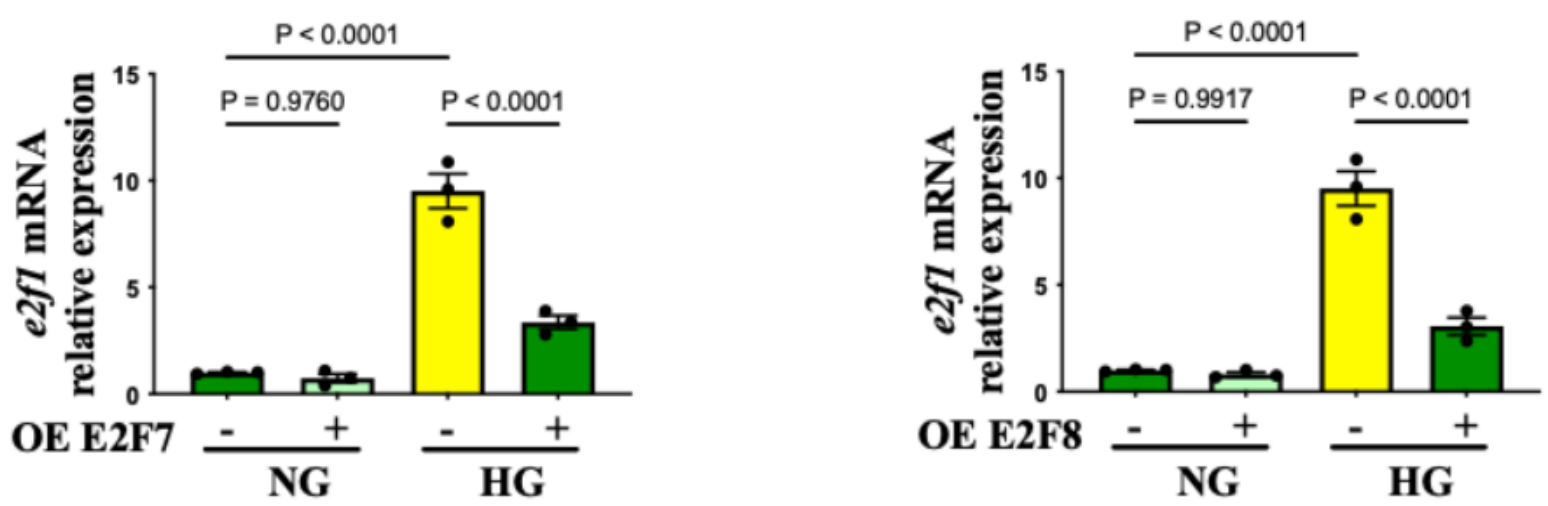

Accordingly, we have revised the manuscript in result section at Line 377-380, Page 16:
E2F7 overexpression mitigated cellular senescence and fibrosis while downregulating E2F1 expression (Fig. 6a-b, S6b). Parallel experiments demonstrated that E2F8 overexpression recapitulates this phenotype (Fig. 6c-d, S6b).

4. Physiological Characterization of the in vivo Model
The use of db/db mice with E2F1 knockdown is central to this paper's logical framework. However, the manuscript lacks an assessment of whether this genetic intervention actually preserves renal function. Authors should provide data on renal functional parameters (e.g., urinary albumin-to-creatinine ratio, serum creatinine) and histological analysis (e.g., PAS

staining for basement membrane thickening or fibrosis quantification) comparing E2F1-knockdown db/db mice to their db/m counterparts.

The study presents a compelling mechanism for tubular senescence in DKD. I recommend that Editors consider this manuscript for publication provided the authors address these concerns with additional experimental data and refined analysis.

Response: We thank the reviewer for highlighting this important point. We fully agree that demonstrating the physiological and structural preservation of renal function in E2F1-knockdown *db/db* mice is essential to rigorously substantiate our molecular findings. To address this, we have now incorporated the previous published *in vivo* renal functional parameters comparing db/m controls, unmanipulated db/db mice, and E2F1-knockdown db/db mice. Compared to the *db/m* group, the BUN and MAU indexes in the *db/db* group showed significant increase, however the levels were significantly decreased in the AAV-shE2F1group (Table from ***Front Pharmacol. 2022 Jun 23:13:926211.***)

Table

Group	*db/m*	*db/db*	AAV-shE2F1 *db/db*
BUN (mmol/l)	2.92 ± 0.82	8.68 ± 1.48	4.66 ± 0.36
MAU (ng/ml)	8.52 ± 1.79	131.89 ± 17.08	27.27 ± 5.77

Accordingly, we have revised the manuscript in result section at Line 356-357, Page 15:

Serum biochemical characterization indicated that E2F1 silencing significantly alleviated serum BUN and MAU expression *in vivo*.

Reviewer 3:

This study focuses on the regulatory mechanism of renal tubular epithelial cell senescence in diabetic kidney disease (DKD). It demonstrates that E2F1 promotes senescence by inducing DNA damage, while E2F7 and E2F8 exert a protective effect through transcriptional inhibition of E2F1. Furthermore, it reveals that resveratrol may exert an anti - fibrotic effect by regulating this axis. The overall study design is reasonable, with diverse experimental approaches and abundant data, holding significant scientific significance and potential clinical application value. However, the study could be further strengthened with more in-depth mechanistic dissection, stricter experimental rigor, and improved data presentation. Specific comments are as follows:

Response: We thank the reviewer for pointing out critical suggestions that certainly improve the study, please find our response to the concerns as follows.

1. The authors demonstrate that E2F7/8 bind to the E2F1 promoter and inhibit its transcription via ChIP-qPCR and dual-luciferase reporter assays. Nevertheless, the current data are solely derived from in vitro overexpression systems, and the binding of E2F7/8 to the E2F1 promoter at the chromatin level has not been verified in db/db mouse renal tissues. It is recommended to perform ChIP-qPCR experiments in renal tissues to enhance the in vivo relevance of this regulatory mechanism.

Response: We appreciate the reviewer's astute feedback regarding the necessity of *in vivo* validation. We fully agree that demonstrating the physical binding of E2F7 and E2F8 to the E2f1 promoter at the chromatin level in kidney tissues is critical for establishing the physiological and therapeutic relevance of our proposed mechanism.

To address this limitation, we performed newly chromatin immunoprecipitation followed by quantitative PCR (ChIP-qPCR) directly on renal cortical tissues harvested from wild-type (WT) and *db/db* mice (**fig. 7d**).

Consistent with our *in vitro* findings, these new *in vivo* assays reveal a significant enrichment of E2F7 and E2F8 at the native E2f1 promoter in the kidneys of WT mice tissue. Results are negative in the *db/db* mice kidney tissue due to very low expression of E2F7 and E2F8 and therefore showing not detectable.

(d)

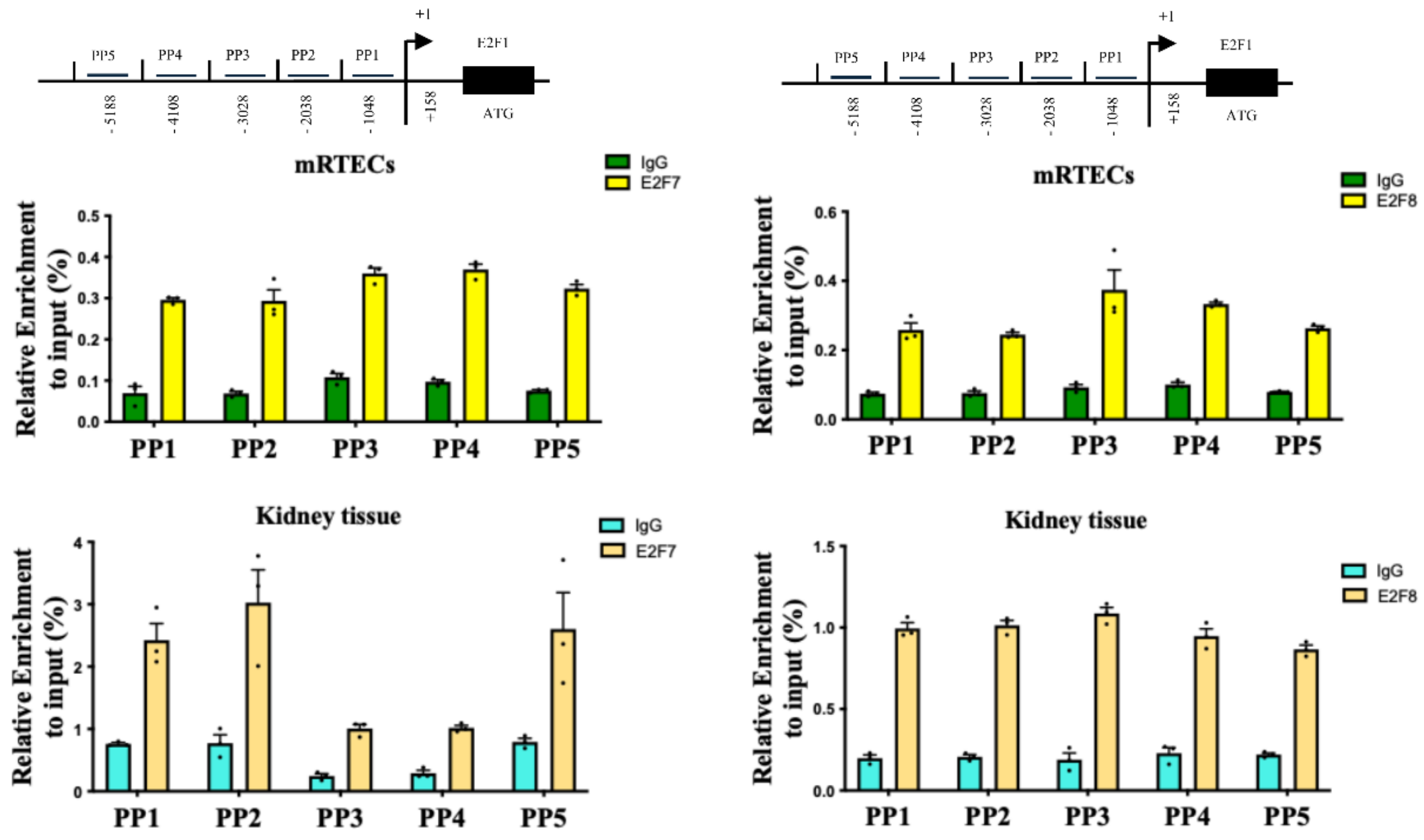

The corresponding text in the Results section has been updated to reflect these findings (<u>Page 17, Lines 403–405</u>):

Subsequent analysis indicated that E2F7 and E2F8 occupy multiple sites within this promoter region to blockade E2F1 production (Fig. 7d-e), both in mRTECs lysates and kidney tissue lysates. Collectively, these data indicate that E2F7 and E2F8 heterodimerize and bind the E2f1 promoter to repress expression, thereby alleviating HG-induced senescence and fibrosis in mRTECs.

2. The discussion mentions that senescent cells exacerbate renal injury through senescence-associated secretory phenotype (SASP) factors, but the study did not detect the expression and secretion of SASP-related factors, such as IL-6, TNF-α, MMP-9. It is suggested to supplement the detection of mRNA and protein levels of key SASP factors, and verify the paracrine effect of the supernatant from senescent mRTECs on normal mRTECs through conditioned medium experiments, so as to strengthen the evidence for the role of SASP in renal tubular injury.

Response: We thank the reviewer for this constructive suggestion. We agree that empirical validation of SASP factor secretion and its functional paracrine consequences is essential to substantiate our mechanistic claims regarding renal tubular injury. To address this, we have performed the recommended expression analyses and conditioned medium (CM) experiments. The newly generated data have been incorporated into the revised manuscript.

We quantified the protein levels of key SASP component via Elisa, as CCL-2, in mouse renal tubular epithelial cells (mRTECs). As suggested, we test CCL-2 levels in the supernatants of mRTECs treated with normal glucose (NG; 5 mM) or HG (30 mM) for 72 h. And then we apply normal mRTECs were cultured in conditioned medium (CM) from NG- or HG-treated cells for 48 h, then switched to fresh NG medium for 24 h prior to measuring secreted CCL-2 levels in the supernatant (**fig. S6d**).

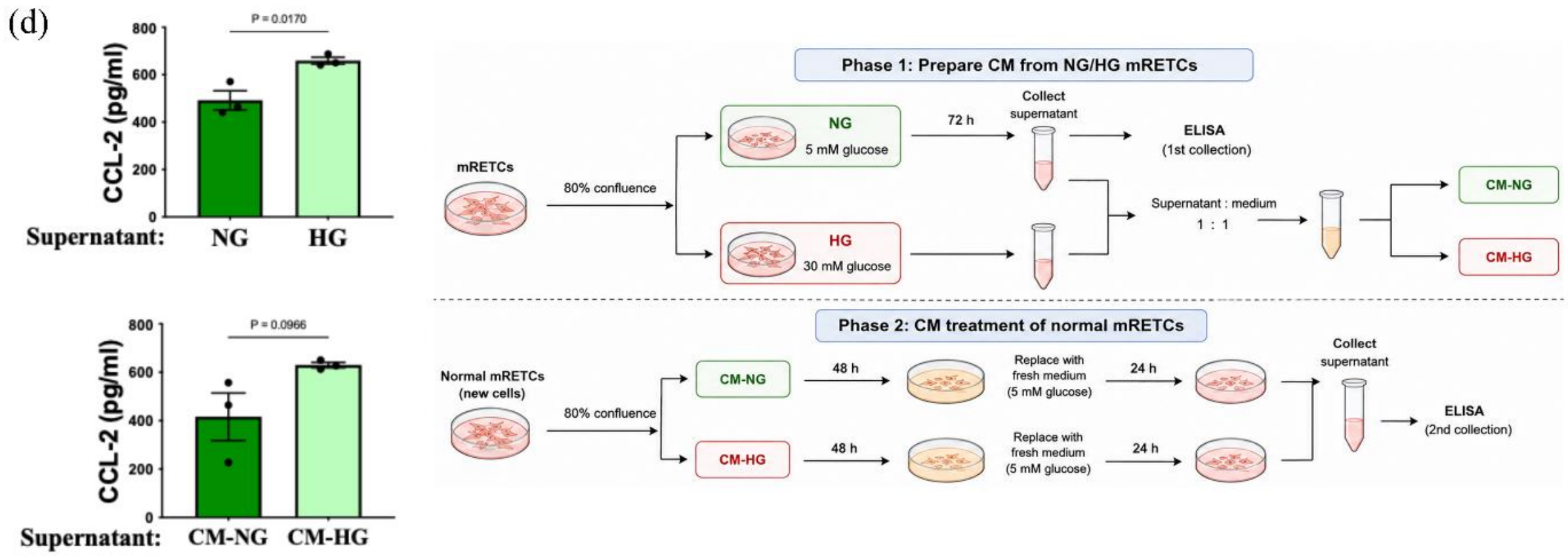

These new experiments rigorously confirm that diabetic mRTECs drives the secretion of a pathogenic SASP, which propagates secondary renal injury through paracrine mechanisms. The corresponding text in the discussion section has been updated to reflect these findings (Page 19, Lines 465–467):

Our results also suggested that SASP factors showing the paracrine effect from senescent mRTECs on normal mRTECs (**Fig. S6d**), supporting the evidence for the role of SASP in DKD renal tubular injury.

3. The study only verified the mechanism in mouse models and cell lines, lacking correlation validation with human DKD samples. It is recommended to supplement immunohistochemical/immunofluorescence analyses of renal biopsy tissues from DKD patients and normal controls, detect the expression levels of E2F1/E2F7/E2F8, and analyze their correlation with the degree of renal tubular injury and the stage of interstitial fibrosis.

Response: We appreciate the reviewer's critical assessment. We fully agree that validating our proposed mechanism in human clinical samples fundamentally strengthens the translational relevance of our findings. In direct response to this valuable suggestion, we have acquired human renal biopsy specimens and performed the recommended analyses.

Due to resource limitation, to address this comment, we obtained renal biopsy tissues from patients with biopsy-proven diabetic kidney disease (DKD, n = 1) and normal adjacent renal tissues from age-matched patients undergoing nephrectomy as healthy controls (n = 1). We conducted immunohistochemical (IHC) staining to quantify the expression and localization of E2F1, E2F7, and E2F8 (**Fig. S7c**).

(c)

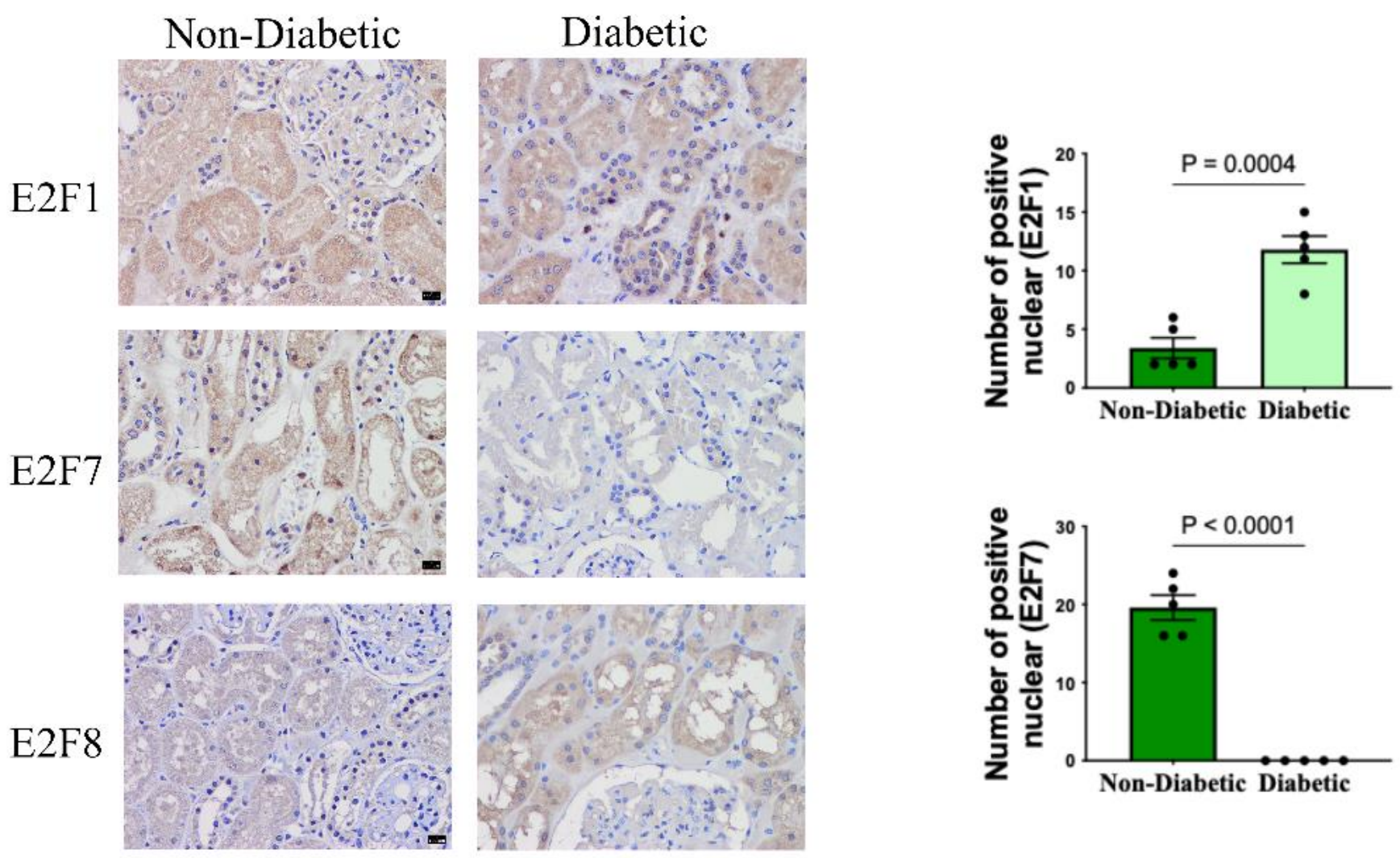

Consistent with our *in vitro* data, we observed a robust upregulation of E2F1 that strongly localized to the nuclei of RTECs in DKD patient compared to normal control. Conversely, the basal expression of the transcriptional repressors E2F7 was significantly diminished in the RTECs of the DKD cohort. E2F8 is not detectable in these cases.

Unfortunately, we cannot perform correlation analyses comparing the E2F staining with clinical pathology scores due to limit case. We added the corresponding text in the limitation section in (Page 21, Lines 502–509):

Several limitations of this study warrant consideration. Notably, tubulointerstitial E2F1 expression exhibited an unexpected positive correlation with GFR within the DKD cohort. We posit that this divergence from our in vivo findings reflects temporal disparities in disease progression. More specifically, animal models likely recapitulate early-stage pathogenesis, whereas the clinical cohort predominantly represents advanced disease. Future clinical investigations are required to systematically delineate the relationship between E2F expression dynamics, the severity of renal tubular injury, and the histological stage of interstitial fibrosis.

4. The study states that E2F1 knockdown can reduce the phosphorylation level of ATM/CHK2, but it remains unclear whether E2F1 directly binds to the promoters of these

genes. It is suggested to supplement ChIP experiments for verification, or analyze E2F1-regulated DNA damage-related genes through transcriptome sequencing to clarify its mechanism of action.

Response: We thank the reviewer for this highly insightful suggestion, which prompted us to rigorously clarify the causal relationship between E2F1 activity and the ATM/CHK2 DNA damage response (DDR) pathway. To address this, we performed a combined ChIP-seq analysis demonstrates E2F1 regulation of the ATM-CHK2 signaling axis via direct promoter binding. Analysis of ChIP-Atlas datasets identified E2F1 target genes bearing binding events within ±1 kb of the transcription start site (TSS). The plotted binding values reflect the average scores derived from MACS2 and STRING. Data filtering isolated genes specifically associated with the ATM signaling pathway that exceed a high-confidence binding score threshold (> 500). Notably, this stringent screening confirms both ATM and CHEK2 (CHK2) as direct regulatory targets of E2F1 (**Fig. S5**).

(a)

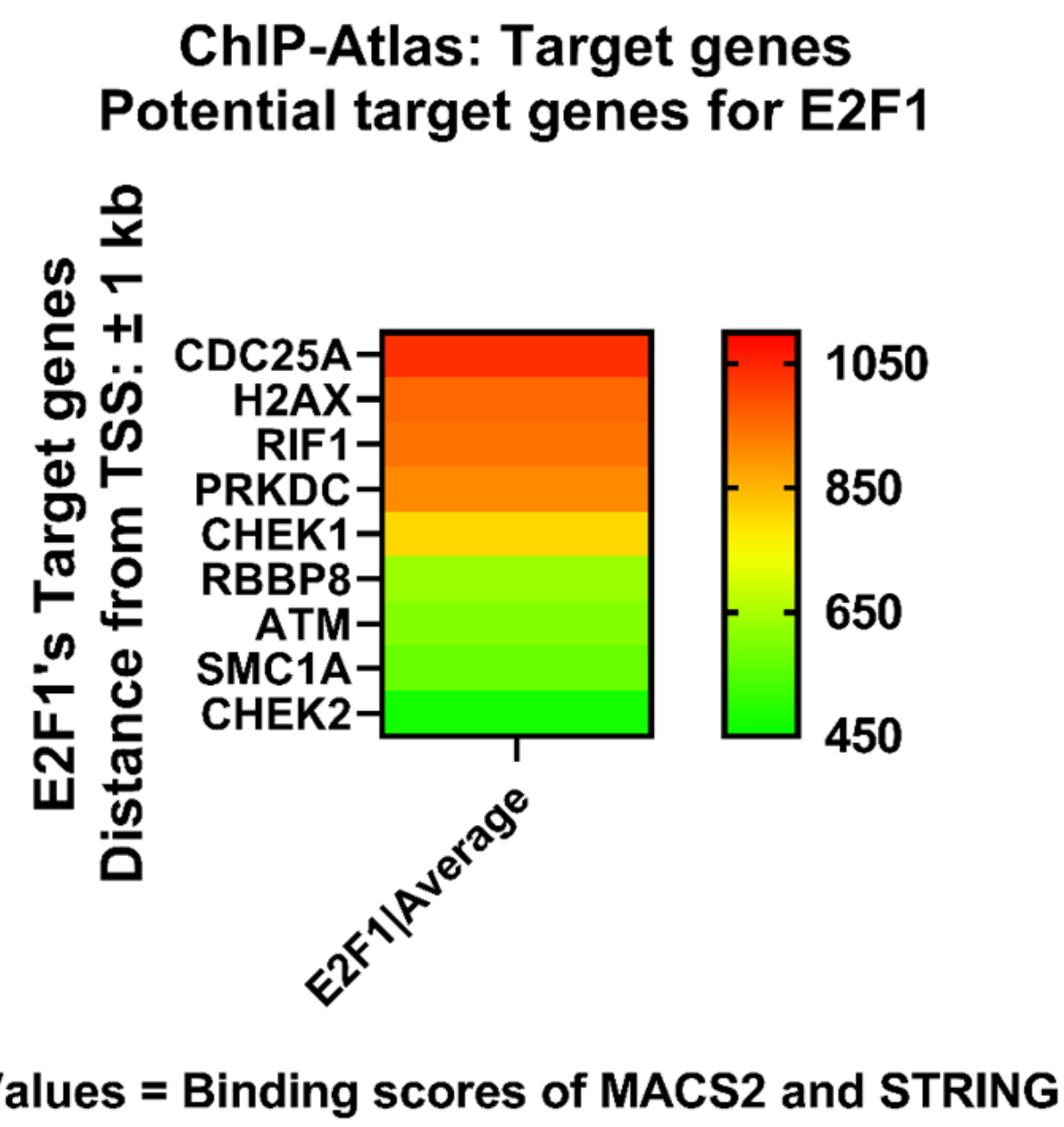

The corresponding text in the results section has been updated to reflect these findings (Page 15, Lines 361–365):

Interestingly, combined ChIP-seq analysis demonstrates E2F1 regulation of the ATM-CHK2 signaling axis via direct promoter binding (Fig. S5). E2F1 directly targets the promoter region of ATM and CHK2. Collectively, these data demonstrate that E2F1 promotes DNA damage via activation of the ATM/CHK2 pathway.

5. The study used 30 mmol/L high glucose to treat mRTECs. It is recommended that the

authors supplement the rationale for the selection of the 30 mmol/L high glucose concentration and treatment duration, which can be supported by citing relevant literature or providing pre-experimental data (time/concentration-dependent changes in cell viability and senescence marker expression) to enhance the scientific rigor of the experimental design.

Response: We appreciate the reviewer's constructive recommendation. We agree that explicitly detailing the selection of the 30 mmol/L glucose concentration and the corresponding treatment duration strengthens the rigorousness of our experimental design. In the revised manuscript, we have addressed this by incorporating both literature-based precedent and our pre-experimental validation data:

Literature Rationale: The use of 30 mmol/L D-glucose is a well-established and widely accepted paradigm for simulating the severe diabetic milieu in vitro, particularly for murine renal tubular epithelial cells (mRTECs).

Pre-experimental Validation: To empirically justify these parameters for our specific senescence model, we conducted comprehensive dose-response and time-course assays. Live cell quantification of mRTECs following exposure to the indicated glucose concentrations (5–40 mM) over a 72-h time course (24, 48, and 72 h). Relative nuclear expression of p21 in mRTECs subjected to dose- and time-dependent glucose treatments (5–40 mM; 24, 48, and 72 h) (**Fig. S2b, c**).

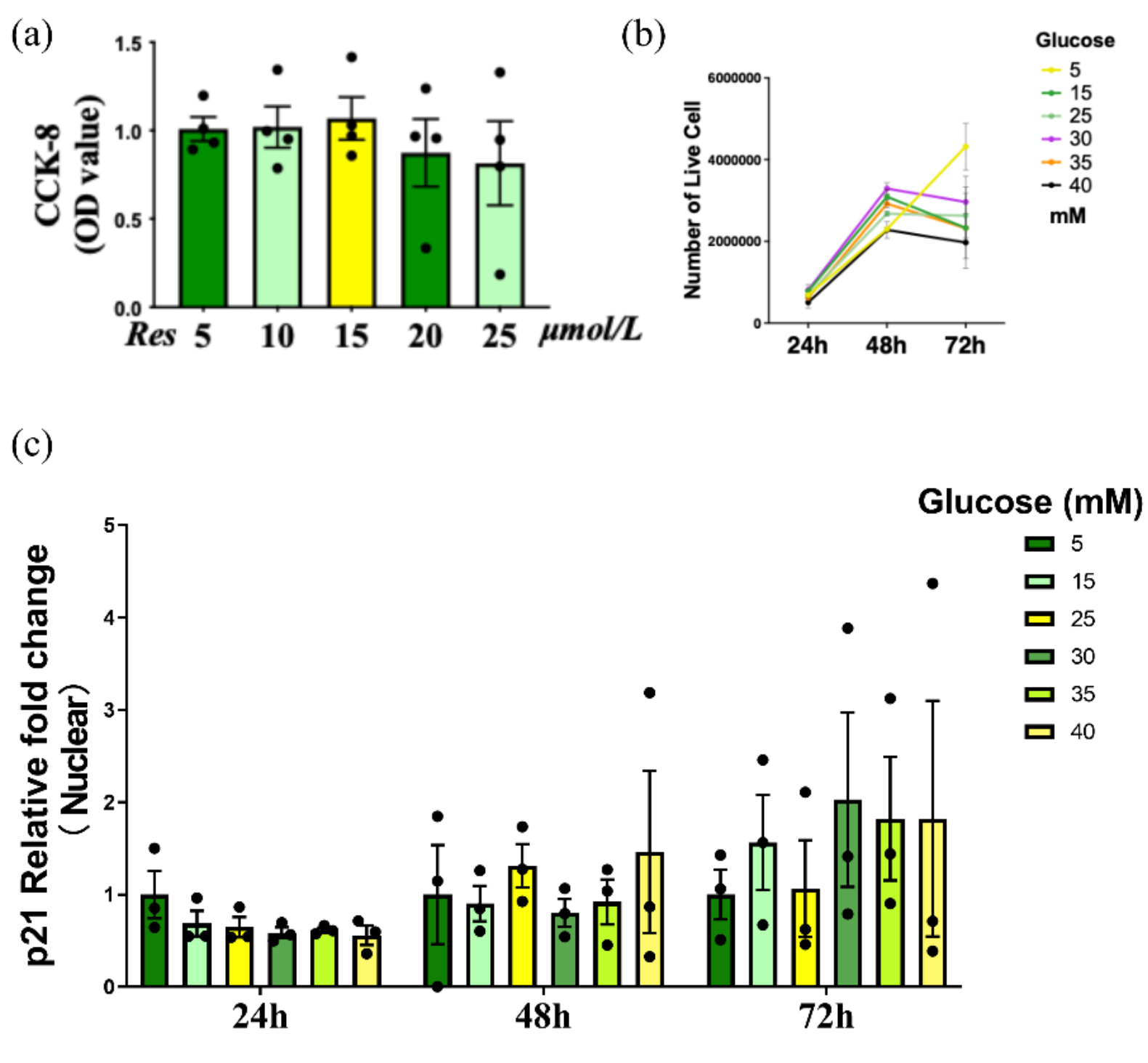

The corresponding text in the methods section has been updated to reflect these findings (Page 5, Lines 106–112):

For experimental treatments, cells were maintained in either normal glucose medium (NG; 5.5 mmol/L glucose) or high glucose medium (HG; 30 mmol/L glucose, Glucose Solution; Gibco, cat# A2494001) to mimic hyperglycemia conditions. Cultured RTECs were incubated in 30 mM glucose for 72 hours which was suggested by previous study. We selected these parameters based on prior literature and our preliminary in vitro data, demonstrating that this specific high-glucose regimen robustly induces nuclear p21 expression without significantly compromising cell viability (Fig. S2b, c).

Point-by-point response to reviewers

<u>Reviewer 1:</u>

Basically, the authors have answered questions.

Response: We thank the reviewer for pointing out important suggestions that certainly improve the study.

<u>Reviewer 3:</u>

My concerns have been addressed. No more comments.

Response: We thank the reviewer for giving important comments for better understanding of current study.
