# Supplementary material for: E2F7 and E2F8 attenuate diabetic kidney disease by suppressing E2F1-induced tubular senescence
Source: Commun Biol. 2026 Nov 13;9:1526. doi: 10.1038/s42003-026-11334-1 (PMC13768344; doi:10.1038/s42003-026-11334-1)

Supplementary

E2F7 and E2F8 attenuate diabetic kidney disease by suppressing E2F1-induced renal tubular epithelial cells senescence

Zhiyang Li [1, 2, 3, 4, #], Yunli Dai [1, 3, 4, #], Dan Liang [1, 3, 4, 6], Yawen Xiao [1, 3, 4], Li Hu [1, 3, 4, 6], Xin Wu [1, 3, 4], Zhaowei Feng [1, 3, 4], JiaJia Chen [1, 3, 4], Shengjie Chen [1, 3, 4], Zhiping Yuan [7], Mingjun Shi [1, 3, 4], Yuanyuan Wang [1, 3, 4], Lingling Liu [1, 3, 4], Pengchen Chen [1, 3, 4], Shihai Jiang [2, *], Bing Guo [1, 3, 4, 5, *], Ying Xiao [1, 3, 4, *]

[1] Guizhou Provincial Key Laboratory of Pathogenesis and Prevention of Common Chronic Diseases, Guizhou Medical University, Guiyang, China.

[2] Institute of Laboratory Medicine, Clinical Chemistry and Molecular Diagnostics, Universitätsklinikum Leipzig, Leipzig, Germany.

[3] International Scientific and Technological Cooperation Base of Pathogenesis and Drug Research on Common Major Diseases, Guizhou Medical University, Guiyang, China.

[4] Guizhou Province Talent Base of Research on the Pathogenesis and Drug Prevention and Treatment for Common Major Diseases, Guizhou Medical University, Guiyang 550004, Guizhou, China.

[5] Collaborative Innovation Center for Prevention and Control of Endemic and Ethnic Regional Diseases Co-constructed by the Province and Ministry, Guizhou, China.

[6] The Second People’s Hospital of Guiyang, Guiyang, China.

[7] University Town Hospital, Guizhou Medical University, Guiyang, China.

# These authors contribute equally to this work.

* Correspondence

Supplementary Table 1.

Primer list of qPCR and CHIP-qPCR (*Mus musculus*)

Gene Name	Sequence
E2F7+F	CCGAGTGTGTGCAAAAGA
E2F7+R	TCCAGGGAATAGGCTGAC
E2F8+F	CTAAACGGACAACCAGTCG
E2F8+R	GCACCATCGAAATCTGTGT
E2F1+F	GCGCATCTATGACATCACCA
E2F1+R	TCAAGCCGCTTACCAATCC
β-actin+F	CCTCACTGTCCACCTTCC
β-actin+R	GGGTGTAAAACGCAGCTC
E2F1-PP1+F	CATCCCTTACTCATTTCCTGG
E2F1-PP1+R	ACATCTCCGGTCTTAAGATCC
E2F1-PP2+F	GAGTAATGTGGTCAGAGTTGG
E2F1-PP2+R	CAATATTCAAGTCCCCAGGTC
E2F1-PP3+F	GCCTCATTTTCCCATCTGTAC
E2F1-PP3+R	CCCTACTCACGAACAGTTTG
E2F1-PP4+F	GCTGGGATTAAAGGTGTGAG
E2F1-PP4+R	AGGAGTACAATGGAGGTAGTG
E2F1-PP5+F	TGTCTTAGTCTGTTCCCCAC
E2F1-PP5+R	ACAAAGGCAAGCGTTCAG

Supplementary Table 2.

Primary antibody list used in present study

Name	Company	Cat No.
Fibronectin	Abcam (Abcam Plc)	ab2413
E2F1	Abcam (Abcam Plc)	ab112580
E2F8	Abcam (Abcam Plc)	ab109596
E2F7	Proteintech (Proteintech Group, Inc)	230073E3
Col III	Proteintech (Proteintech Group, Inc)	22734-1-AP
ATM	Absin (Absin Bioscience Inc)	abs131163
p-ATM	Absin (Absin Bioscience Inc)	abs140239
Chk2	Bioss (Bioss Biotechnology Co., Ltd)	bs-1391R
p-Chk2	Bioss (Bioss Biotechnology Co., Ltd)	bs-3721R
p21	Santa (Santa Cruz Biotechnology)	SC-6246
β-actin	PMK (Bioprimacy)	PMK058S
AQP1	Santa cruz	sc-55466
HyperFluor™ 488 GoatAnti-Rabbit IgG	APExBIO	K1206
HyperFluor™ 594 Goat Anti-Mouse IgG	APExBIO	K3306
Ubiquitin Recombinant Rabbit Monoclonal Antibody	HUABIO	ET1609-21

Supplementary Table 3.

The 35 annotated genes interacted with resveratrol. The pathway Recruitment and ATM-mediated phosphorylation of repair and signaling proteins at DNA double strand breaks (REACT:R-HSA-5693565) analyzed by CTD

Gene names				
ABL1	CHEK2	H2BC17	KAT5	RNF8
APBB1	EYA1	H2BC21	MAPK8	SMARCA5
ATM	EYA2	H2BC5	MRE11	SUMO1
BAP1	EYA3	H2BC6	NBN	TP53
BARD1	EYA4	H2BC8	NSD2	TP53BP1
BRCA1	H2AX	H4C13	RAD50	UBB
BRCC3	H2BC15	H4C6	RNF168	UBC

Supplementary Figure legend

Fig S1. Resveratrol mitigates renal fibrosis in the *db/db* mice model.

a) Serum biochemical analysis of *db/m* control and *db/db* mice. T-CHO, total cholesterol; TG, triglycerides (n=6). b) Representative image of kidney sections stained with HE staining, Sirius Red, and Periodic acid-Schiff (PAS) (n=6). Yellow arrows indicate damaged renal tubules. All data are represented as mean ± s.d. Data presented in (a) was analyzed via one-way ANOVA with Tukey's multiple comparisons test. All tests were two-sided. Source data are provided as a Supplementary Data 1file.

Fig S2. High glucose stimulation upregulates Vcam-1 expression in murine renal tubular epithelial cells (mRTECs).

a) Cell viability of mRTECs treated with escalating doses of resveratrol (5–25 µM), assessed via CCK-8 assay (n=4). b) Live cell quantification of mRTECs following exposure to the indicated glucose concentrations (5–40 mM) over a 72-h time course (24, 48, and 72 h) (n=3). c) Relative nuclear expression of p21 in mRTECs subjected to dose- and time-dependent glucose treatments (5–40 mM; 24, 48, and 72 h) (n=3). d) Vcam-1 expression levels in mRTECs in response to high glucose conditions (n=3). All data are represented as mean ± s.d. Source data are provided as a Supplementary Data 1file.

Fig S3. Resveratrol interacts directly with E2F7 and E2F8.

a) Molecular docking simulations predicting the binding conformations of resveratrol within the structural pockets of the E2F7 (left) and E2F8 (right) proteins. b) Cellular thermal shift assay (CETSA) demonstrating the thermal stabilization of E2F7 and E2F8 upon resveratrol binding. Cell lysates were incubated with 0.4 nM resveratrol or vehicle (1% DMSO) and subjected to a temperature gradient (37–62 °C). Soluble protein fractions were analyzed by immunoblotting, and band intensities were quantified via densitometry, normalized to the

respective 37 °C baseline samples (n=3). c) Representative immunoblots and corresponding densitometric quantification of Collagen-III and Fibronectin (FN) protein expression in mRTECs following treatment with 15 μM resveratrol. d) Representative immunoblots and densitometric analysis of p21 protein expression in mRTECs treated with 15 μM resveratrol. All data are represented as mean ± s.d. Data presented in (c-d) were analyzed via one-way ANOVA with Tukey's multiple comparisons test. All tests were two-sided. Source data are provided as a Supplementary Data 1file.

Fig S4. Resveratrol modulates the ATM-CHK2 signaling axis.

a) Reactome pathway enrichment analysis of resveratrol-interacting genes curated from the Comparative Toxicogenomics Database. Data display significantly enriched pathways (corrected $P < 0.01$). The pathway denoting the recruitment and ATM-mediated phosphorylation of repair and signaling proteins at DNA double-strand breaks (REACT:R-HSA-5693565) exhibits significant enrichment, encompassing 35 annotated genes mapped via the STRING database. b) Cytoscape network visualization of the ATM signaling pathway. The 35 annotated resveratrol-interacting genes identified in a are highlighted by red boxes.

Fig S5. Combined ChIP-seq analysis demonstrates E2F1 regulation of the ATM-CHK2 signaling axis via direct promoter binding.

a) Analysis of ChIP-Atlas datasets identified E2F1 target genes bearing binding events within ±1 kb of the transcription start site (TSS). The plotted binding values reflect the average scores derived from MACS2 and STRING. Data filtering isolated genes specifically associated with the ATM signaling pathway that exceed a high-confidence binding score threshold (> 500). Notably, this stringent screening confirms both ATM and CHEK2 (CHK2) as direct regulatory targets of E2F1.

Fig S6. Overexpression of E2f7 or E2f8 regulates the E2F1 and ATM-CHK2 signaling axis.

a) Flow cytometric analysis of cell cycle progression in mRTECs. Cells were exposed to high glucose (HG) with concurrent E2F7 or E2F8 overexpression (left), E2f1 knockdown (middle), or resveratrol treatment (right). Normal glucose (NG) conditions served as the control. b) RT-qPCR analysis of *E2f1* mRNA expression in mRTECs following the overexpression of E2f7 or E2f8. c) Immunoblot analysis and corresponding quantification of the DNA damage response (DDR) proteins ATM and CHK2 in mRTECs overexpressing E2f7 or E2f8. d) ELISA quantification of the senescence-associated secretory phenotype (SASP) factor CCL-2. Top: CCL-2 levels in the supernatants of mRTECs treated with normal glucose (NG; 5 mM) or HG (30 mM) for 72 h. Bottom: Normal mRTECs were cultured in conditioned medium (CM) from NG- or HG-treated cells for 48 h, then switched to fresh NG medium for 24 h prior to measuring secreted CCL-2 levels in the supernatant. All data are represented as mean ± s.d. Data presented in (b-c) were analyzed via one-way ANOVA with Tukey's multiple comparisons test. Data presented in (d) was Unpaired t test. All tests were two-sided. Source data are provided as a Supplementary Data 1file.

Fig S7. Clinical relevance of E2F1 expression in human diabetic nephropathy.

a) Relative E2F1 mRNA expression in the renal tubulointerstitium of healthy donors and patients with diabetic nephropathy. Transcriptomic data were retrieved from the European Renal cDNA Bank (ERCB) cohort via Nephroseq. b) Correlation between E2F1 expression levels and glomerular filtration rate (GFR, Cockcroft-Gault) in patient samples with diabetic nephropathy. Data was obtained from the Schmid dataset via Nephroseq. c) Representative immunohistochemical (IHC) staining and corresponding quantification of E2F1 and E2F7 protein levels in human kidney biopsy sections (non-diabetic control and diabetic nephropathy; n = 1 per group). All data are represented as mean ± s.d. Data presented in (c)

was Unpaired t test. All tests were two-sided. Source data are provided as a Supplementary Data 1file.

(a)

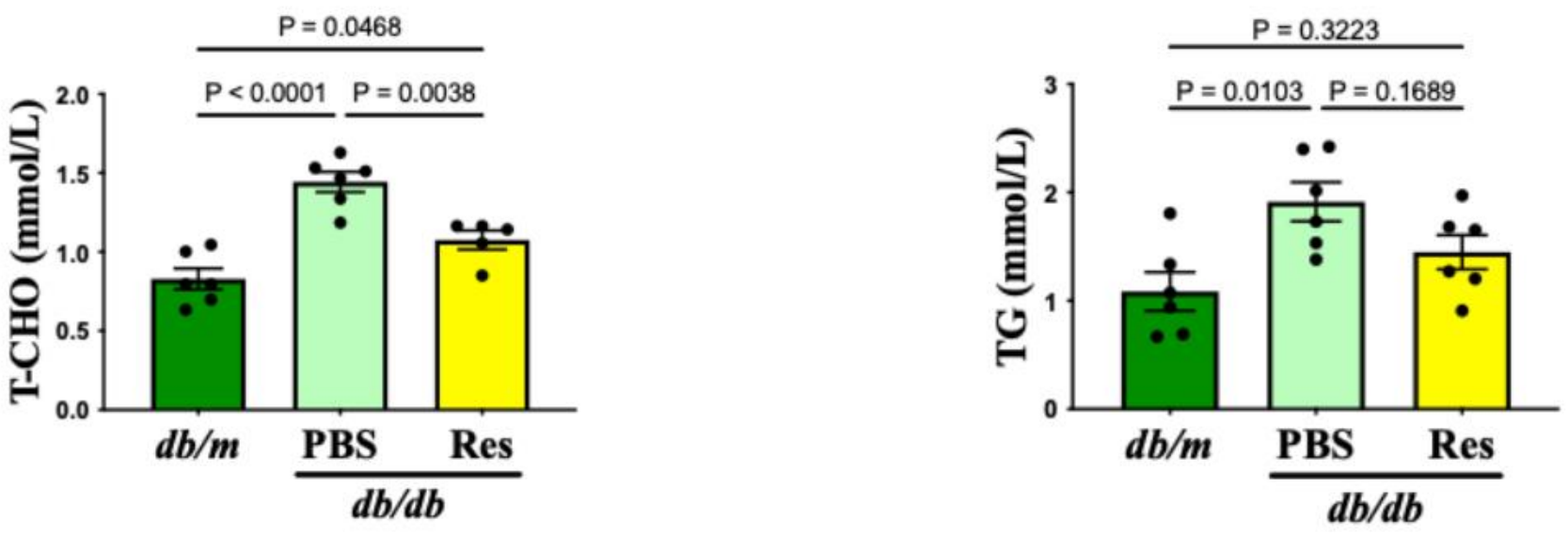

P = 0.0468
P < 0.0001
P = 0.0038
T-CHO (mmol/L)
2.0
1.5
1.0
0.5
0.0
db/m
PBS
Res
db/db
P = 0.3223
P = 0.0103
P = 0.1689
TG (mmol/L)
3
2
1
0
db/m
PBS
Res
db/db

(b)

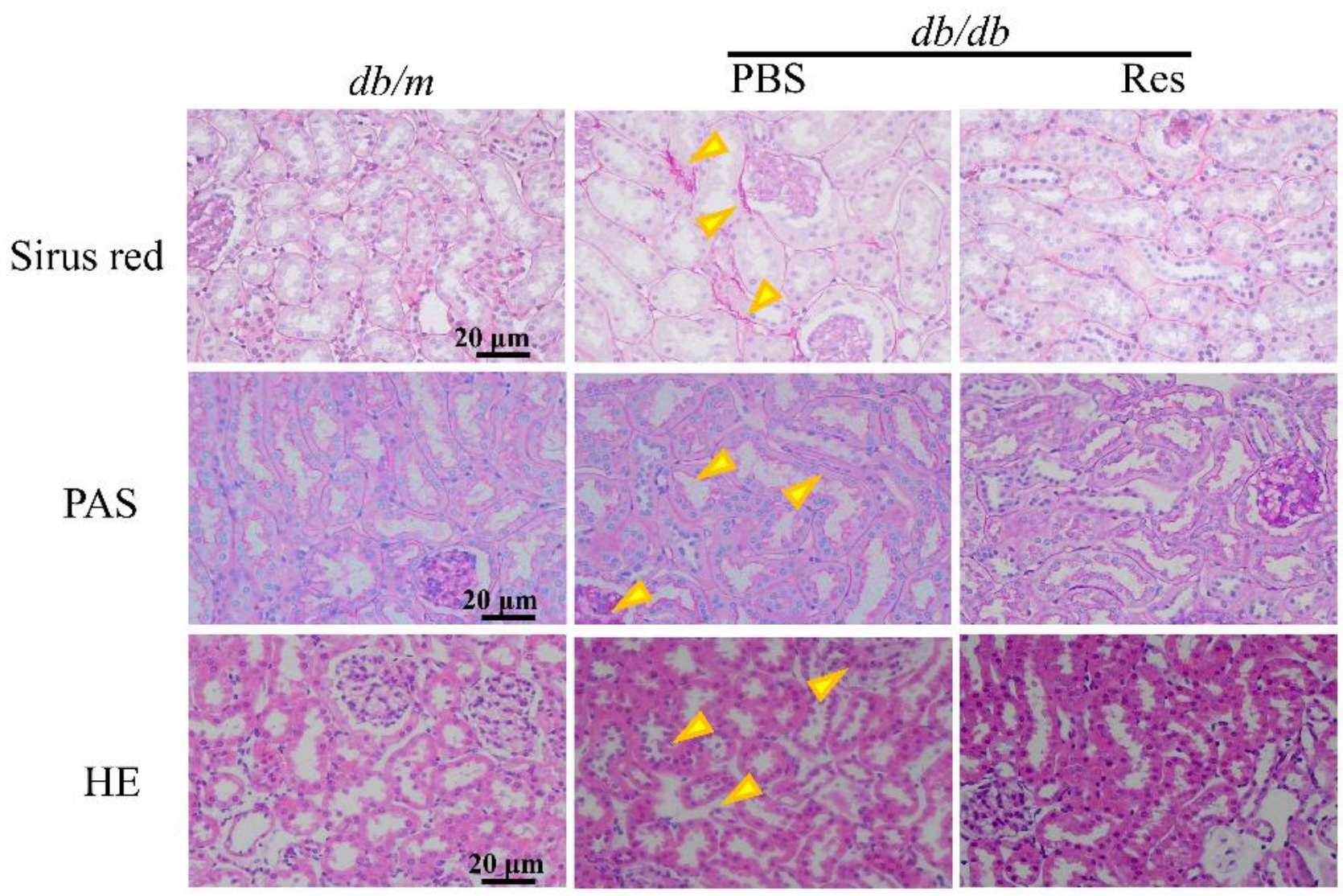

db/db
db/m
PBS
Res
Sirus red
PAS
HE
20 μm
20 μm
20 μm

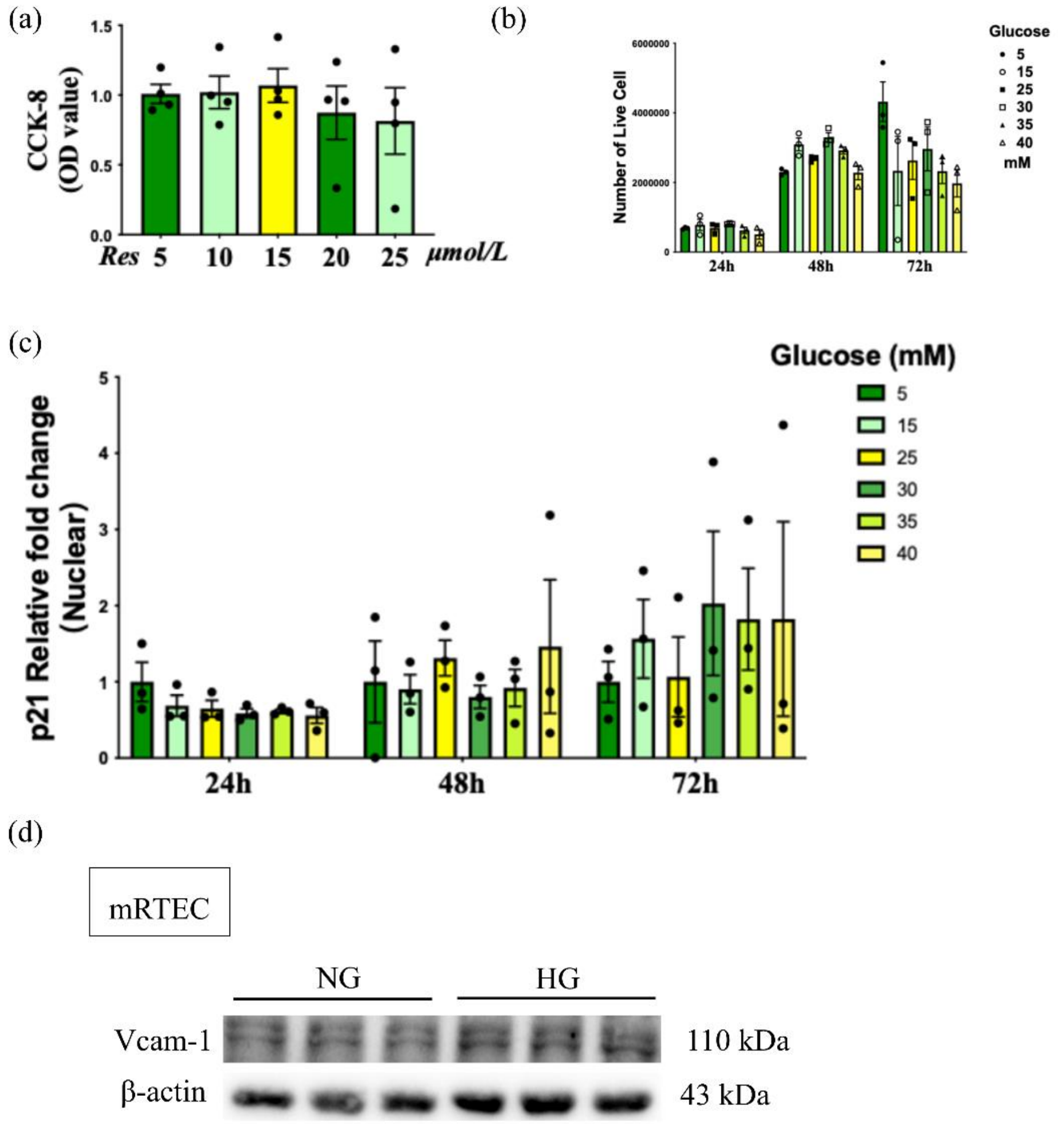
(a)
CCK-8
(OD value)
1.5
1.0
0.5
0.0
Res 5 10 15 20 25 μmol/L
(b)
Number of Live Cell
6000000
4000000
2000000
0
24h
48h
72h
Glucose
5
15
25
30
35
40
mM
(c)
p21 Relative fold change
(Nuclear)
5
4
3
2
1
0
24h
48h
72h
Glucose (mM)
5
15
25
30
35
40
(d)
mRTEC
NG
HG
Vcam-1
110 kDa
β-actin
43 kDa

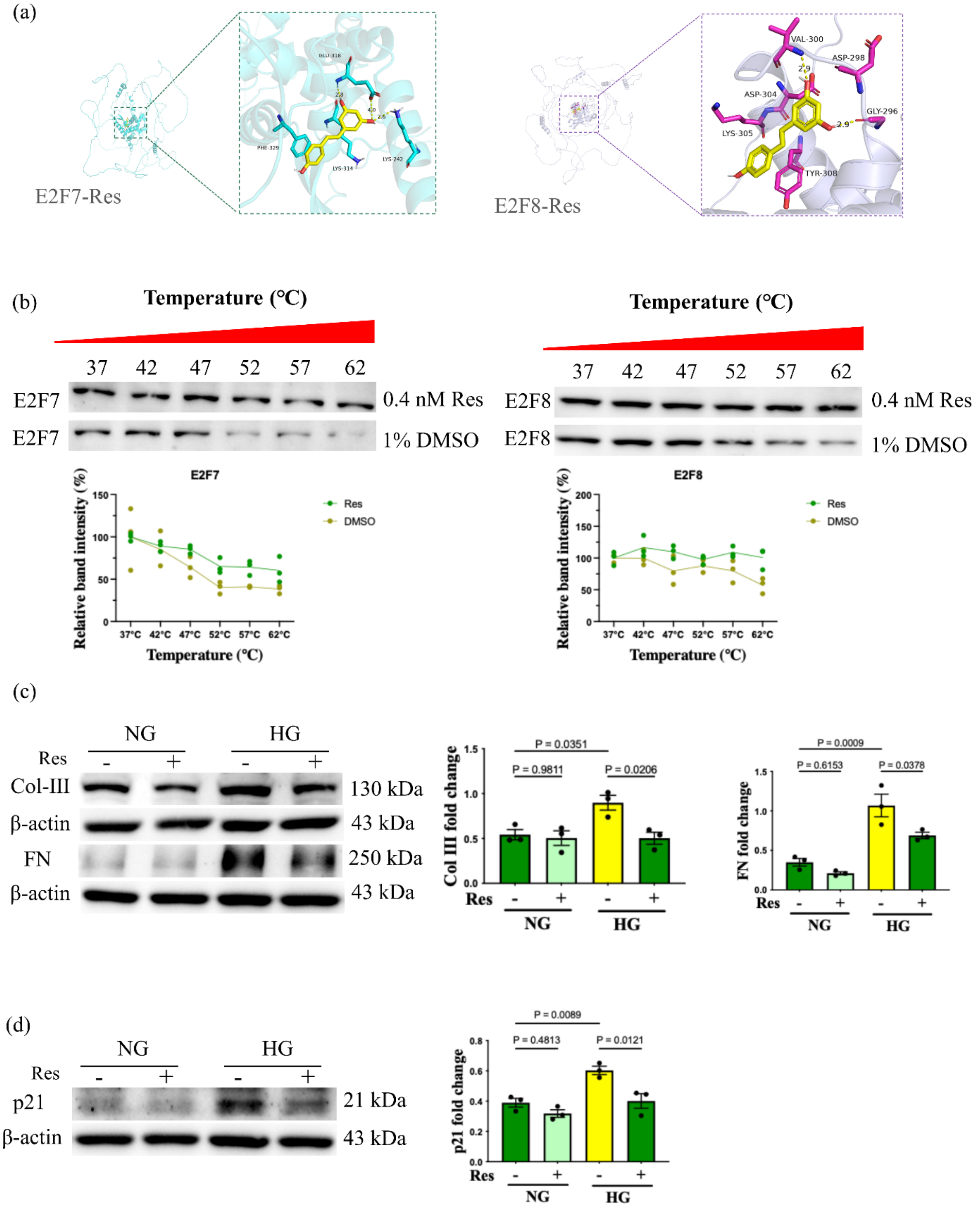
(a)
GLU-318
PHE-329
LYS-314
LYS-242
E2F7-Res
VAL-300
ASP-298
ASP-304
GLY-296
LYS-305
TYR-308
E2F8-Res
(b)
Temperature (℃)
37 42 47 52 57 62
E2F7
0.4 nM Res
E2F7
1% DMSO
E2F8
0.4 nM Res
E2F8
1% DMSO
E2F7
E2F8
Res
DMSO
Relative band intensity (%)
37℃ 42℃ 47℃ 52℃ 57℃ 62℃
Temperature (℃)
(c)
NG
HG
Res
Col-III
130 kDa
β-actin
43 kDa
FN
250 kDa
β-actin
43 kDa
P = 0.0351
P = 0.9811
P = 0.0206
Col III fold change
P = 0.0009
P = 0.6153
P = 0.0378
FN fold change
(d)
NG
HG
Res
p21
21 kDa
β-actin
43 kDa
P = 0.0089
P = 0.4813
P = 0.0121
p21 fold change

(a)

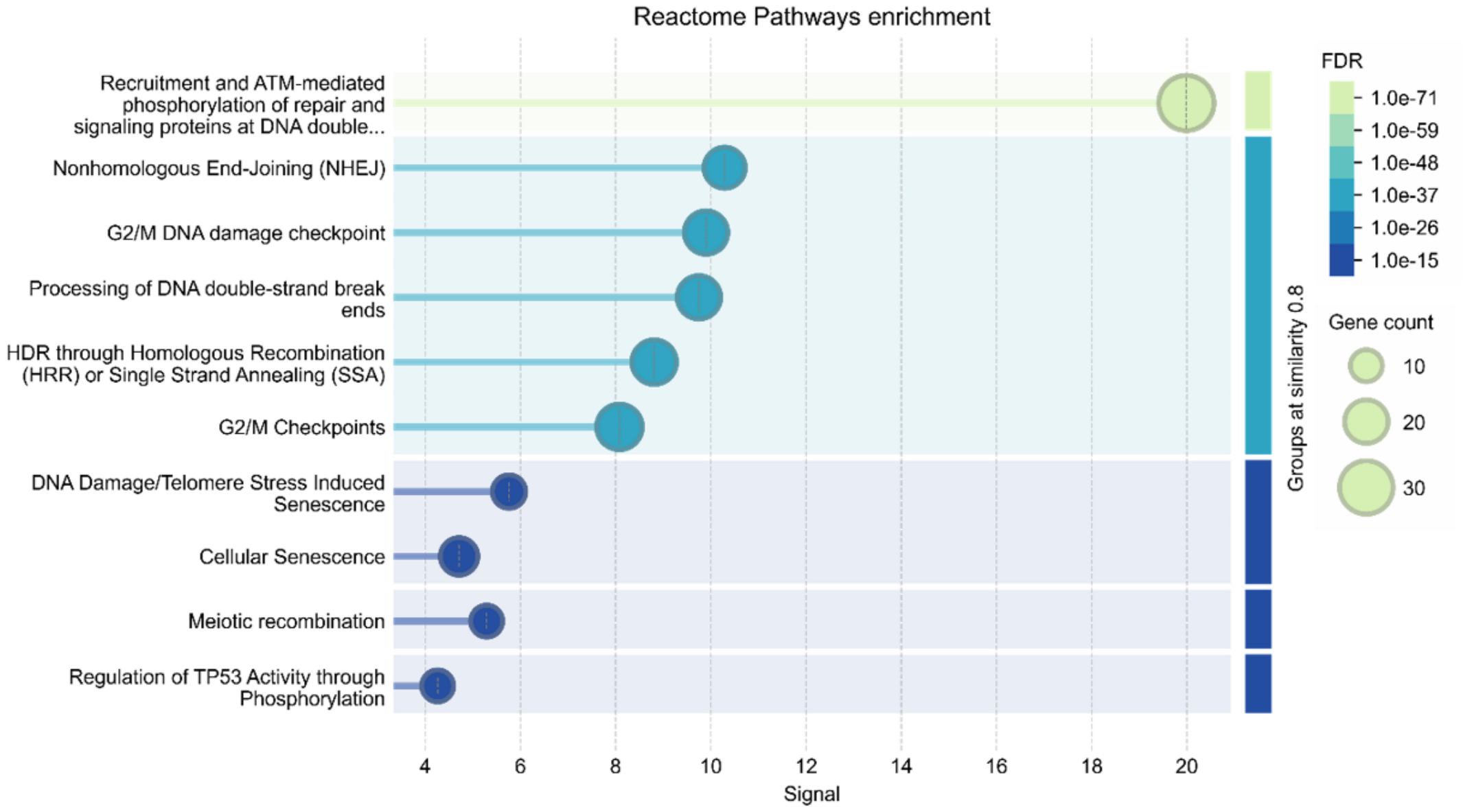
Reactome Pathways enrichment
Recruitment and ATM-mediated phosphorylation of repair and signaling proteins at DNA double...
Nonhomologous End-Joining (NHEJ)
G2/M DNA damage checkpoint
Processing of DNA double-strand break ends
HDR through Homologous Recombination (HRR) or Single Strand Annealing (SSA)
G2/M Checkpoints
DNA Damage/Telomere Stress Induced Senescence
Cellular Senescence
Meiotic recombination
Regulation of TP53 Activity through Phosphorylation
4
6
8
10
12
14
16
18
20
Signal
Groups at similarity 0.8
FDR
1.0e-71
1.0e-59
1.0e-48
1.0e-37
1.0e-26
1.0e-15
Gene count
10
20
30

(b)

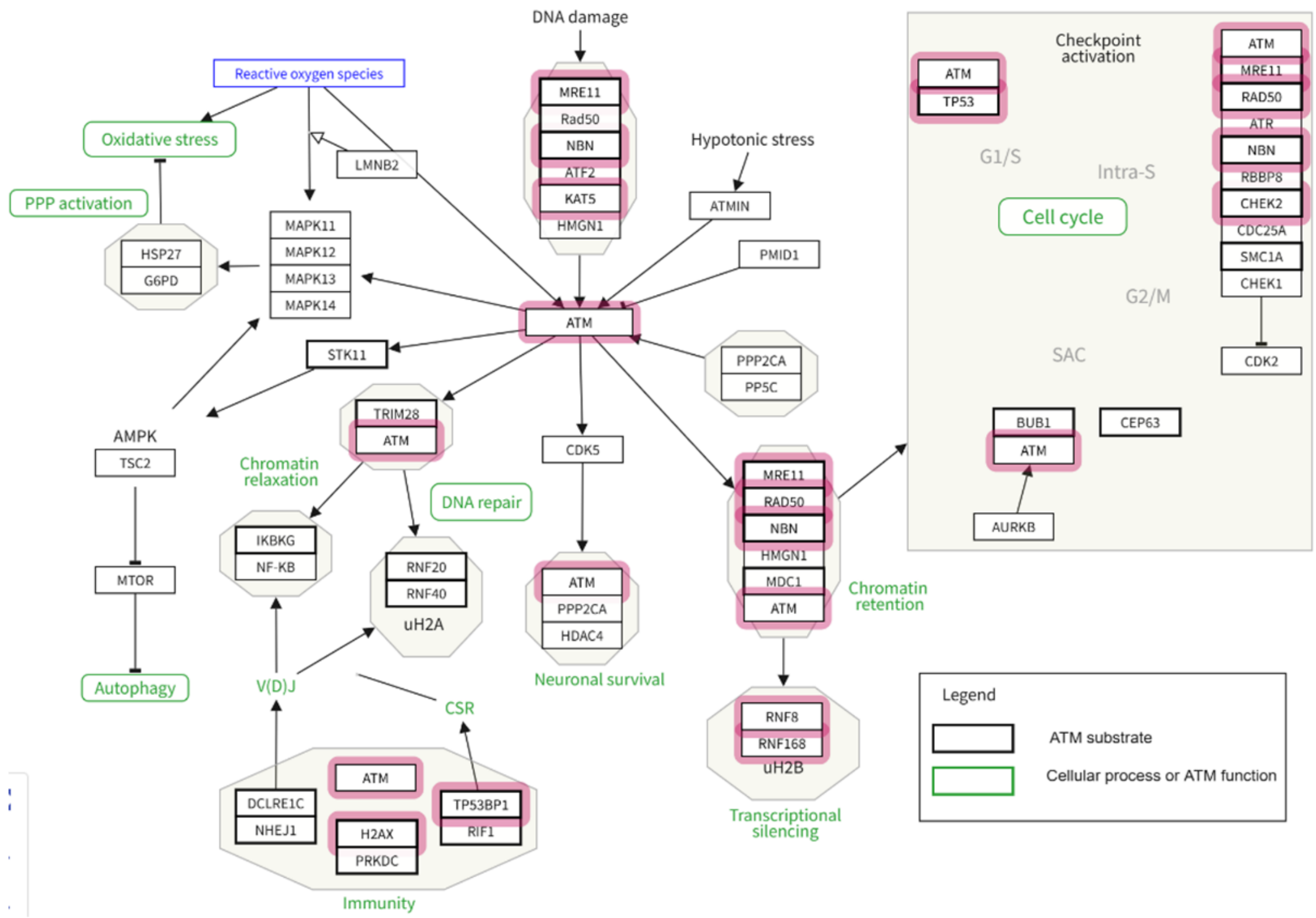
DNA damage
Reactive oxygen species
Oxidative stress
PPP activation
HSP27
G6PD
MAPK11
MAPK12
MAPK13
MAPK14
LMNB2
MRE11
Rad50
NBN
ATF2
KAT5
HMGN1
Hypotonic stress
ATMIN
PMID1
ATM
STK11
PPP2CA
PP5C
AMPK
TSC2
MTOR
Autophagy
TRIM28
ATM
Chromatin relaxation
IKBKG
NF-KB
RNF20
RNF40
uH2A
DNA repair
CDK5
ATM
PPP2CA
HDAC4
Neuronal survival
MRE11
RAD50
NBN
HMGN1
MDC1
ATM
Chromatin retention
RNF8
RNF168
uH2B
Transcriptional silencing
V(D)J
CSR
ATM
DCLRE1C
NHEJ1
TP53BP1
RIF1
H2AX
PRKDC
Immunity
Checkpoint activation
ATM
TP53
G1/S
Intra-S
Cell cycle
G2/M
SAC
ATM
MRE11
RAD50
ATR
NBN
RBBP8
CHEK2
CDC25A
SMC1A
CHEK1
CDK2
BUB1
ATM
CEP63
AURKB
Legend
ATM substrate
Cellular process or ATM function

(a)

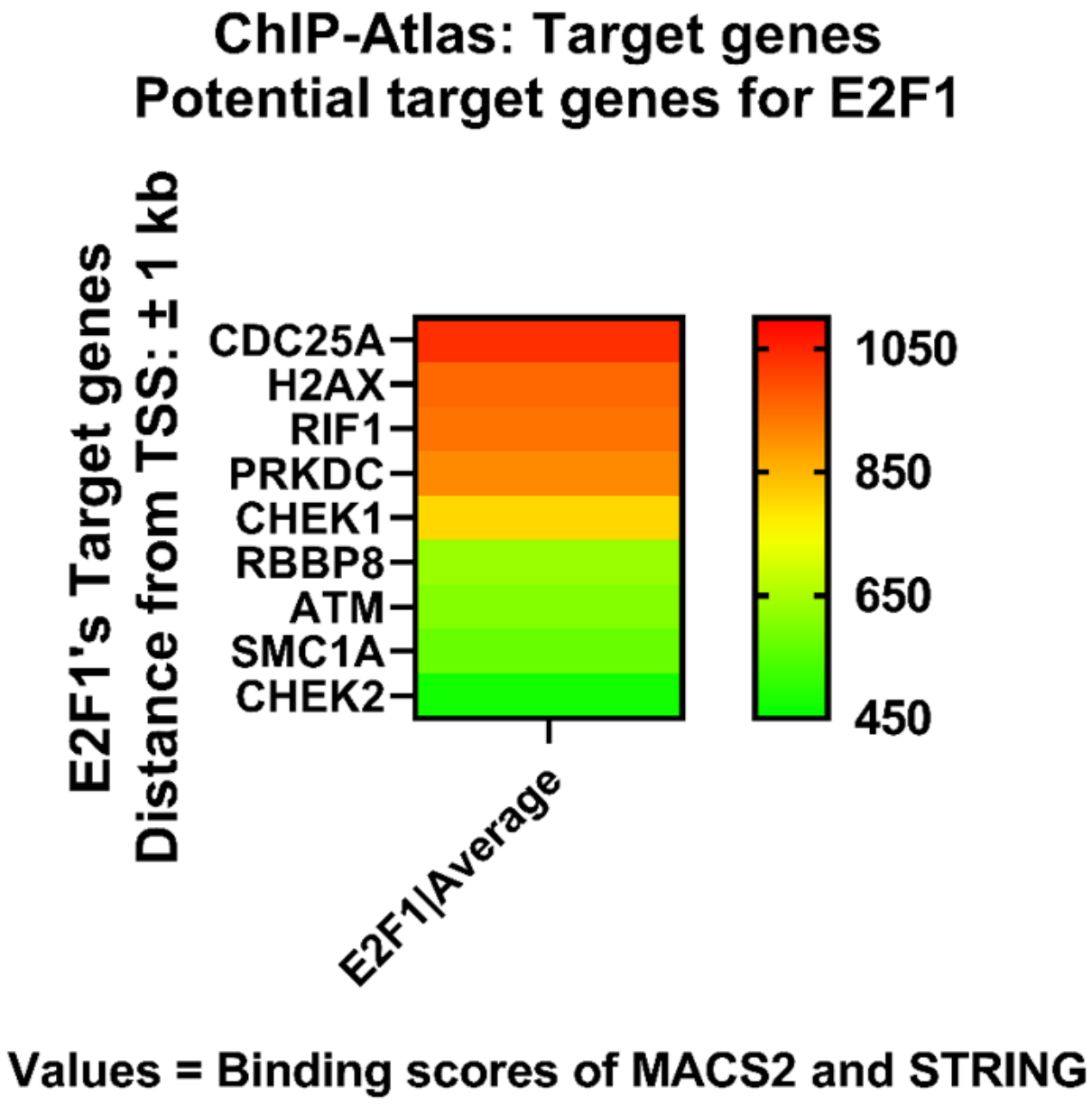
ChIP-Atlas: Target genes
Potential target genes for E2F1
E2F1's Target genes
Distance from TSS: ±1 kb
CDC25A
H2AX
RIF1
PRKDC
CHEK1
RBBP8
ATM
SMC1A
CHEK2
E2F1|Average
1050
850
650
450
Values = Binding scores of MACS2 and STRING

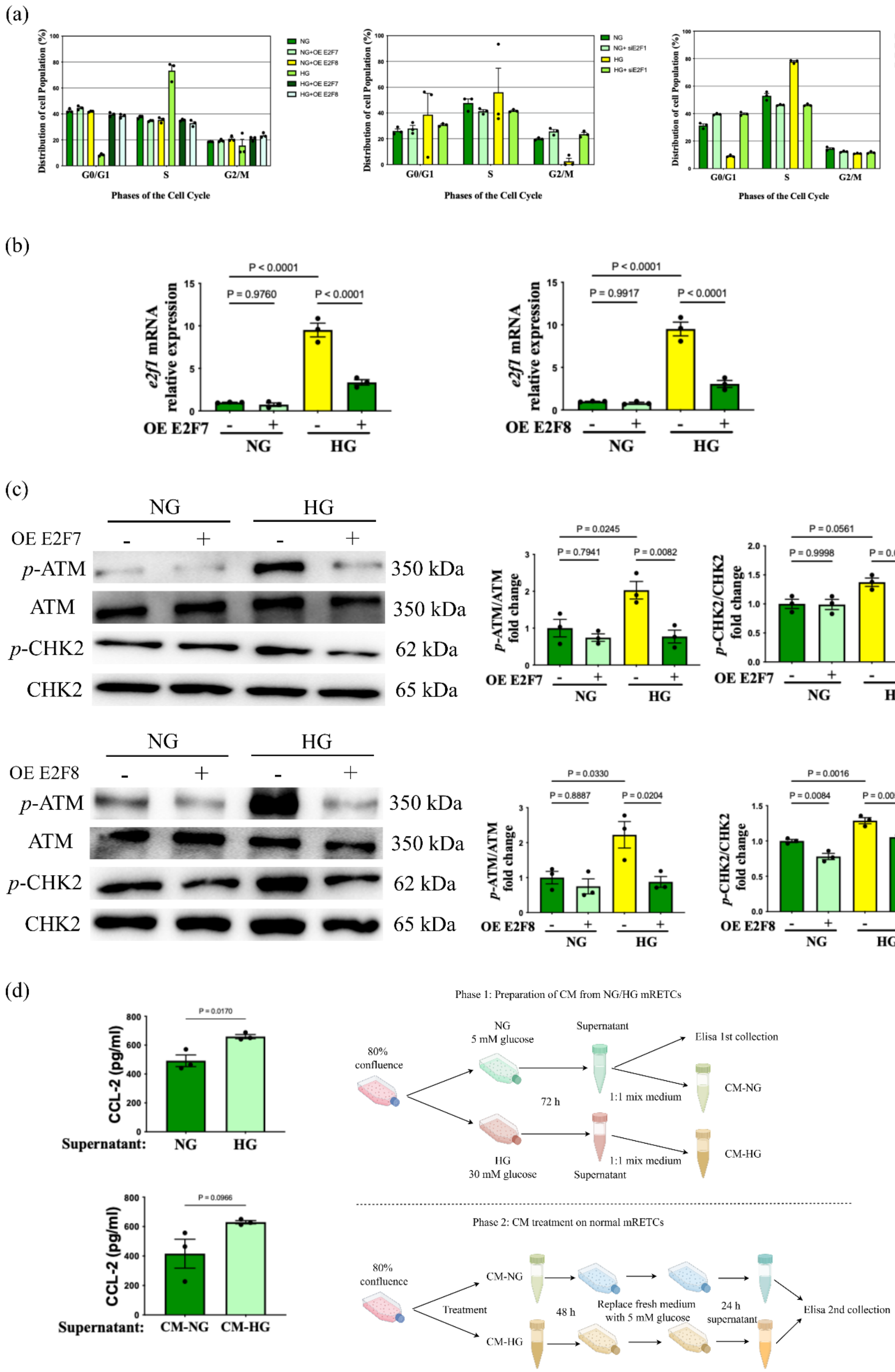
(a)
Distribution of cell Population (%)
NG
NG+OE E2F7
NG+OE E2F8
HG
HG+OE E2F7
HG+OE E2F8
G0/G1
S
G2/M
Phases of the Cell Cycle
NG
NG+ siE2F1
HG
HG+ siE2F1
NG
NG+Res
HG
HG+Res
(b)
e2f1 mRNA relative expression
P < 0.0001
P = 0.9760
P < 0.0001
OE E2F7
NG
HG
P < 0.0001
P = 0.9917
P < 0.0001
OE E2F8
(c)
NG
HG
OE E2F7
p-ATM
ATM
p-CHK2
CHK2
350 kDa
350 kDa
62 kDa
65 kDa
p-ATM/ATM fold change
P = 0.0245
P = 0.7941
P = 0.0082
p-CHK2/CHK2 fold change
P = 0.0561
P = 0.9998
P = 0.0529
OE E2F8
P = 0.0330
P = 0.8887
P = 0.0204
P = 0.0016
P = 0.0084
P = 0.0059
(d)
CCL-2 (pg/ml)
P = 0.0170
Supernatant:
NG
HG
P = 0.0966
CM-NG
CM-HG
Phase 1: Preparation of CM from NG/HG mRETCs
80% confluence
NG 5 mM glucose
Supernatant
Elisa 1st collection
72 h
1:1 mix medium
CM-NG
HG 30 mM glucose
Supernatant
1:1 mix medium
CM-HG
Phase 2: CM treatment on normal mRETCs
80% confluence
CM-NG
Treatment
48 h
Replace fresh medium with 5 mM glucose
24 h supernatant
Elisa 2nd collection
CM-HG

(a)

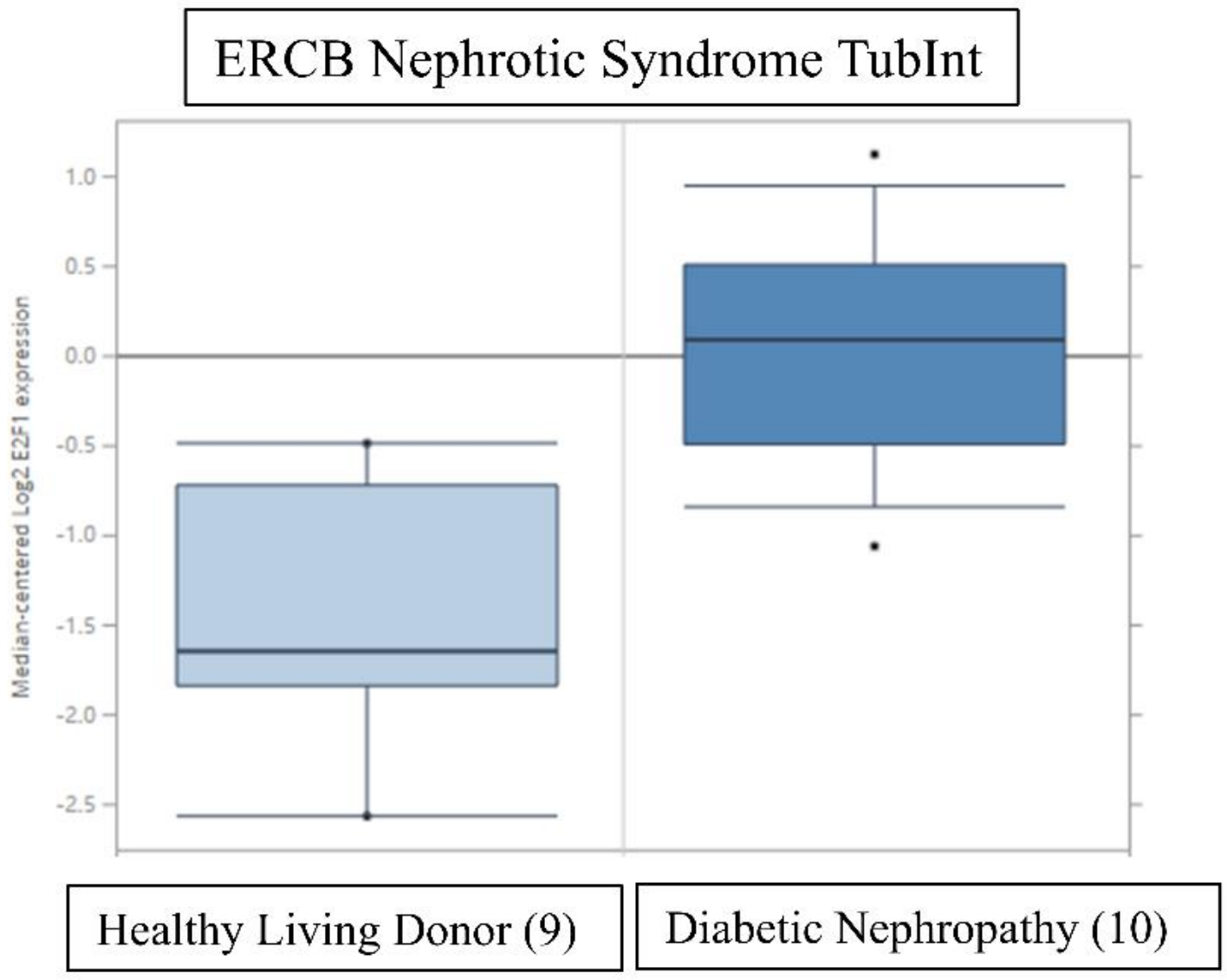
ERCB Nephrotic Syndrome TubInt
Median-centered Log2 E2F1 expression
1.0
0.5
0.0
-0.5
-1.0
-1.5
-2.0
-2.5
Healthy Living Donor (9)
Diabetic Nephropathy (10)

(b)

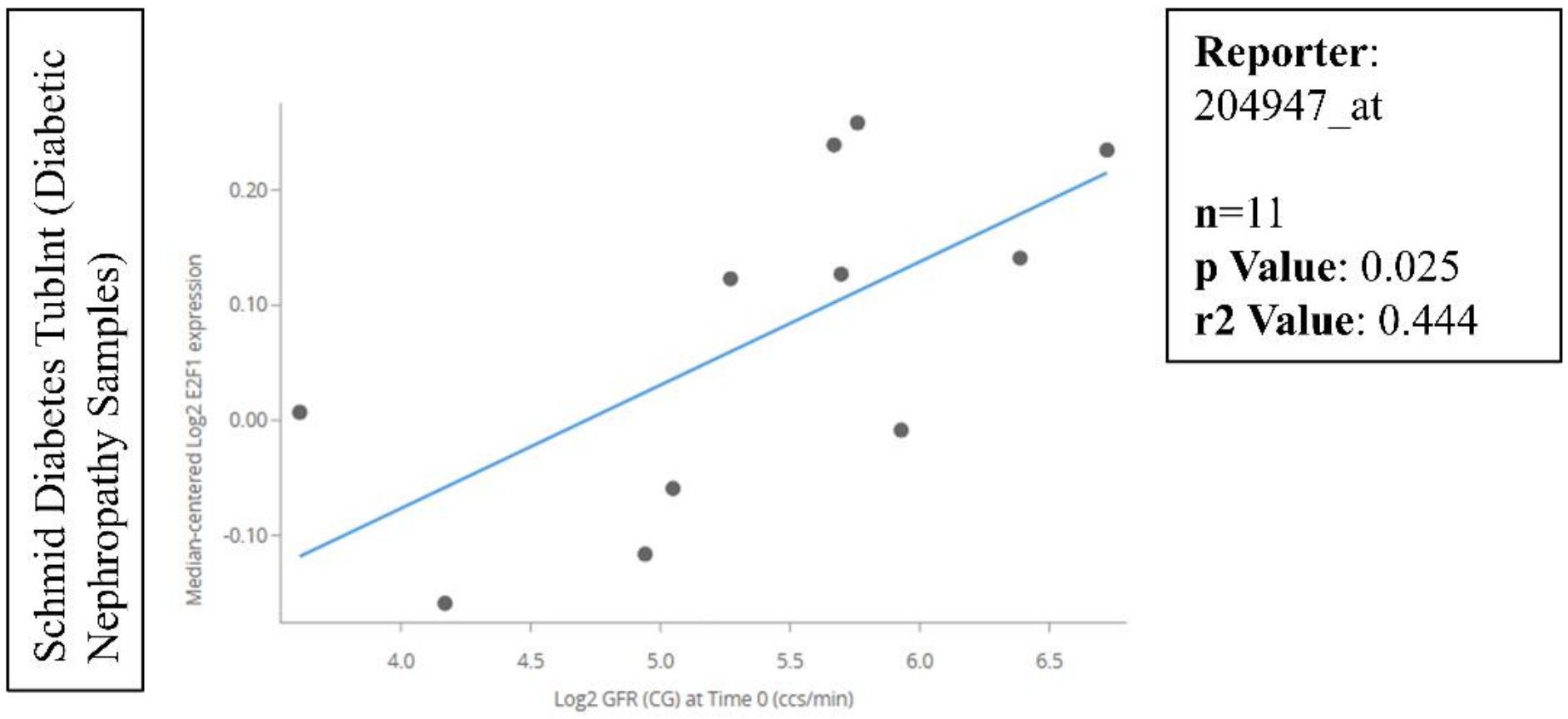
Schmid Diabetes TubInt (Diabetic Nephropathy Samples)
Median-centered Log2 E2F1 expression
0.20
0.10
0.00
-0.10
4.0
4.5
5.0
5.5
6.0
6.5
Log2 GFR (CG) at Time 0 (ccs/min)
Reporter: 204947_at
n=11
p Value: 0.025
r2 Value: 0.444

(c)

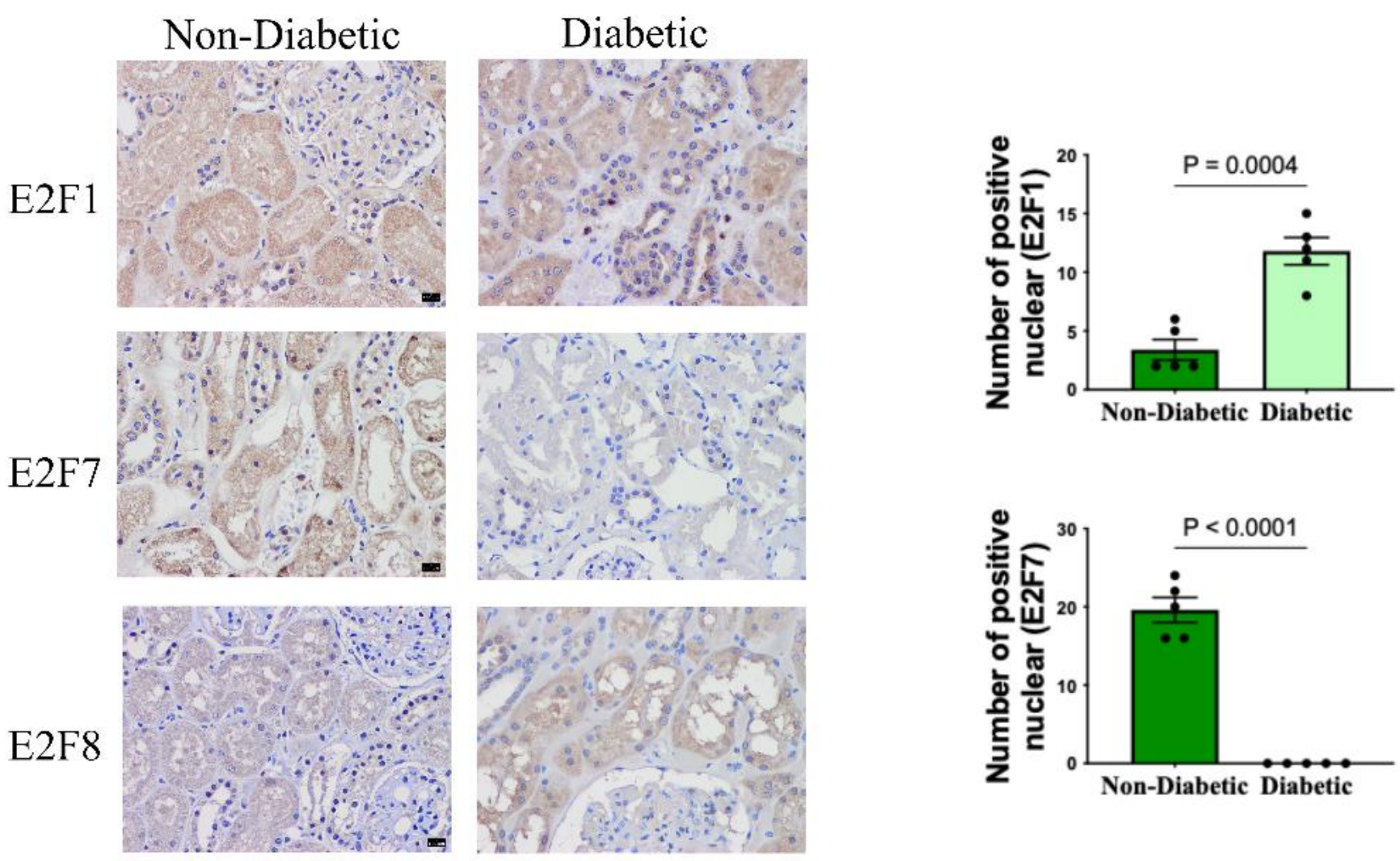
Non-Diabetic
Diabetic
E2F1
E2F7
E2F8
Number of positive nuclear (E2F1)
P = 0.0004
20
15
10
5
0
Non-Diabetic
Diabetic
Number of positive nuclear (E2F7)
P < 0.0001
30
20
10
0
Non-Diabetic
Diabetic

Original uncut blots

Figure 1c

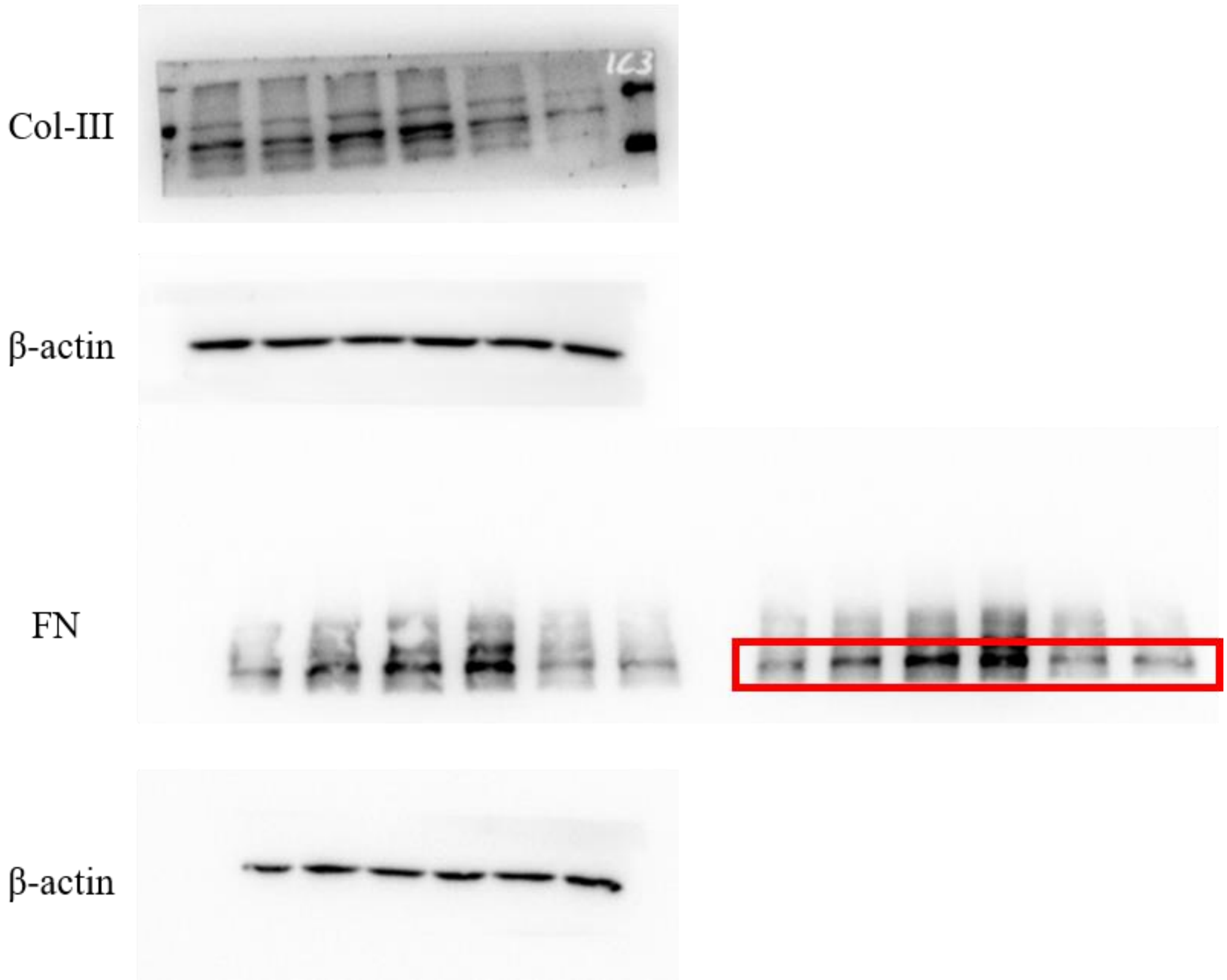

Figure 2b

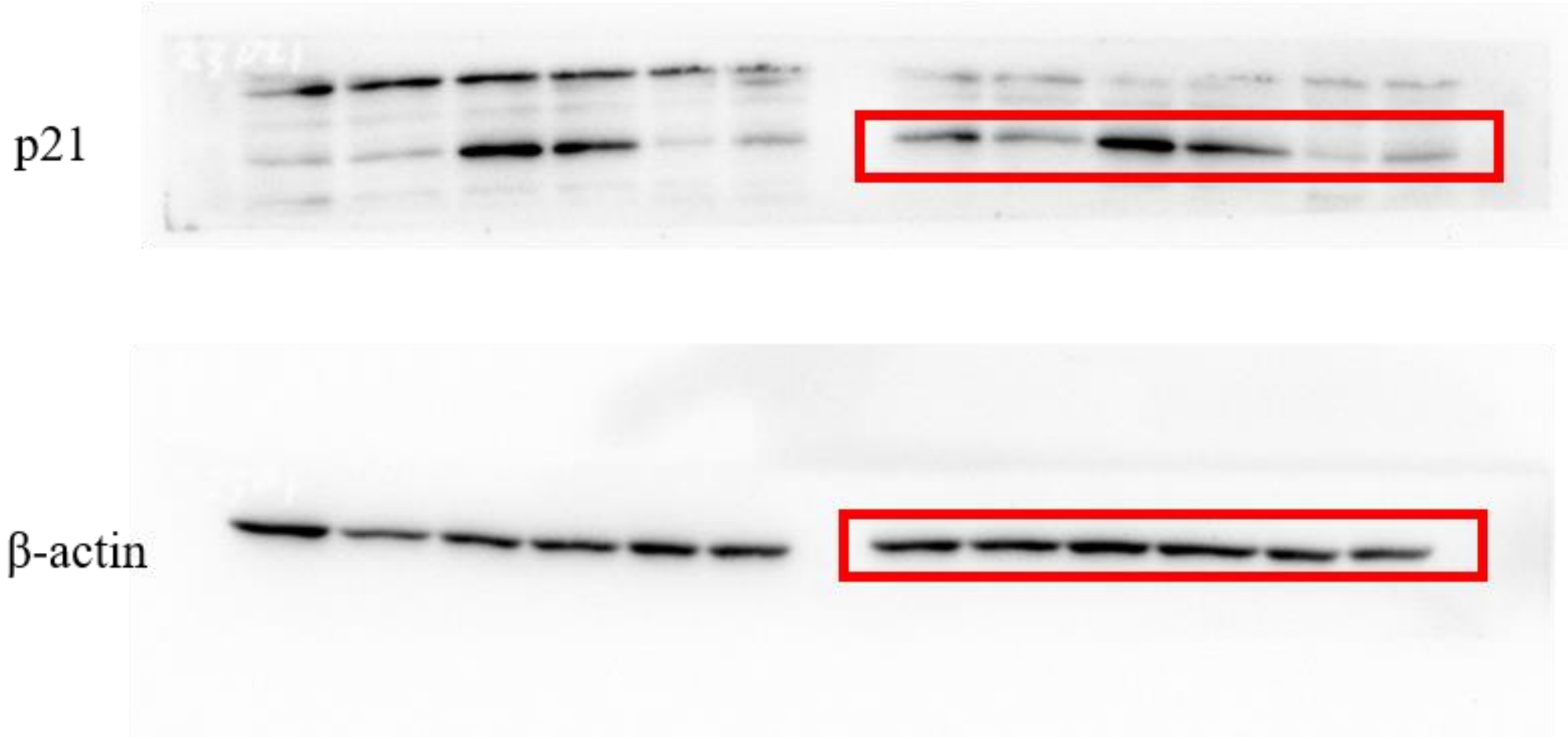

Figure S2d

Vcam-1

β-actin

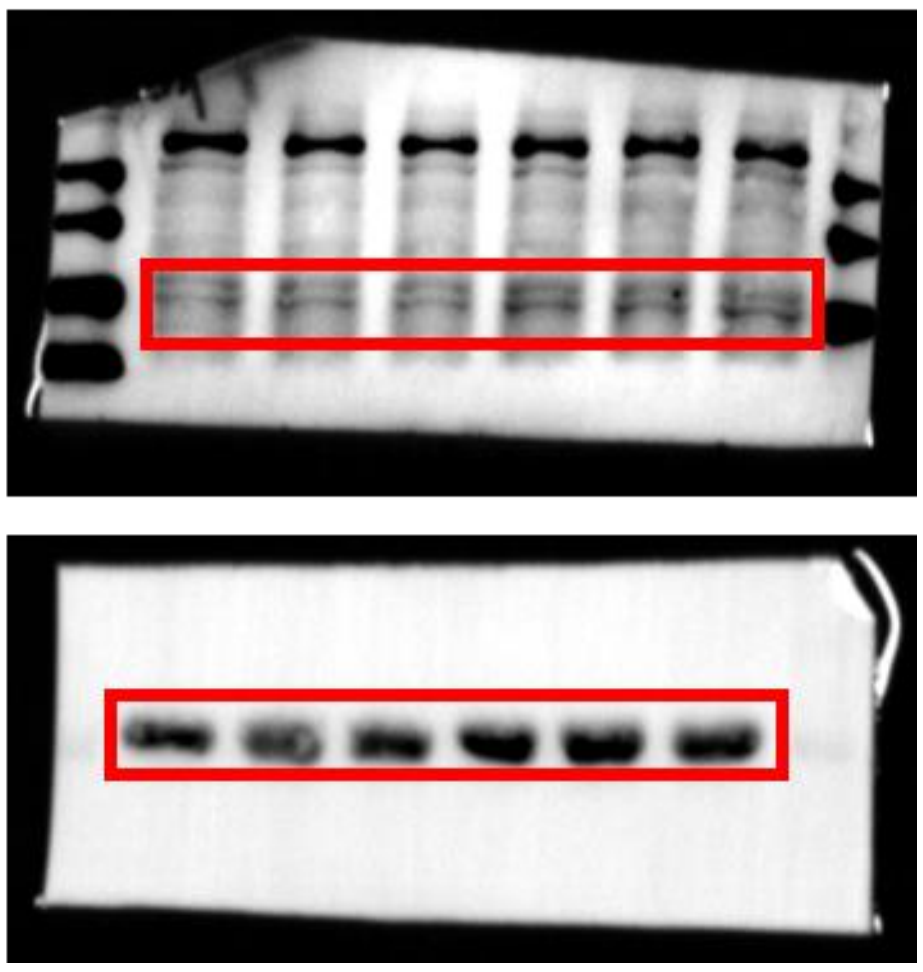

Figure 3c

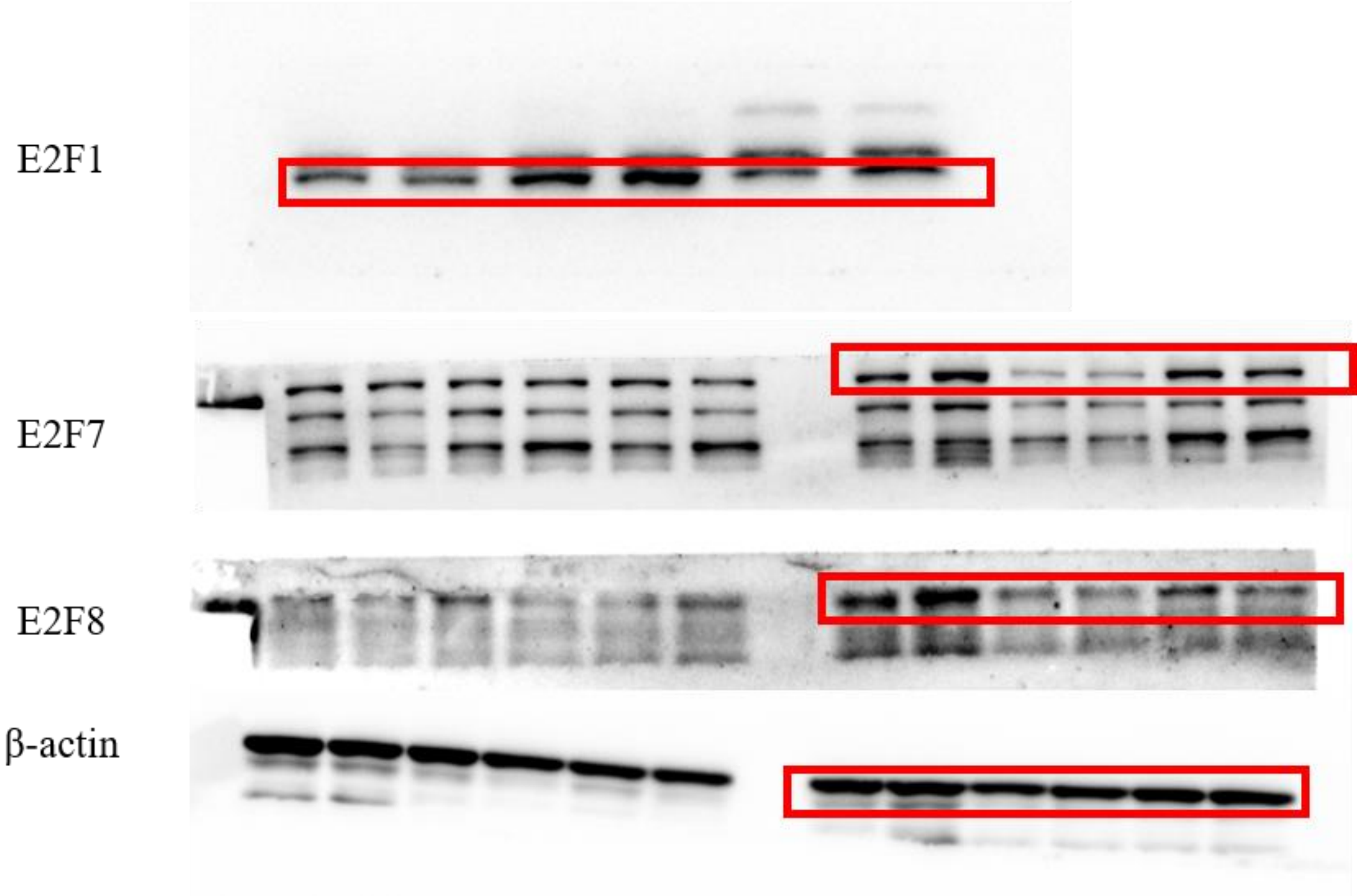

Figure S3b left panel

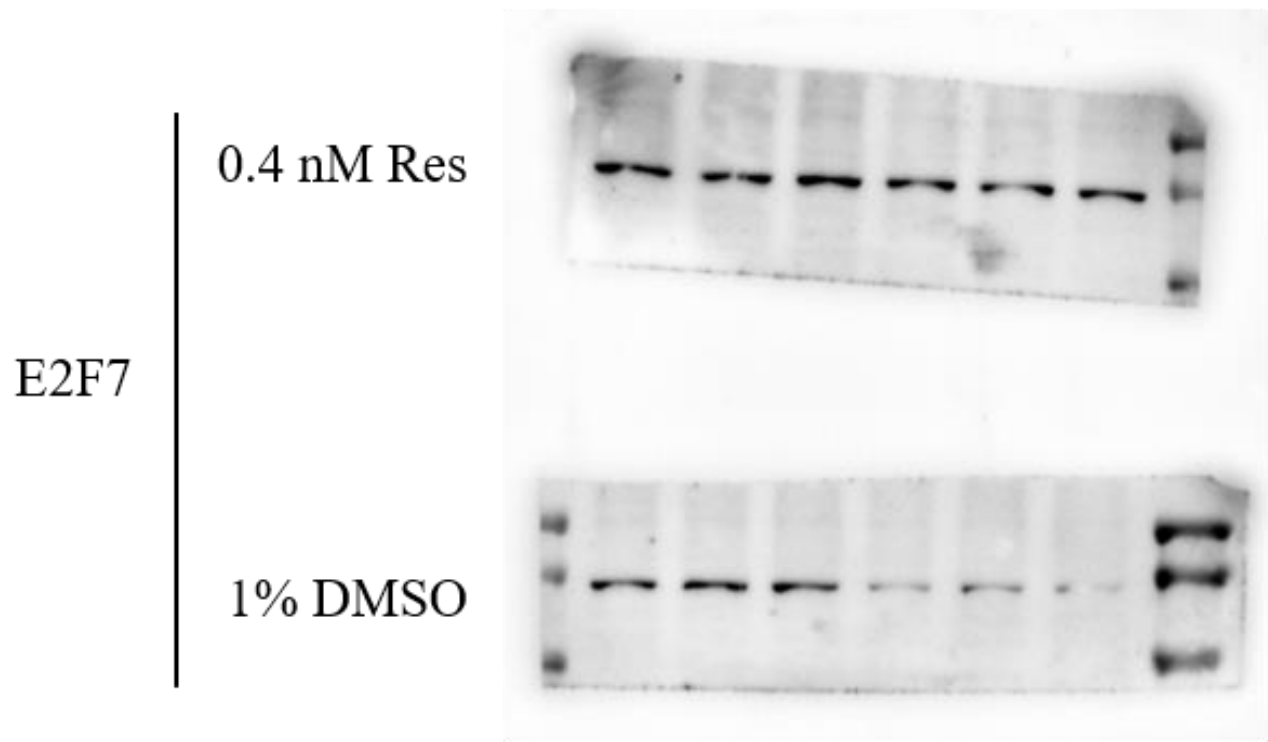

Figure S3b right panel

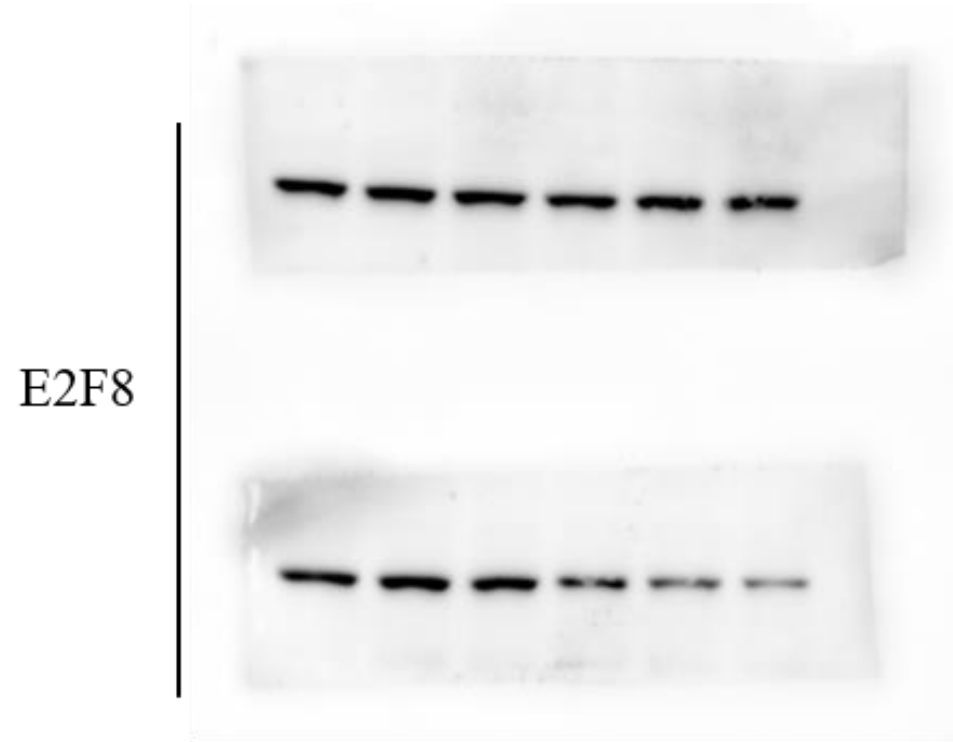

Figure S3c

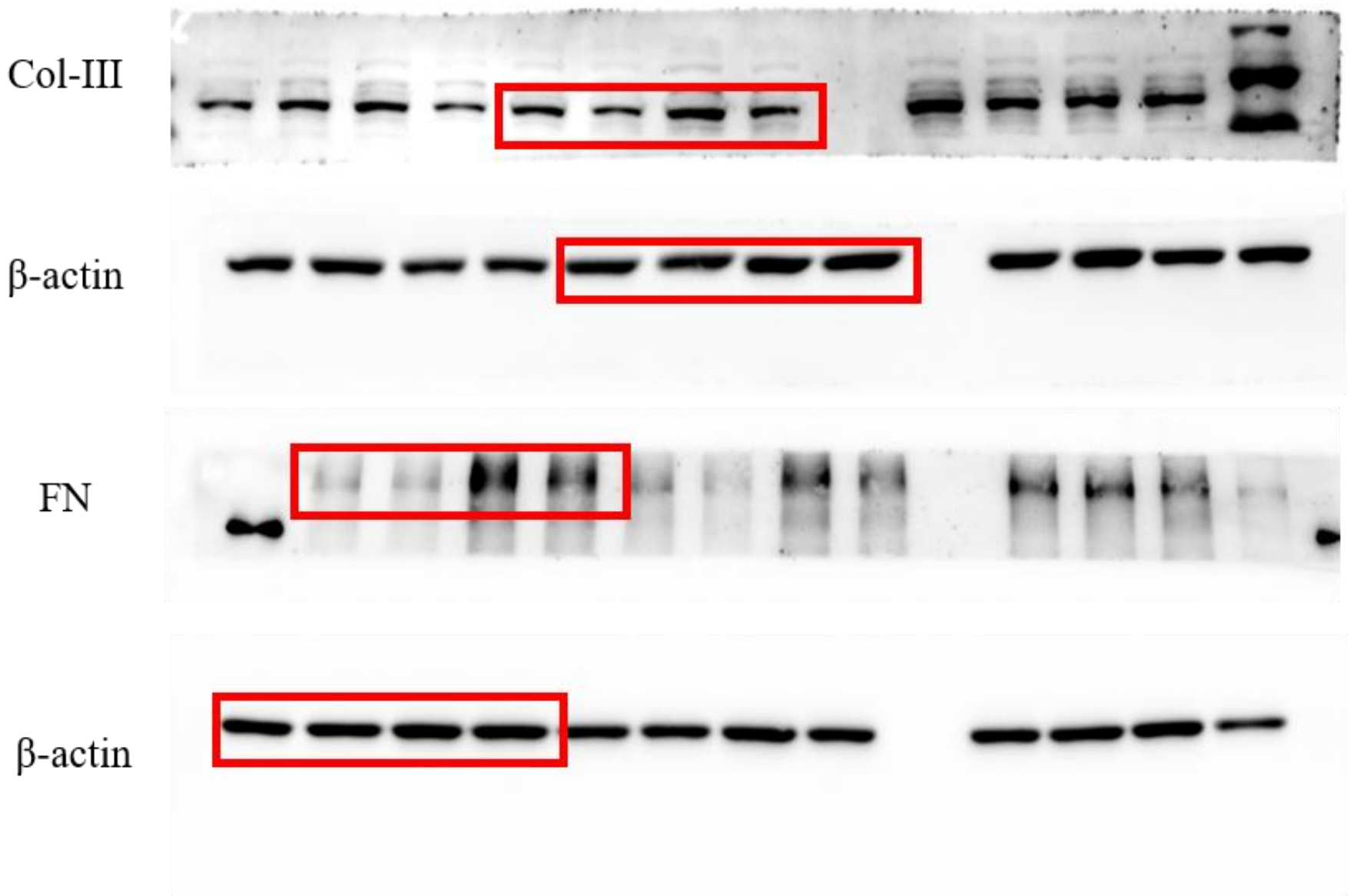

Figure S3d

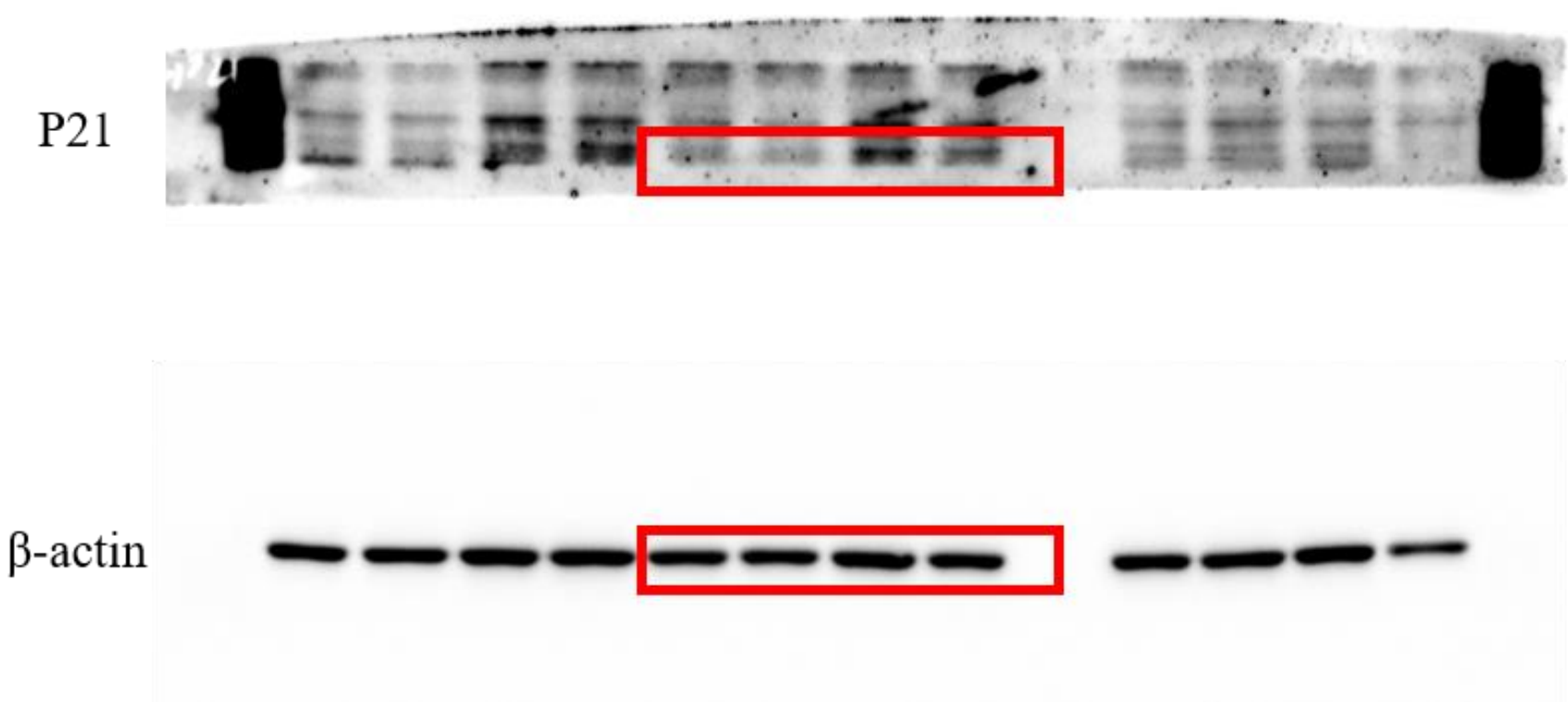

Figure 4a

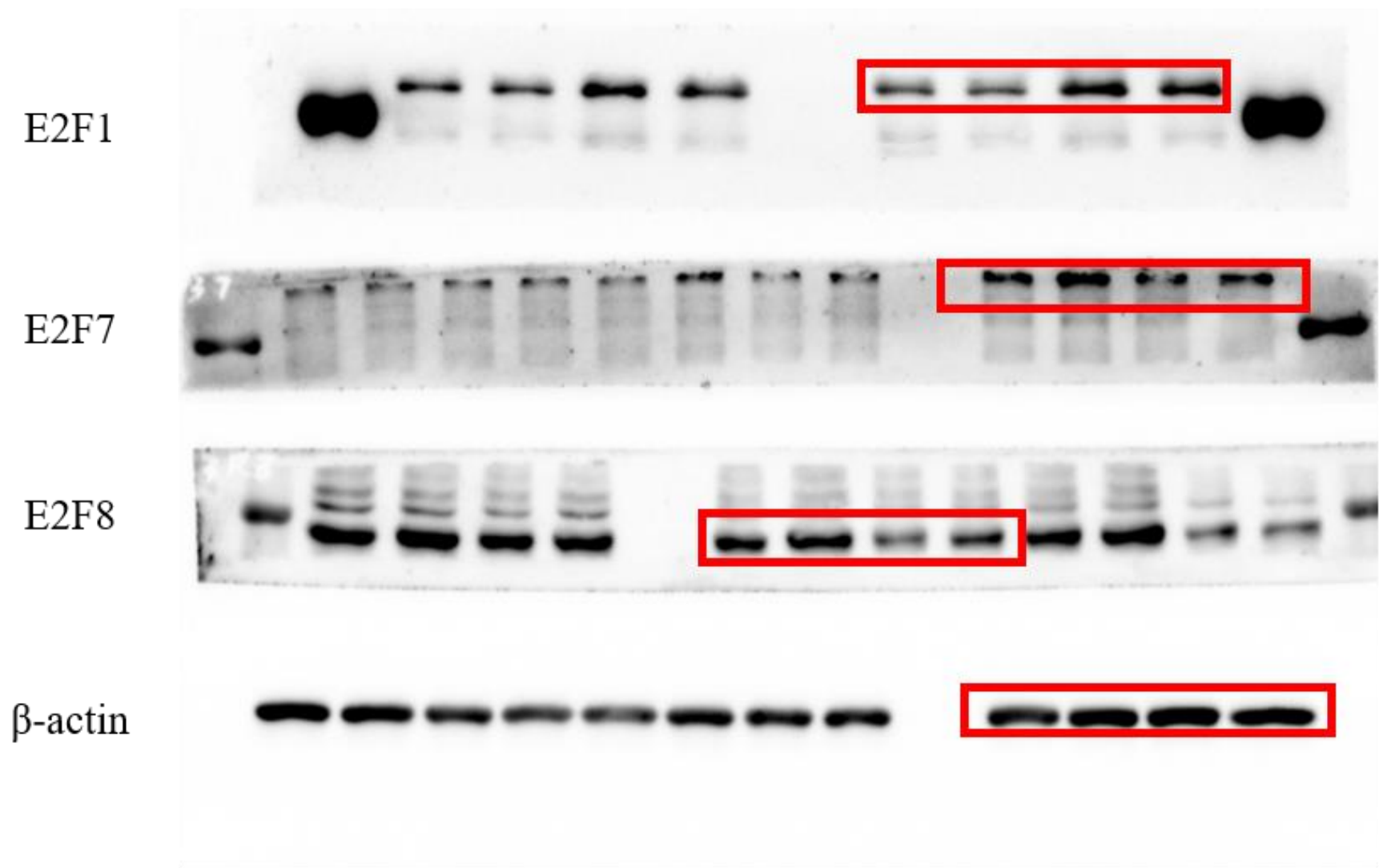

Figure 4b left panel

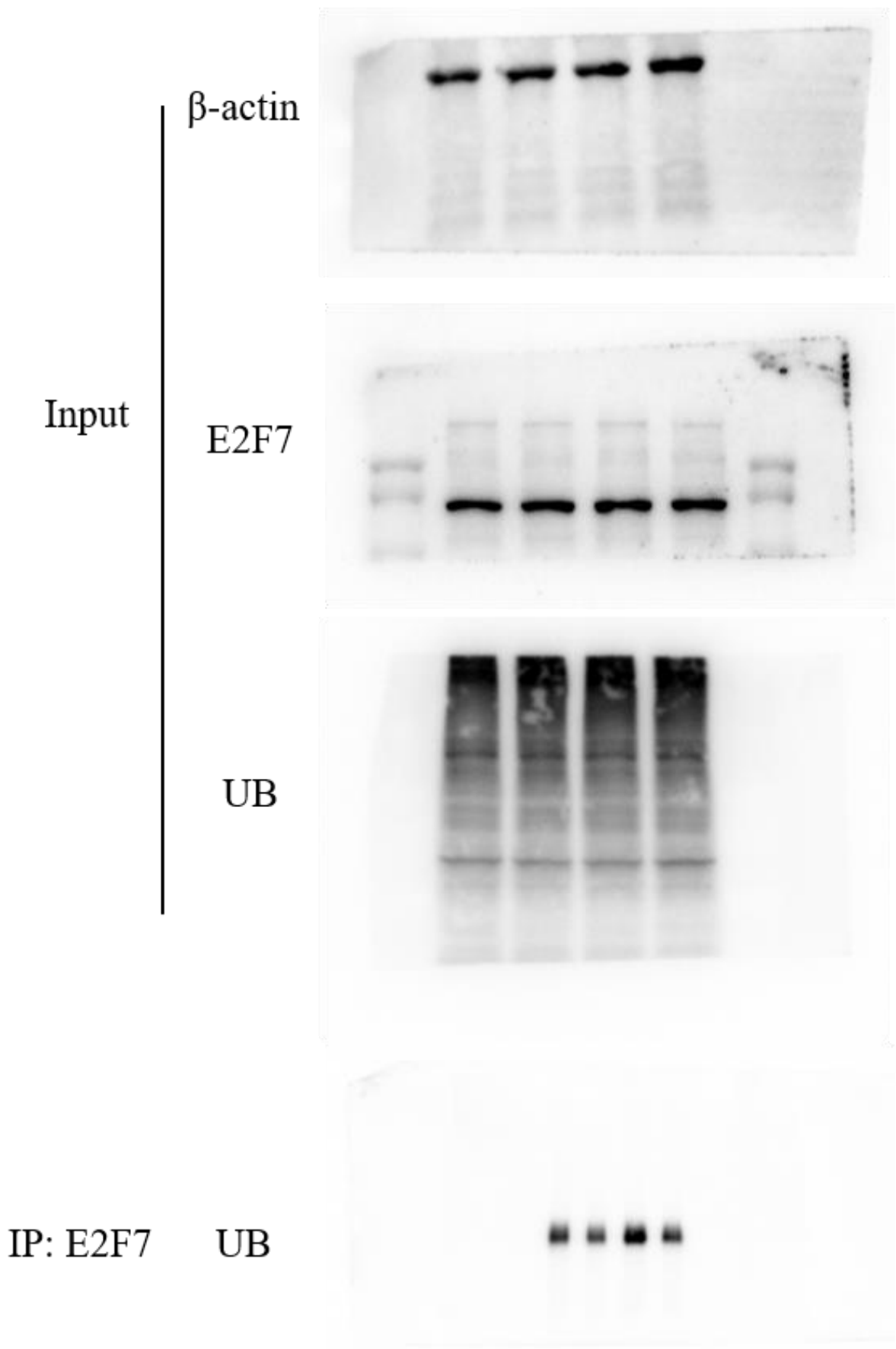

Figure 4e right panel

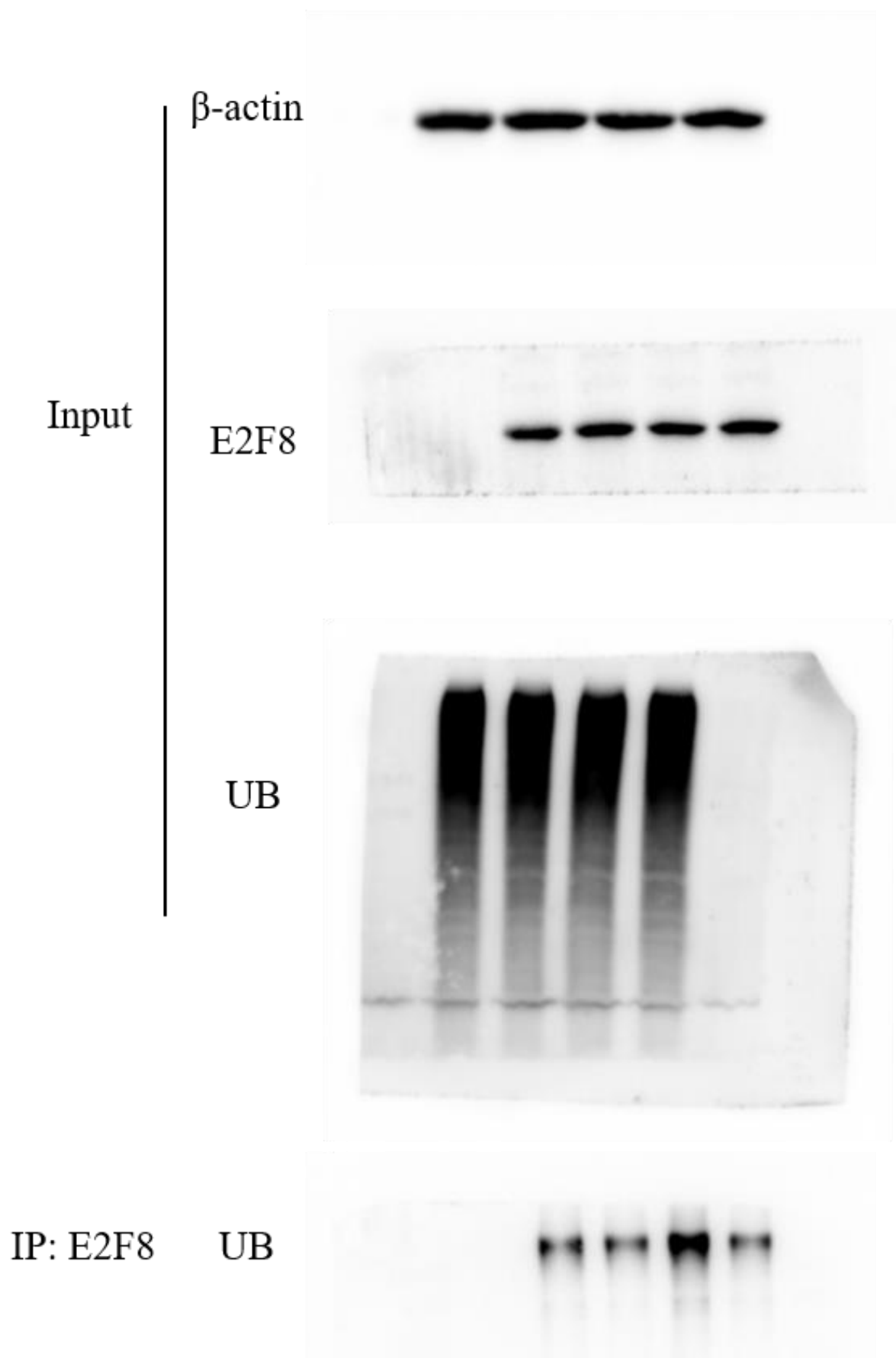

Figure 5c

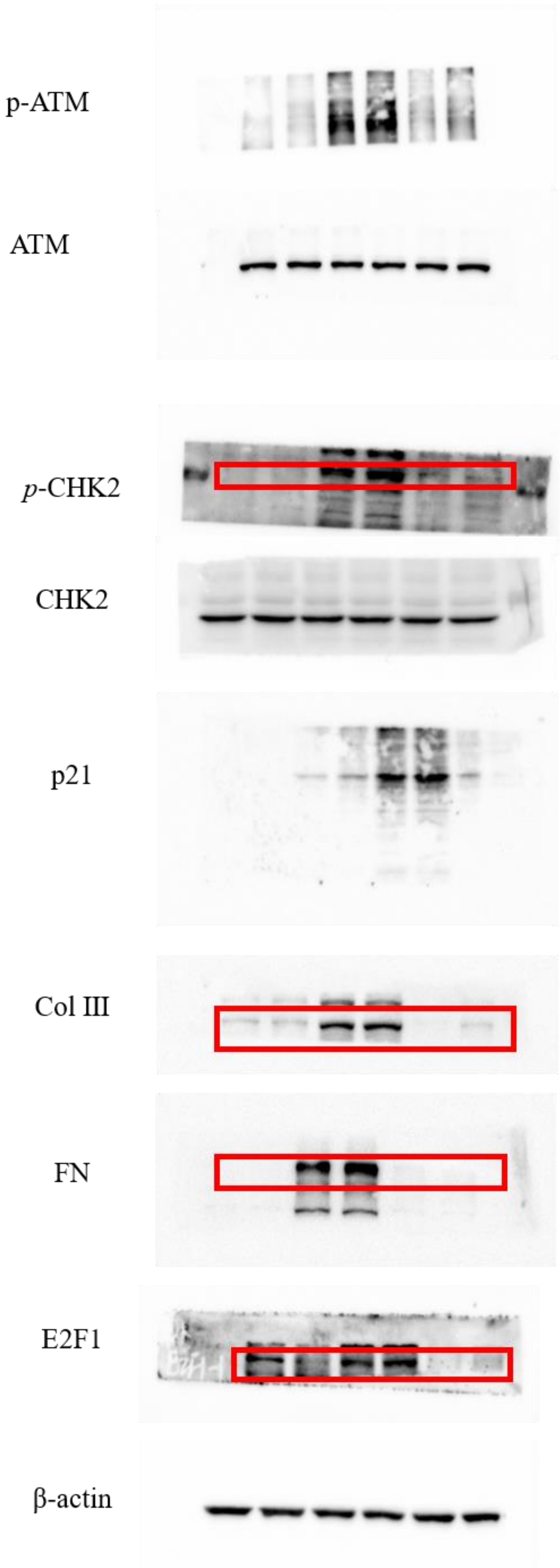

Figure 6b

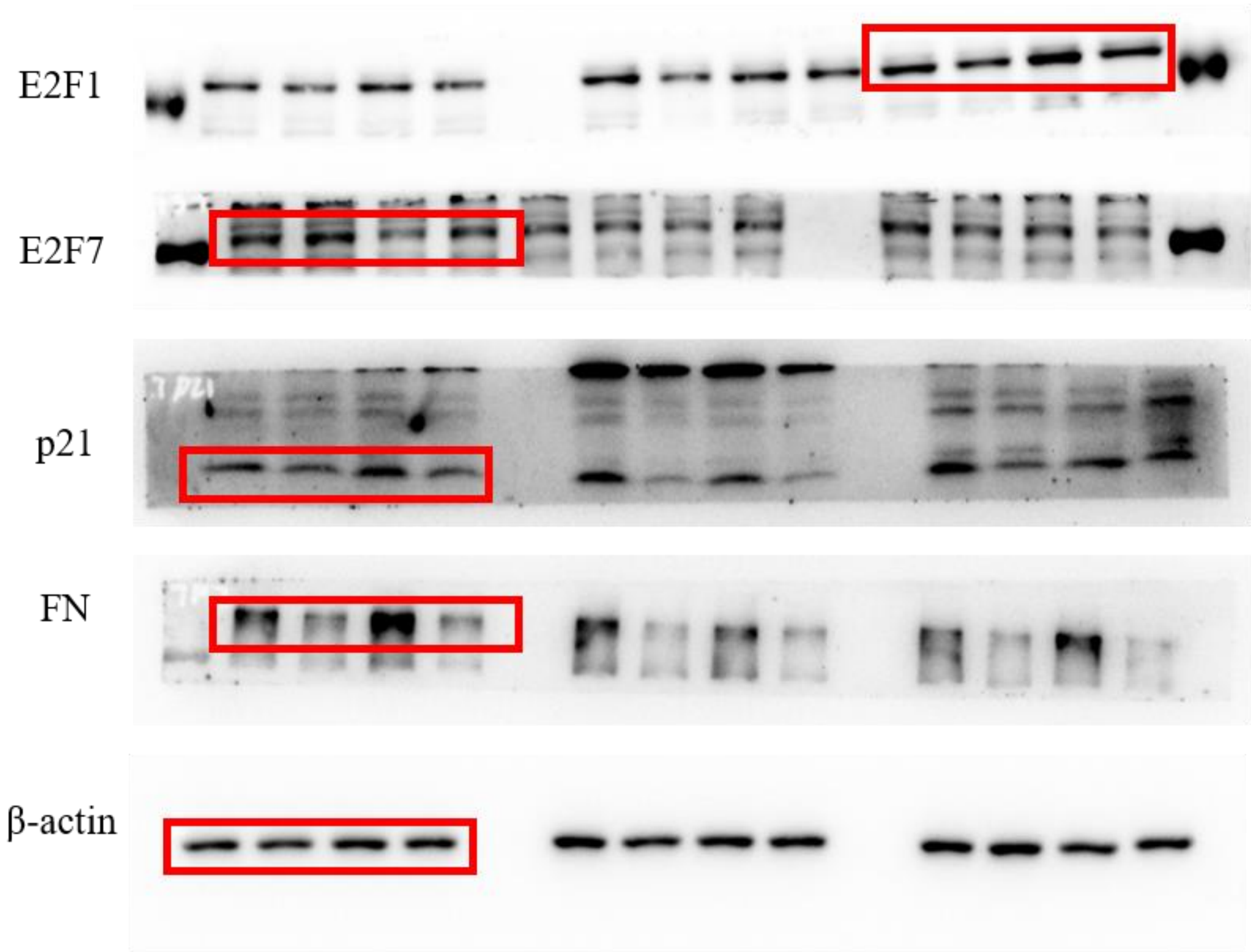

Figure 6d

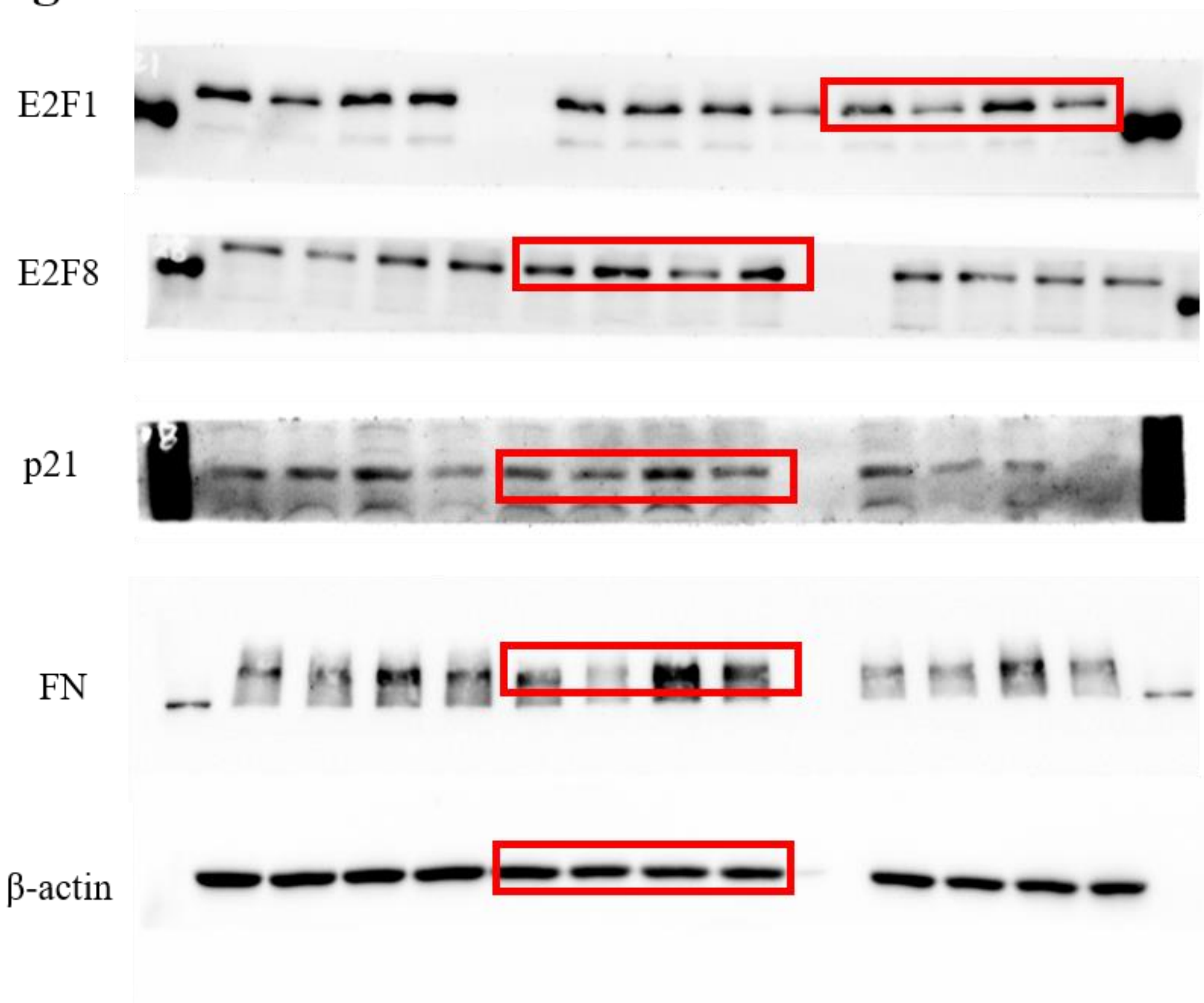

Figure S6c top panle

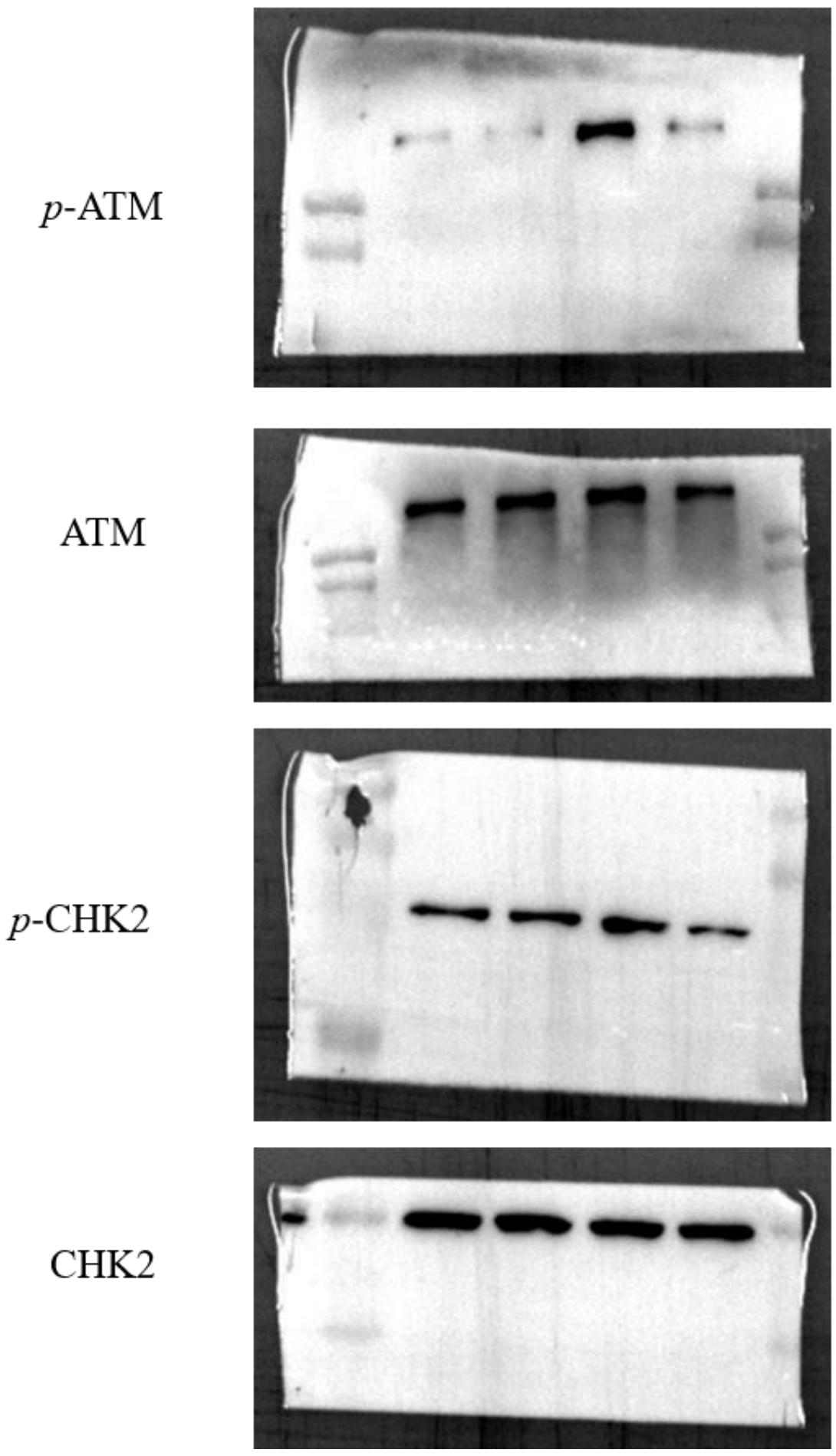

Figure S6c bottom panle

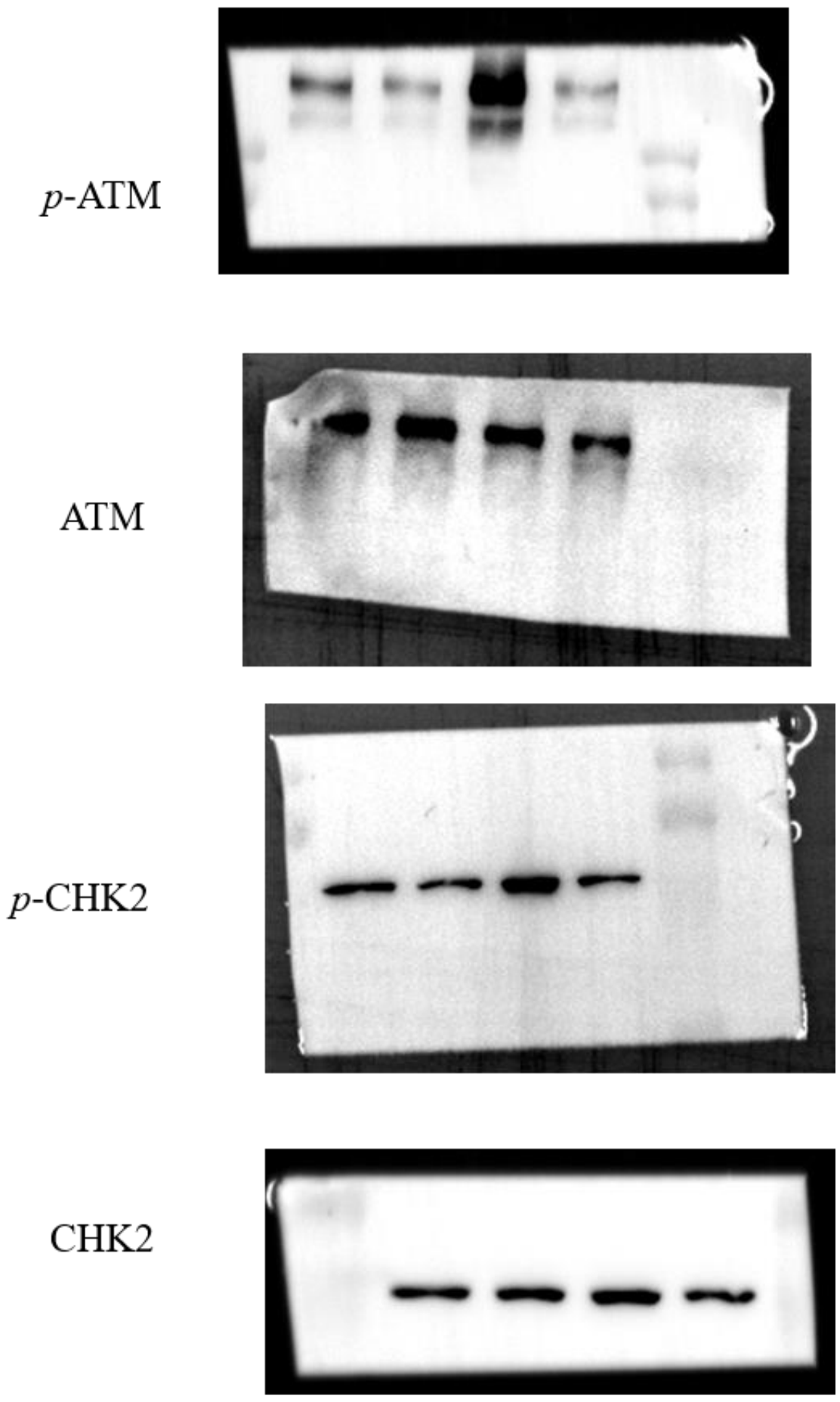

Figure 7a

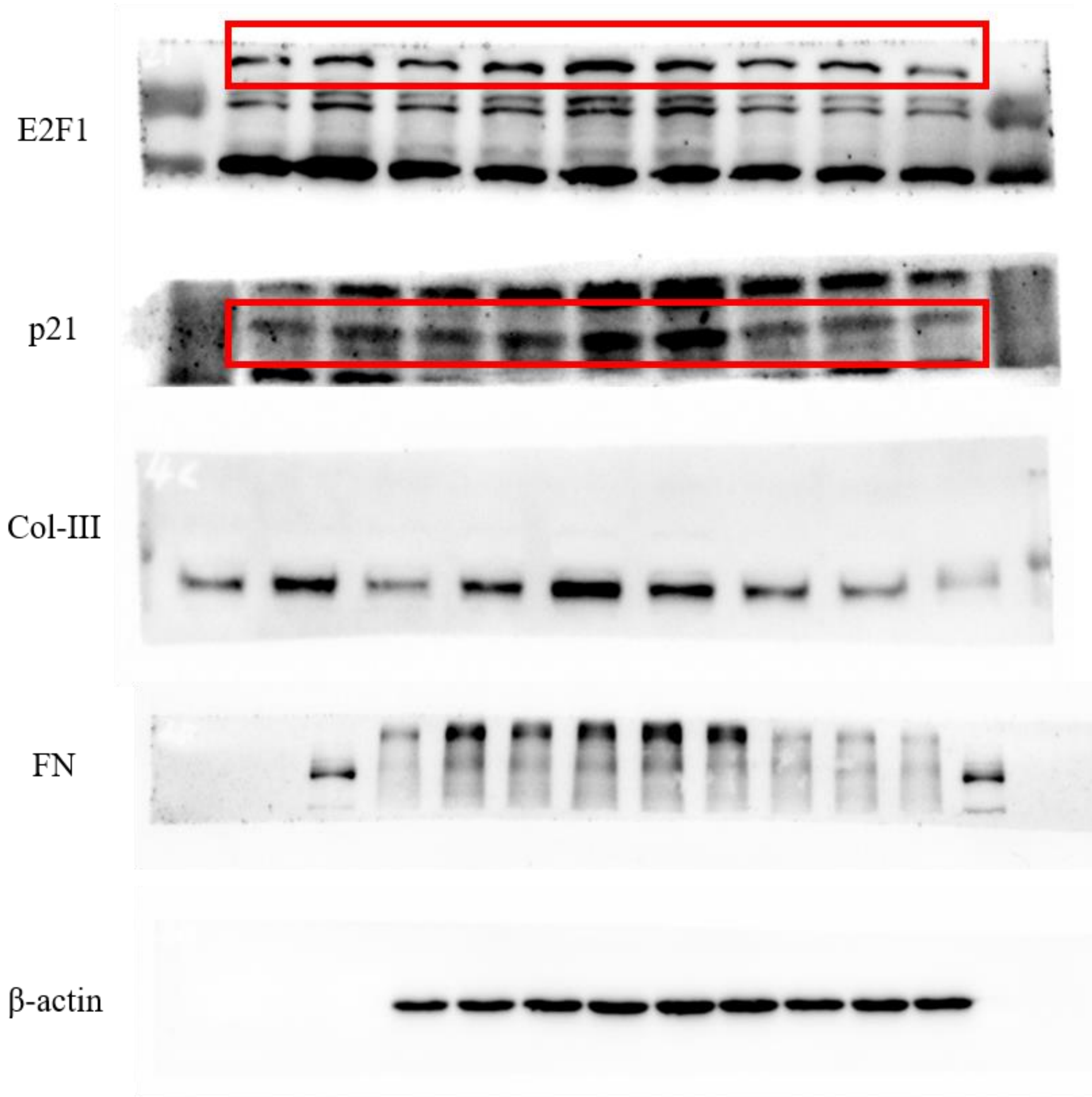

Figure 7b

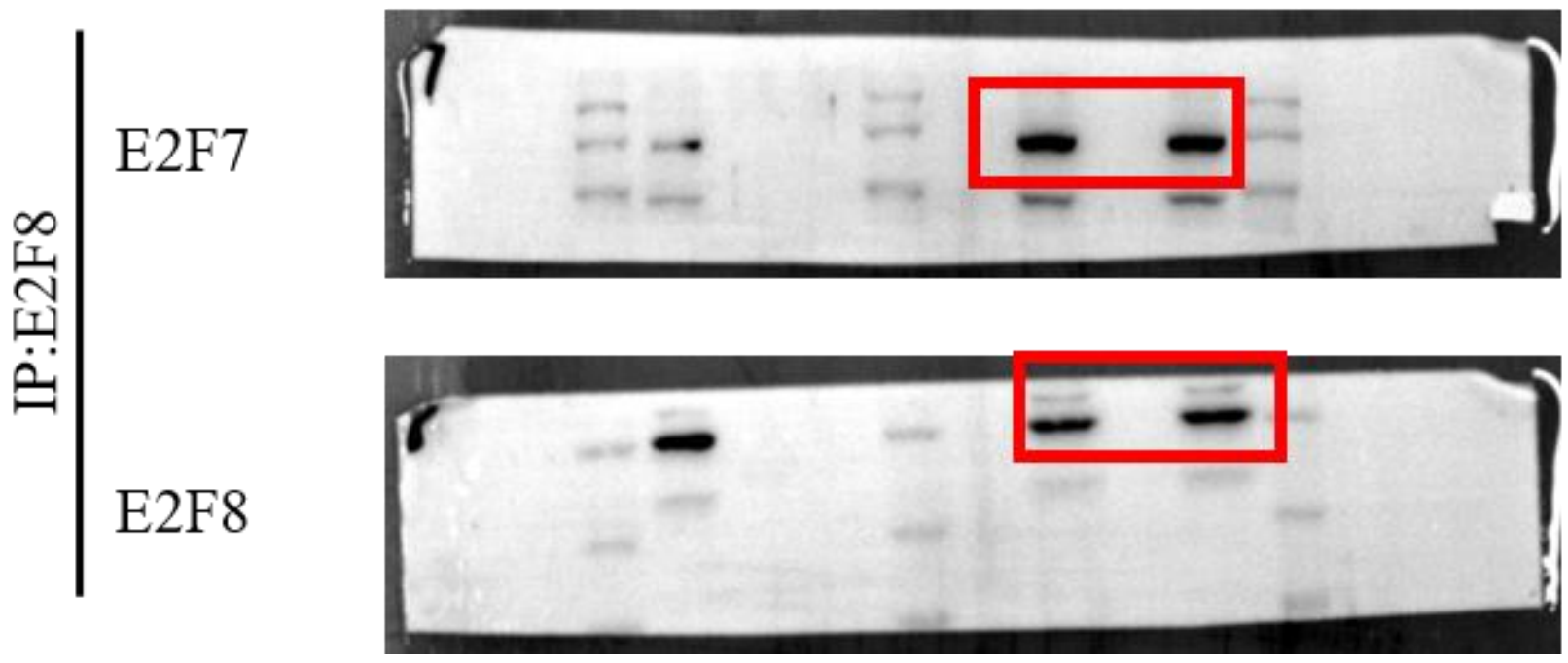

Supplement: Supplementary file 2 [file 42003_2026_11334_MOESM2_ESM.pdf]
