# Supplementary material for: E2F7 and E2F8 attenuate diabetic kidney disease by suppressing E2F1-induced tubular senescence
Source: Commun Biol. 2026 Nov 13;9:1526. doi: 10.1038/s42003-026-11334-1 (PMC13768344; doi:10.1038/s42003-026-11334-1)
Supplement: Supplementary file 3 [file 42003_2026_11334_MOESM3_ESM.pdf]

## Description of Additional Supplementary Files

**Title:** Supplementary Table 1

**Description:** Primer list of qPCR and CHIP-qPCR (*Mus musculus*) using in current study.

**Title:** Supplementary Table 2

**Description:** Primary antibody list used in present study.

**Title:** Supplementary Table 3

**Description:** The 35 annotated genes interacted with resveratrol. The pathway Recruitment and ATM-mediated phosphorylation of repair and signaling proteins at DNA double strand breaks (REACT:R-HSA-5693565) analyzed by CTD database.

**Title:** Supplementary Figure 1

**Description:** Serum biochemical analysis of *db/m* control and *db/db* mice and representative image of kidney sections stained with HE staining, Sirius Red, and Periodic acid-Schiff.

**Title:** Supplementary Figure 2

**Description:** Cell viability of mRTECs treated with resveratrol and live cell quantification of mRTECs following exposure to the indicated glucose concentrations. Relative nuclear expression of p21 and Vcam-1 expression levels in mRTECs in response to high glucose conditions

**Title:** Supplementary Figure 3

**Description:** Molecular docking of resveratrol and the E2F7or E2F8 proteins. Cellular thermal shift assay CETSA of E2F7 and E2F8 upon resveratrol binding. Representative

immunoblots and corresponding densitometric quantification of Collagen-III and Fibronectin expression in mRTECs and representative immunoblots and densitometric analysis of p21 protein expression in mRTECs

**Title:** Supplementary Figure 4

**Description:** Reactome pathway enrichment analysis of resveratrol-interacting genes curated from the Comparative Toxicogenomics Database and Cytoscape network visualization.

**Title:** Supplementary Figure 5

**Description:** Analysis of ChIP-Atlas datasets identified E2F1 target genes bearing binding events within ±1 kb of the transcription start site.

**Title:** Supplementary Figure 6

**Description:** Flow cytometric analysis of cell cycle progression in mRTECs. RT-qPCR analysis of *E2f1* mRNA expression and ATM and CHK2 protein expression in mRTECs following the overexpression of E2f7 or E2f8. ELISA quantification of the SASP factor CCL-2.

**Title:** Supplementary Figure 7

**Description:** Relative E2F1 mRNA expression in the renal tubulointerstitium of healthy donors and patients with diabetic nephropathy via Nephroseq. Correlation between E2F1 expression levels and glomerular filtration rate in patient samples with diabetic nephropathy. via Nephroseq. Representative IHC staining and corresponding quantification of E2F1 and E2F7 protein levels in human kidney biopsy sections.
